# New 1,2,4-Triazole Derivatives with a *N*-Mannich Base Structure Based on a 4,6-Dimethylpyridine Scaffold as Anticancer Agents: Design, Synthesis, Biological Evaluation, and Molecular Modeling

**DOI:** 10.3390/ijms26146572

**Published:** 2025-07-08

**Authors:** Piotr Świątek, Teresa Glomb, Benita Wiatrak, Paulina Nowotarska, Tomasz Gębarowski, Kamil Wojtkowiak, Aneta Jezierska, Małgorzata Strzelecka

**Affiliations:** 1 Department of Medicinal Chemistry, Faculty of Pharmacy, Wroclaw Medical University, Borowska 211, 50-556 Wroclaw, Poland; teresa.glomb@umw.edu.pl (T.G.); malgorzata.strzelecka@umw.edu.pl (M.S.); 2 Department of Pharmacology, Wroclaw Medical University, J. Mikulicza-Radeckiego 2, 50-345 Wroclaw, Poland; benita.wiatrak@umw.edu.pl; 3 Department of Biostructure and Animal Physiology, Wroclaw University of Environmental and Life Sciences, Kożuchowska 1/3, 51-631 Wroclaw, Poland; paulina.nowotarska@upwr.edu.pl (P.N.); tomasz.gebarowski@upwr.edu.pl (T.G.); 4 Faculty of Chemistry, University of Wroclaw, F. Joliot-Curie 14, 50-383 Wrocław, Poland; kamil.wojtkowiak2@uwr.edu.pl

**Keywords:** dimethylpyridine, 1,2,4-triazole, *N*-Mannich base, anticancer activity, cytotoxicity, DFT, QTAIM, molecular docking

## Abstract

A series of novel *N*-Mannich bases derived from a dimethylpyridine–1,2,4-triazole hybrid was synthesized and evaluated in vitro for cytotoxic activity on several human gastrointestinal cancer cells (EPG, Caco-2, LoVo, LoVo/Dx, and HT-29). Compound **6** bearing a phenyl group at the *N*-4 position and a 4-methylphenyl piperazine moiety at the *N*-2 position of the 1,2,4-triazole-3-thione scaffold exerted good cytotoxic activities on EPG and Caco-2 cell lines, along with pronounced selectivity, showing lower cytotoxicity against normal colonic epithelial cells (CCD 841 CoTr). Further evaluation revealed the good ability of compound **6** to inhibit the efflux function of P-glycoprotein in P-gp-expressing cell lines (HT-29, LoVo, and LoVo/Dx). Moreover, compound **6** induced apoptotic cell death through a significant increase in the caspase-3 and p53 protein levels in HT-29 cells. Finally, the molecular docking method was applied to explain our experimental findings. The molecular modeling study based on Density Functional Theory (DFT) and the Quantum Theory of Atoms in Molecules (QTAIM) analysis provided insight into the geometric and electronic structure properties of the compounds.

## 1. Introduction

Given the recent updates in cancer burden estimates by the International Agency for Research on Cancer (IARC), approximately 19.98 million newly diagnosed cancer cases and 9.7 million cancer-related deaths worldwide were documented in 2022 [1]. Demographics-based predictions suggest that the annual number of new cases of cancer will reach 35 million by 2050, a 77% increase from the 2022 level. Even though the exploration for anticancer candidates started at the beginning of the preceding century, there is a persistent need to develop new antineoplastic agents with improved efficacy and minimal side effects, especially due to a rapid rise in multidrug-resistant tumors.

**Figure 1:**
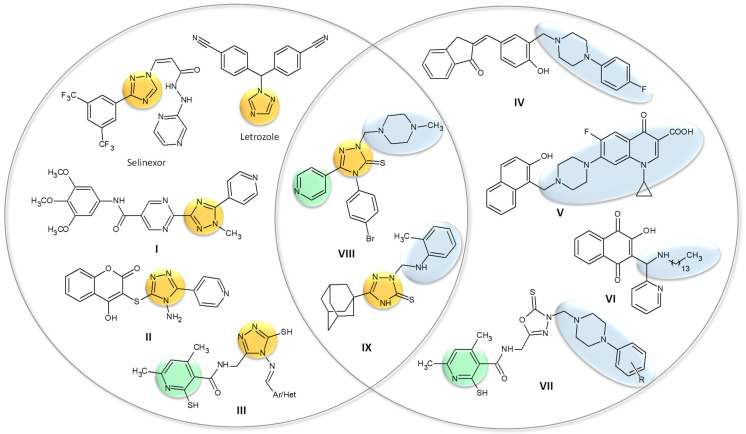
Market-available anticancer drugs containing 1,2,4-triazole and cytotoxic compounds comprising pyridine, 1,2,4-triazole, and/or *N*-Mannich base cores.

The azole scaffold is considered a privileged fragment in medicinal chemistry, particularly in the design and synthesis of anticancer drug candidates. Among all nitrogen heterocyclic systems, the importance of 1,2,4-triazoles and their derivatives is well documented due to their wide range of pharmacological properties, such as antimicrobial [2,3], analgesic [4], anti-inflammatory [5,6], antioxidant [7], and anticancer [8,9,10] activities. Owing to its electron-rich properties and structural rigidity, the triazole exerts high affinity with the biological receptors via a variety of non-covalent interactions like H-bonding, p-p conjugation, hydrophobicity, and van der Waals forces [11], giving it important pharmacological effects. Currently, several triazole analogs have been approved or are in use as drugs for the treatment of cancers for example, Letrozole, which represents a highly potent third-generation aromatase inhibitor to treat breast cancer in postmenopausal women, and Selinexor, which was explored as the first-in-class, nuclear export inhibitor used for the treatment of patients with relapsed or refractory multiple myeloma (Figure 1).

Due to the promising anticancer properties of triazole derivatives, their inclusion in the development of potent hybrid molecules is rationalized. Some of the recently reported 1,2,4-triazole-based compounds with anticancer potential are given in Figure 1. Among them, 2-(1-methyl-5-(pyridin-4-yl)-1*H*-1,2,4-triazol-3-yl)-*N*-(3,4,5-trimethoxyphenyl)-pyrimidine-5-carboxamide **I** possessed remarkable cytotoxic activity against various cancer cell lines (MCF-7, A549, A2780) with IC_50_ values ranging from 0.1 ± 0.075 to 0.26 ± 0.037 μM when compared to etoposide (IC_50_ = 1.31 ± 0.27 – 3.08 ± 0.135 μM) [12]. Another 1,2,4-triazole–pyridine hybrid **II** was more potent than the standard drug doxorubicin against HepG2 cells (IC_50_ = 1.93 μM vs. 4.45 μM, respectively), with a good selectivity and safety profile [13]. The structure–activity relationship (SAR) analysis indicated that the *N*-aminotriazole fragment is more preferable than the oxadiazole ring. Also, in our recently published work, we demonstrated that 1,2,4-triazole-Schiff base derivatives of 4,6-dimethylpyridine **III** exhibited great cytotoxic potency on several human gastrointestinal cancer cells (EPG, Caco-2, HT29, LoVo, and LoVo/Dx) with a higher selectivity index than reference drugs, 5-FU and cisplatin [14]. Further mechanistic studies showed that selected Schiff bases induced apoptotic cell death through a caspase-dependent mechanism and by regulating the p53-MDM2 signaling pathway in HT-29 cells.

On the other hand, modern organic synthesis is interested in multi-component reactions (MCRs), which offer benefits such as faster reaction times, improved yields, and repeatability [15]. The three-component Mannich reaction of a primary or secondary amine, an aldehyde component, and structurally diverse substrates containing at least one active hydrogen atom remains a valuable approach for generating C–C or N-C bonds. For the synthesis and modification of pharmacologically active molecules, the aminoalkylation of aromatic substrates utilizing the Mannich reaction is crucial for improving drug distribution in the human body or increasing the hydrophilic properties of drugs [16,17]. Studies in the literature reveal that Mannich bases derived from various skeletons exhibit several biological activities, such as antioxidant [18], anticonvulsant [19], antimicrobial [20,21], analgesic, and anti-inflammatory [22]. Moreover, aminomethylated derivatives have also been reported as potent antitumor agents (Figure 1). The phenolic Mannich base **IV** displayed significant cytotoxic properties against the Ca9-22 cell line (derived from gingival tissue) with the highest selectivity index compared with references, 5-fluorouracil, and melphalan, and it was able to strongly inhibit carbonic anhydrase (hCA) I and II isoenzymes [23]. The Mannich base derivative of 7-piperazinyl quinolones **V** showed highly potent activity against the OVCAR-3 cell line with an IC_50_ of 0.97 μM by inducing cell cycle arrest at the G2/M phase and apoptosis [24]. Investigations on Mannich bases bearing a naphthoquinone feature provided a potent anticancer agent **VI**, which was particularly active against prostate cancer cells (IC_50_ = 0.82 ± 0.04 mM for BxPC-3 and 0.67 ± 0.04 mM for PC-3 cell lines) [25]. Also, in our published work, several aminomethylated derivatives of 1,3,4-oxadiazole ring **VII** were evaluated for their growth inhibitory activity on selected cancer cell lines (A375, C32, SNB-19, MCF-7/WT, and MCF-7/Dx) [26]. The obtained results demonstrated that selected compounds have cytotoxic and proapoptotic effects on melanoma cells and can induce F-actin depolarization in a dose-dependent manner.

Combining different pharmacophores in the same unit is an attractive approach to discovering novel potent drugs. Several reports of *N*-Mannich bases of 1,2,4-triazole rings as cytotoxic agents are available in the literature (Figure 1); for example, the 4-bromophenyl derivative **VIII** incorporating a methylpiperazine moiety showed more potent cytotoxic activity against NUGC cell line (IC_50_ = 0.021 μM) compared to the standard [27], and *N*-Mannich base **IX** derived from 5-adamantyl-1,2,4-triazole-3-thione showed good cytotoxic activity against K562 and HL-60 cells through the caspase-dependent apoptosis and upregulation of Bax expression levels [28].

**Figure 2:**
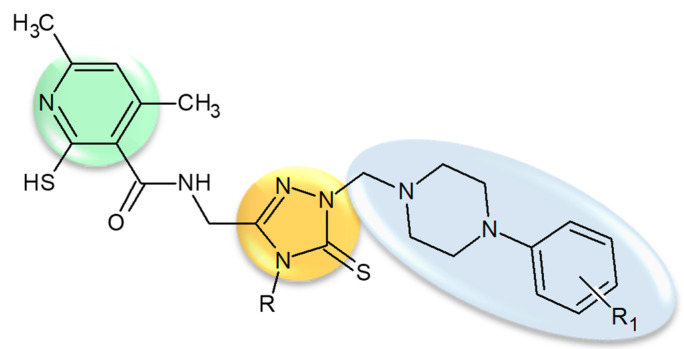
Target 1,2,4-triazole-derived *N*-Mannich bases.

In light of the aforementioned data and our continued interest in the synthesis and biological evaluation of novel, potential anticancer molecules, we designed the new compounds as a hybrid structure of a dimethylpyridine scaffold with 1,2,4-triazole-3-thione-derived *N*-Mannich base (Figure 2). Synthesized derivatives have been evaluated for their potential cytotoxicity against a panel of gastrointestinal cancer cells: gastric cancer (EPG) and colorectal cancers (Caco-2, HT29, LoVo, LoVo/Dx), cancer selectivity, and plausible mechanism of action. Finally, molecular modeling techniques were applied to explain our experimental findings. We have employed Density Functional Theory (DFT) [29,30] and the molecular docking approach [31] for this purpose. The theoretical study was divided into two parts: (i) quantum chemistry method to describe the geometric and electronic structure of the new ligands; (ii) molecular docking study to identify ligand-selected molecular target interactions.

## 2. Results and Discussion

### 2.1. Chemistry

**Scheme 1:**
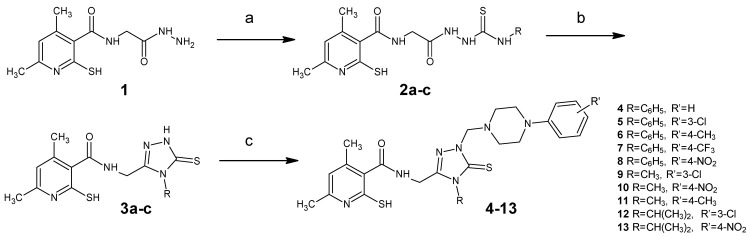
Scheme for synthesis of *N*-Mannich bases derivatives **4**–**13** (a—appropriate isothiocyanate/EtOH/reflux/12–14 h, b—NaOH/reflux/2 h/HCl, c—EtOH, HCHO, appropriate phenylpiperazine/r.t./4 h).

The synthesis of *N*-(2-hydrazinyl-2-oxoethyl)-4,6-dimethyl-2-sulfanylpyridine-3-carboxamide (**1**) was carried out using the previously published method [32]. The synthetic route leading to the formation of the target derivatives, which have not yet been described in the literature, is outlined in Scheme 1. The first step involved the condensation of compound 1 with appropriate isothiocyanates (phenyl, methyl, or isopropyl) in refluxing ethanol, followed by the cyclization of open structures **2a**–**c** to 1,2,4-triazole derivatives **3a**–**c**. Final *N*-Mannich bases **4**–**13** were formed via a convenient and efficient one-step reaction with appropriate phenylpiperazine derivatives and formaldehyde in ethanol. The spectroscopic properties of all newly obtained derivatives were determined based on spectral data analysis, such as FT-IR, ^1^H NMR, ^13^C NMR, and HR-MS. All are consistent with their predicted structures and are summarized in the experimental section.

The FT-IR spectra of compounds **4**–**13** showed peaks around 1672–1645 cm^−1^ due to the carbonyl function derived from the amide structure. Additionally, the FT-IR spectra exhibited, in the 3299–3175 cm^−1^ range, the NH band of the CONH functions. The distinctive peak in the ^1^H NMR spectrum near δ 5.00 ppm and the signal at around δ 70.00 ppm in the ^13^C NMR spectrum indicate the formation of the methylene linker characteristic of Mannich bases. Additionally, in the spectra of the final compounds, the signals of the piperazine protons, in the form of two four-proton multiplets in the range of 2.77–3.50 ppm for ^1^H NMR and two two-carbon signals in the range of 50–46 ppm for ^13^C NMR, were recorded. All NMR and FT-IR spectra are presented in Tables S1 and S2 in the Supplementary Materials. Mass spectral analysis HR-MS (ESI-MS) was carried out, and the molecular ion peaks (M + 1) were found to correlate with the corresponding calculated molecular mass, confirming the structure of synthesized molecules.

### 2.2. Biological Evaluation

#### 2.2.1. Cytotoxic Evaluation

The cytotoxic effects of ten newly synthesized *N*-Mannich bases were assessed on the normal human intestinal epithelium (CCD 841 CoTr) and cancer cell lines: gastric cancer (EPG) and four colorectal cancer lines (Caco-2, LoVo, LoVo/Dx, and HT-29). Cell viability was evaluated using the MTT assay in accordance with Annex C of ISO 10993-5 [33]. Cisplatin and 5-fluorouracil (5-FU) were included as reference drugs. The results were expressed as the cytotoxic concentration (CC_50_ ± SD) that causes a 50% reduction in viable cells (Table 1).

As shown in Table 1, the cytotoxicity of the compounds depended mainly on the substitution at the 4 position of the 1,2,4-triazole ring, as well as on the presence and electronic effect of the substituent in the phenyl ring from the phenylpiperazinyl moiety at the 2 position of the triazole. Based on the results of the general comparison between *N*-Mannich bases, it was clear that compounds bearing the isopropyl group at position *N*-4 of 1,2,4-triazole were less active than their methyl and phenyl analogs, which suggested that the bulky alkyl group may not be favored at this position. Also, the substitution variations at position *N*-2 of 1,2,4-triazole can be correlated with the activity of the *N*-Mannich bases. It was found that compounds **6** and **11** possessing *p*-methyl substituent showed the most potent cell growth inhibition activity of all tested compounds against the EPG cell line (CC_50_ for **6** = 57.70 ± 5.40 µM) and the Caco-2 cell line (CC_50_ for **11** = 57.70 ± 4.80 µM), respectively, and were more potent to the reference drugs. In addition, *N*-Mannich base **6** exhibited good cytotoxicity against Caco-2 cells (CC_50_ = 75.10 ± 5.10 µM). The compounds bearing electron-withdrawing substituents (*m*-chloro- or *p*-nitro groups), such as compounds **5**, **8**, **9**, and **10**, showed moderate anticancer activities with a CC_50_ value of 83.00 ± 4.6 µM for compound **5** against EPG, and CC_50_ in the range of 84.30 ± 6.8 – 88.60 ± 7.90 µM for compounds **8**–**10** against the Caco-2 cell line. In general, of all the investigated cancer cells, the LoVo and LoVo/Dx were the least sensitive cancer cell lines to the synthesized compounds. A significant variation in sensitivity among the remaining cell lines was observed, correlated with the chemical characteristics of the *N*-4 substituent. The presence of an aromatic ring at the *N*-4 position of 1,2,4-triazole enhances the cytotoxic effect on the EPG and HT-29 cell lines, whereas the Caco-2 cell line demonstrated the highest sensitivity to methyl analogs.

One of the major hindrances to compounds having effective anticancer activity is their toxicity towards normal cells. Thus, it is necessary to evaluate cytotoxicity on non-cancerous cells in the preliminary drug study. The CCD 841 CoTr cells were used as a human normal cell line model to investigate the safety of the *N*-Mannich bases. The results revealed that all tested compounds exhibited significantly lower cytotoxic activity towards non-cancerous cells (CC_50_ values in the range of 220.80 ± 2.40 to 486.80 ± 4.6 μM) compared to the reference drugs, cisplatin and 5-FU (CC_50_ = 14.50 ± 2.90 μM and 61.62 ± 3.70 μM, respectively). Moreover, compounds **8**, **9**, and **10** were non-toxic to normal CCD 841 CoTr cells. This finding encouraged us to calculate the selectivity index (SI = CC_50_ against non-cancer cells/CC_50_ against cancer cells), summarized in Table 1. The compounds showed various selectivity against cancer cell lines. Only a few SI values were inferior to 1. The most outstanding in selectivity was compound **6**, which showed an SI close to or above 2 for all cancer cell lines; for the EPG and Caco-2 lines, it was much higher, 6.72 and 5.16, respectively.

#### 2.2.2. Rhodamine-123 Assay

P-glycoprotein (P-gp), also referred to as multidrug resistance protein 1 (MDR1), is a member of the ATP-binding cassette (ABC) superfamily [34]. This protein plays a crucial role in transporting various molecules across cell membranes, thereby reducing their intracellular accumulation. P-gp functions as an efflux pump and is vital in the pharmacokinetics of numerous drugs, affecting their absorption, distribution, and elimination [34,35]. ABC transporters like P-gp are essential for protecting tissues from toxins and xenobiotics and play a significant role in many physiological processes [36]. However, the overexpression of P-gp is often linked to resistance to a broad range of chemotherapeutic agents. This resistance arises because P-gp actively exports drugs out of cancer cells, leading to decreased drug efficacy and lower remission and survival rates in cancer patients. As such, P-gp overexpression is a major factor contributing to the development of multidrug resistance (MDR), presenting a significant obstacle in effective cancer treatment [35]. Additionally, P-gp not only mediates the efflux of cytotoxic agents but also interferes with apoptosis pathways, including the tumor necrosis factor-related apoptosis-inducing ligand (TRAIL) and caspase-related pathways, further complicating cancer therapy [37,38,39].

**Figure 3:**
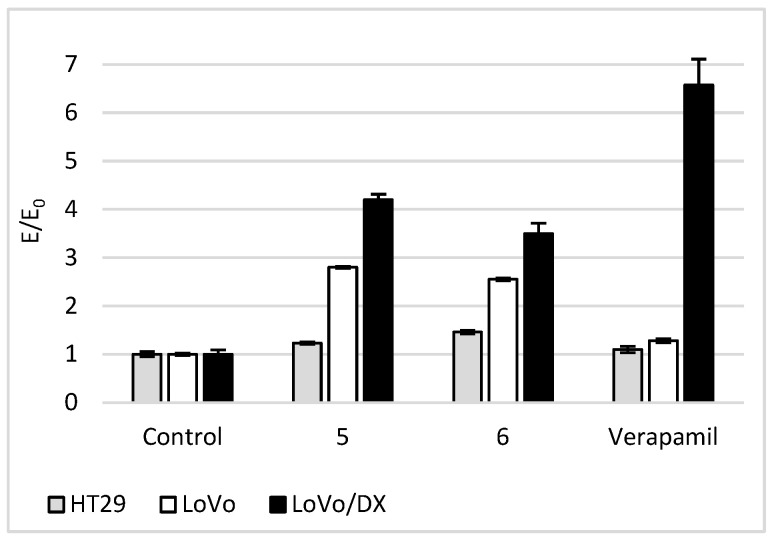
Influence of compounds **5** and **6** on rhodamine 123 accumulation in HT-29, LoVo, and LoVo/Dx cancer cells. The mean fluorescence intensity of accumulated Rh-123 dye, which means the results were normalized to control untreated cells (E is the result for the measured sample, and E_0_ is the result for the control). Verapamil was used as a positive control.

To discover potential MDR inhibitors among selected compounds (**5** and **6**), which demonstrated promising anticancer activity in gastric (EPG) and colon (Caco-2) cancer cells, a test was conducted. This test measured the intracellular accumulation of rhodamine 123 (a fluorescent P-gp substrate analog) in P-gp-expressing cell lines (HT-29, LoVo, and LoVo/Dx). Following accumulation and efflux phases, the fluorescence of rhodamine 123 (Rh-123) was quantified using spectrofluorimetry. The results are illustrated in Figure 3. Verapamil, a calcium channel blocker, served as a positive control for P-gp inhibition due to its well-established ability to inhibit P-gp activity [40]. Verapamil binds to P-gp, inhibiting its function, which increases the intracellular concentration of chemotherapeutic drugs and other P-gp substrates. This property makes verapamil a valuable tool in research for demonstrating the effectiveness of potential MDR inhibitors in reversing P-gp-mediated drug resistance [41].

In previous work, we showed that LoVo/Dx cells poorly accumulated Rh-123 compared to the other colorectal cancer cell lines used in this assay (LoVo and HT-29) [14]. The addition of verapamil to the culture increased intracellular fluorescence in LoVo/Dx cells. Compounds **5** and **6** significantly enhance the accumulation of Rh-123 in all tested cell lines. Specifically, for the HT-29 cell line, the increase was 1.23-fold and 1.46-fold, respectively; for the LoVo cell line, the increase was 2.8-fold and 2.55-fold; and for the LoVo/Dx cell line, the increase was 4.2-fold and 3.5-fold. In the case of the HT-29 and LoVo cell lines, the intensity of intracellular Rh-123 fluorescence following treatment with the tested compounds exceeded that observed with verapamil. Conversely, for the LoVo/Dx cell line, the fluorescence intensity was greater for the reference drug, verapamil, than for the tested compounds.

#### 2.2.3. Detection of Apoptosis

**Figure 4:**
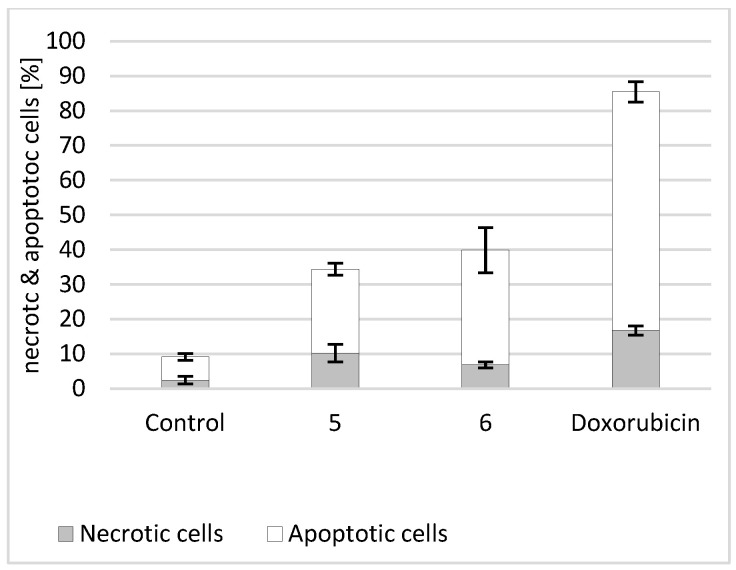
The effect of compounds **5** and **6** on the level of apoptotic and necrotic HT-29 cells after 24 h of incubation. Doxorubicin was used as a reference drug.

Most traditional chemotherapy drugs induce cancer cell death through apoptosis, a form of programmed cell death that does not cause inflammation, unlike necrosis [42,43,44]. To evaluate the impact of new compounds on apoptosis induction, an annexin-V/propidium iodide staining assay was conducted on HT-29 cells, with doxorubicin serving as the positive control (Figure 4). The tested compounds **5** and **6**, at a concentration of 1 µM, increased the number of cells in the apoptosis phase, although to a lesser extent than doxorubicin. Additionally, compound **5** caused a significant rise in the number of necrotic cells. Treatment with compound **5** resulted in more than a 3.5-fold increase in apoptotic cells compared to untreated cells, while compound **6** led to nearly a 5-fold increase.

p53 and caspase-3 are crucial proteins involved in the cellular response to anticancer compounds. p53, often referred to as the “guardian of the genome”, is a tumor suppressor protein that plays a critical role in regulating the cell cycle and inducing apoptosis in response to cellular stress and DNA damage [45,46,47]. The activation of p53 can lead to cell cycle arrest, allowing time for DNA repair, or triggering apoptosis if the damage is irreparable, thereby preventing the proliferation of potentially cancerous cells [45,46]. Many anticancer compounds aim to activate or restore the function of p53 to promote the death of cancer cells [48].

Caspase-3, a key executioner caspase in the apoptotic pathway, is activated in response to pro-apoptotic signals, including those mediated by p53 [49,50]. Once activated, caspase-3 cleaves various cellular substrates, leading to the characteristic morphological and biochemical features of apoptosis, such as DNA fragmentation, membrane blebbing, and the formation of apoptotic bodies [49].

In the context of anticancer compounds, the effectiveness of these agents is often linked to their ability to modulate the activity of p53 and caspase-3. For instance, compounds that enhance p53 activity can increase the expression of pro-apoptotic genes and proteins, subsequently leading to the activation of caspase-3 and the execution of apoptosis in cancer cells [51]. Additionally, some compounds may directly influence the activation of caspase-3, bypassing upstream signals, to induce apoptosis [49,50].

To check the effect of compound 6 on the level of p-53 and caspase-3 protein, another test was performed on HT-29 cells, taking doxorubicin as the reference drug. The level of p53 protein was nearly doubled by compound **6** compared to untreated control cells, although it was lower than the 4.23-fold increase observed with doxorubicin treatment. Conversely, the increase in caspase-3 levels was more than 15-fold compared to untreated cells, yet almost 2-fold lower than that induced by doxorubicin (Table 2).

#### 2.2.4. Effect of 6 on IL-6 and IL-1β Level

Interleukin-6 (IL-6) and Interleukin-1 beta (IL-1β) are pro-inflammatory cytokines that play different roles in the body and may respond differently to anticancer therapies [52]. IL-6 is a key factor promoting cancer progression and therapy resistance by inhibiting apoptosis of cancer cells and stimulating factors such as proliferation and angiogenesis [53]. High levels of IL-6 are associated with poor prognosis in various types of cancer, and reducing its levels or inhibiting its signaling can be therapeutically beneficial [54].

On the other hand, IL-1β is a pro-inflammatory cytokine that plays a crucial role in the inflammatory response and affects cancer cell growth, angiogenesis, and the immune response in the tumor microenvironment [55]. Increased levels of IL-1β may result from the induction of cellular stress or the death of cancer cells, leading to an inflammatory response in the body [56].

The tested compound **6** showed a statistically significant decrease in the level of the cytokine IL-6 and a slight increase in IL-1β in the HT-29 cell line (Table 3). The different effects of the anticancer compound on IL-6 and IL-1β can be explained by the different regulatory mechanisms of these cytokines and the distinct signaling pathways targeted by the therapy [52]. The reduction in IL-6 levels may be due to the direct action of the compound on pathways promoting this cytokine or its ability to inhibit inflammatory processes specific to IL-6. In contrast, the increase in IL-1β levels might result from the inflammatory response to cellular stress or apoptosis induced by the therapy, leading to activation of the immune system and increased production of IL-1β.

### 2.3. Computational Chemistry and Molecular Modeling Study

**Figure 5:**
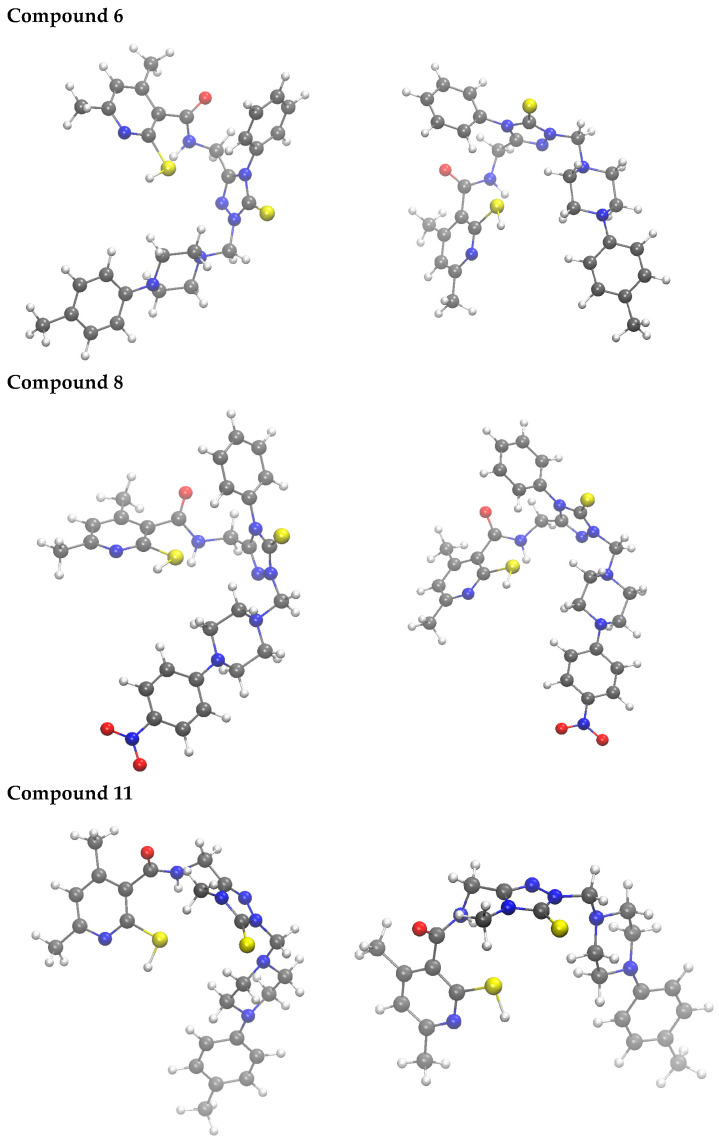
Molecular structures of *N*-Mannich **6**, **8**, and **11** obtained as a result of DFT/ωB97-XD/def2-TZVP (selected metric parameters of all compounds were gathered in Table S3) simulations in vacuo (**left**) and with solvent reaction field with water as a solvent (**right**).

Density Functional Theory (DFT) was applied to prepare the quantum-chemical description of the metric and electronic structure parameters of the studied set of *N*-Mannich bases. The simulations were performed in the gas phase and using a continuum solvation model to reproduce the polar environment influence on the molecular structure. The structures obtained as a result of the simulations in vacuo and with solvent reaction field and water as a solvent are presented in Figure 5 and Figure S1 in the SI. As is shown, the introduction of the continuum solvation does not dramatically affect the molecular structure of the studied compounds.

**Figure 6:**
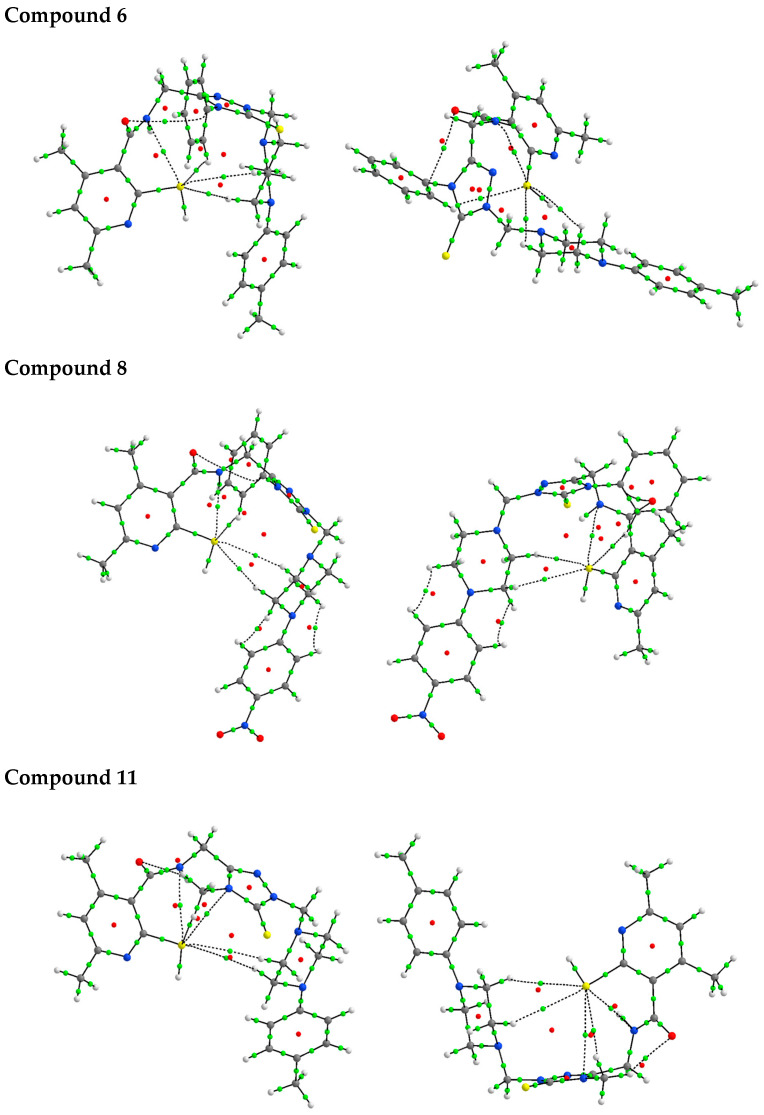
Selected QTAIM molecular graphs obtained based on results from the DFT/ωB97-XD/def2-TZVP level of theory in vacuo for compounds **6**, **8**, and **11**. The dotted line indicates the presence of a non-covalent interaction. The green and red spheres are Bond and Ring Critical Points (BCPs and RCPs), respectively. The complete set of graphs is presented in the SI.

We have chosen compounds denoted as **6**, **8**, and **11** to present their molecular structures, because they exhibit promising properties based on biological activity assays as well as a molecular docking study. Next, the partial atomic charge distribution in the core part of the studied set of *N*-Mannich bases was analyzed based on Quantum Theory of Atoms in Molecules (QTAIM). The theory made it possible to study the electron density distribution as well as the topology of the investigated compounds. In Scheme S1, the core part of the compounds is presented with atom numbering prepared for the current study. It was of interest to see quantitatively how the presence of various substituents affects the electron density distribution upon the introduction of various substituents. In Table S4, the data are presented. As is shown, the electron density changes are visible, and depending on the substituent, we can notice a slight decrease or an increase in the partial atomic charge values. Such a study is useful in further design of similar molecules, because for the most biologically active compounds we can monitor changes in the atomic charges distribution in the core part and further draw conclusions concerning the targeted substitution. The study is completed by the electron density value and its Laplacian at selected Bond Critical Points (BCPs), see Table S5. We have chosen the pyridine ring as an important chemical part present in all investigated molecules. As is shown, the electron density values at selected BCPs are very similar. We could notice only slight changes. In Figure 6, Figures S2 and S3, molecular graphs derived from the QTAIM theory are presented.

**Figure 7:**
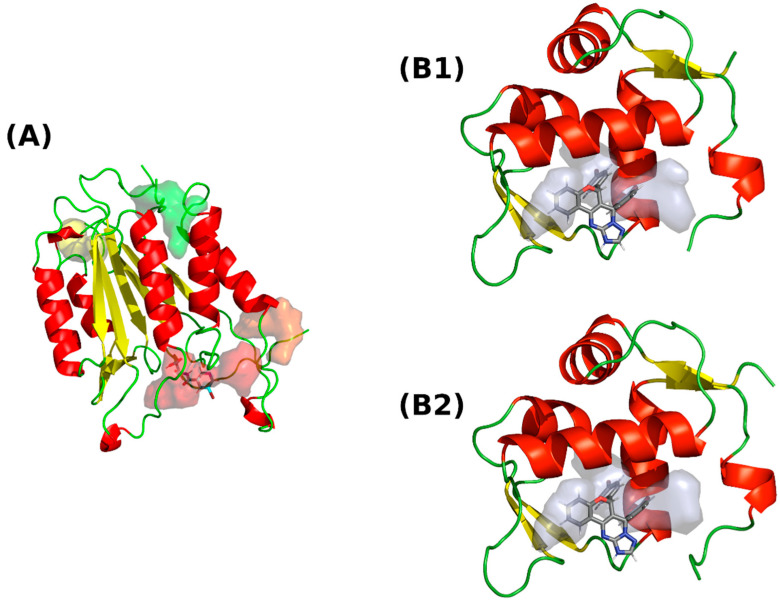
Visualization of the studied enzymes (**A**)—caspase-3, (**B1**)—MDM-2 (model 1), and (**B2**) MDM-2 (model 2) with their native ligand structures as well as their binding pockets (transparent surfaces) calculated with PyVol.

The performed topological analysis revealed the network of non-covalent interactions responsible for the spatial arrangement of atoms in the studied compounds. In the next step, to elucidate the underlying factors responsible for the ligand–protein interactions, a variety of molecular modeling techniques were applied, including classical mechanics-based calculations of binding site volume, electrostatic potential of the protein, and molecular volume (MV) calculations of the examined inhibitors. Finally, a molecular docking study was carried out to estimate the binding affinity as well as the position of the *N*-Mannich bases in the binding pocket. Based on the results of the biological activity assays, caspase-3 and MDM2 were selected for molecular docking studies. MDM2, a well-known negative regulator of p53, was included in the study due to its critical role in the inactivation of the p53 pathway through direct binding to p53, which inhibits its transcriptional activity and promotes its degradation via the ubiquitin–proteasome pathway. In Figure 7, the structures of native ligands and proteins, as well as their binding sites, are presented. It is worth underlining that two different models of the MDM-2 protein were prepared to study the interactions of chosen ligands with the binding pocket. For the (B2) model, the *N*-terminal loops were modeled, whereas in the model (B1) the experimentally obtained structure of the protein was used. The analysis of binding pockets in caspase-3 revealed four distinct regions accessible for the ligand. In this case, the nicotinic acid aldehyde binds to the volumetrically largest pocket (630 Å3, marked by transparent red surface). In case of (B1), the binding pocket size was estimated to be 414 Å3, and rebuilding of the *N*-terminal loop region (B2 model) enlarged it to 441 Å3.

**Figure 8:**
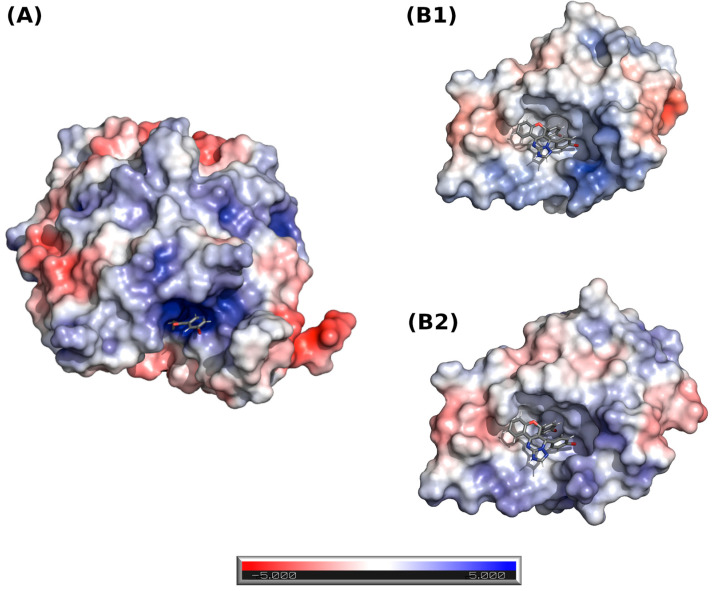
The electrostatic potential (ESP) visualization of the studied enzyme models with their native ligands. Enzymes (**A**)—caspase-3, (**B1**)—MDM-2 (model 1), and (**B2**)—MDM-2 (model 2). The red color indicates regions with an excess of negative charge, while blue indicates those with a positive charge.

Moreover, the analysis of the electrostatic potential (ESP) maps (see Figure 8) indicates the importance of localizing the negative charge on ligand structures—the binding site of the caspase-3 model (A) shows positive ESP values, hence structures having electro-negative groups (which can act as Lewis bases) will more easily interact with these enzymes. Similar observations can be made for (B1) and (B2) models. In both cases, the ESP values are rather smaller in magnitude than those found for (A); however, most of the attractive components to the interaction with this protein will still arise due to Coulombic forces between the positively charged binding pockets and the negative charge located on the ligand molecule.

**Figure 9:**
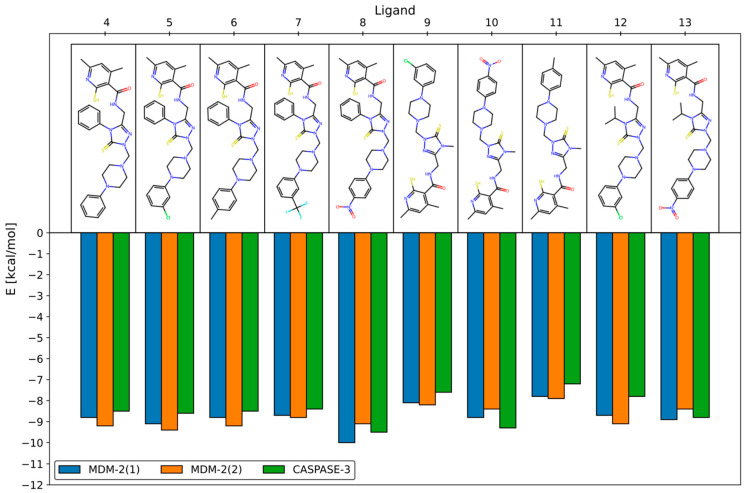
Molecular docking results obtained for the set of studied *N*-Mannich bases. The host macromolecules are MDM-2 (models 1 and 2) and caspase-3, respectively.

The ESP graphs identify how the specificity of the two binding sites differs. In the case of (A), the BS is characterized by a small passage to the bulk from the binding cavity, while the entire BS of MDM-2 is exposed and easily accessible to molecules from the environment. Figure 9 and Table S6 show the results obtained from the molecular docking experiment.

**Figure 10:**
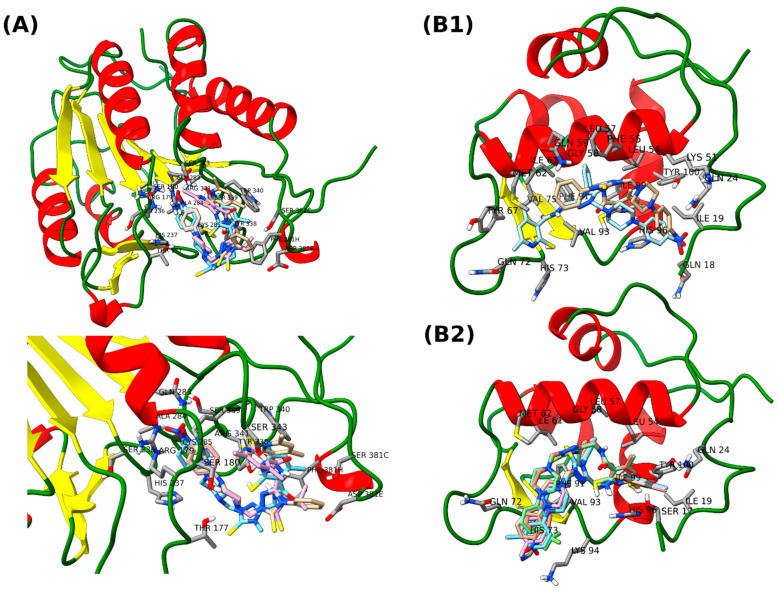
Visualization of selected positions of ligand–protein complexes, (**A**)—caspase-3 with compounds denoted as **8**, **10**, and **13**, (**B1**)—MDM-2 (model 1) with **8**, and (**B2**) MDM-2 (model 2) with **4**, **5**, **6**, **7**, and **8** positions of ligands that obtained the most favorable binding affinities in the docking experiment. Coloration of the carbon atoms of the ligands: (in order of increasing number of ligands) tan, light blue, pink, light green, light brown.

Generally, the obtained binding affinities for all of the investigated compounds are within the error margin of the molecular docking method. Nonetheless, it can be seen, that in the case of MDM-2(1) model the most favorable binding affinity (BA) was obtained by **8**–MDM-2 complex, whereas for MDM-2(2) model, the results did not differ significantly from each other within the group of ligands **4**, **5**, **6**, **7**, and **8**. Interestingly, all of the molecules of the group assumed a similar position in the binding pocket of the MDM-2(2) model (see (B2), Figure 10). That tendency can be explained based on the presence of a phenyl substituent and, in consequence, the larger molecular volume of these molecules. In general, lower binding affinities were obtained for complexes with ligands of larger molecular volumes for both MDM-2 models. Another factor associated with the electronegativity of the functional groups attached to the phenyl ring seems to play a less important role for MDM-2 complexes (compare BA for **6** and **11**). The latter was more important in the case of caspase-3 complexes, where the BS is more positively charged (Figure 8). In line with this, the most pronounced BA values were obtained for caspase-3 complexes of **8**, **10**, and **13**, where an electronegative NO_2_ group is present. In the case of caspase-3 complexes, the compounds denoted as **9**, **11**, and **12** tend to exhibit lower (in magnitude) values of BA because of the modification in their structure (the lack of the aromatic ring attached to the triazole moiety as well as the NO_2_ substituent). It is worth to emphasize that, there is no clear preference for ligands with lower/larger magnitude of molecular volume (MV)—for instance, the BA for caspase-3 complex with compound denoted as **10** was equal to −9.3 kcal/mol and its MV equals to 503 Å, whereas complex with **7** (MV equal to 577 Å) acquired BA of only −8.4 kcal/mol.

**Figure 11:**
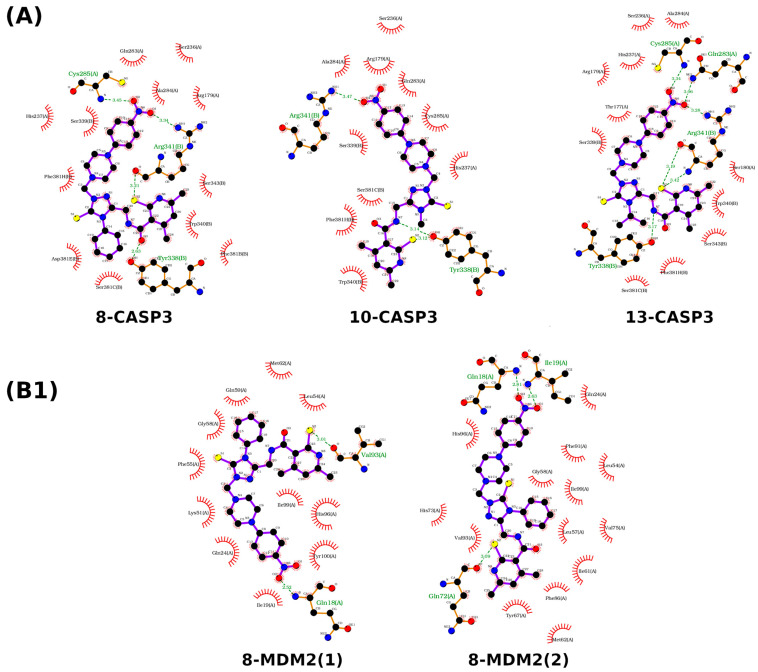
Diagrams of the interactions of selected ligand–protein complexes for the model enzymes (**A**)—caspase-3 and (**B1**)—MDM-2 (model 1). Hydrophobic interactions were indicated by the presence of red combs, while hydrogen bonds were indicated by green pathways between the H-bond donor and acceptor atoms.

**Figure 12:**
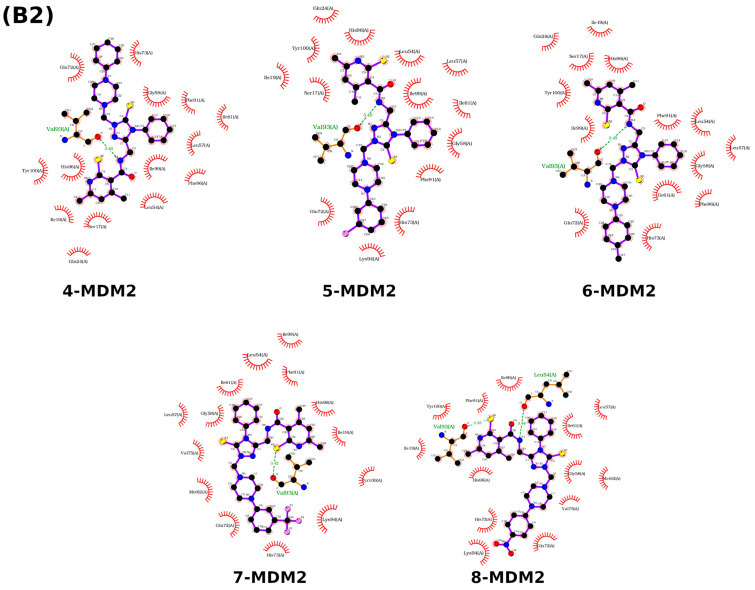
Diagrams of interactions of selected ligand–protein complexes for model enzyme (**B2**)—MDM-2 (model 2). Hydrophobic interactions are indicated by the presence of red combs, while hydrogen bonds are indicated by green paths between the H-bond donor and acceptor atoms.

Detailed analysis of the above-mentioned ligands was facilitated by inspection of the interaction diagrams generated using the LigPlot+ software v.2.2 (see Figure 11 and Figure 12). In Figure 11, the interactions between the caspase-3 enzyme and the first of two MDM-2 models of the protein are depicted. Starting with the (A) complexes, it can be seen that all of the analyzed ligands possess an electronegative NO_2_ group (aromatic ring attached to the triazole ring was not a mandatory molecular feature to obtain a preferable binding affinity of the studied complex, as was elaborated earlier). Notably, in the case of all studied compounds, there was a high preference in the formation of the hydrogen bond (HB) between NO_2_ and Cys285, Arg341, Gln283, Gln18, and Ile19 residues. The formation of two such HBs was observed for the **8**–caspase-3 complex (with Cys285 and Arg341), whereas one (with Arg341) and three formed in the case of the **10**–caspase-3 and **13**–caspase-3 complexes, respectively. In addition, in each of the complexes, there is also a hydrogen bond between the thiol group (S2-H) and the oxygen of Arg341 (ligands: **8**, **13**) or the oxygen of the hydroxyl group of Tyr338 in the **10**–caspase-3 complex. In **10**–caspase-3 and **13**–caspase-3, one can notice the presence of HBs, where the N7 nitrogen atom acts as a Lewis acid. In the case of the **8**–caspase-3 complex, the HB forms between the Tyr338 hydroxyl group hydrogen and the carbonyl oxygen (O3) of the ligand molecule.

In case of the first model of MDM-2, one can notice the presence of different interactions characteristic of the two studied **8**-MDM-2 binding modes. This example is a striking evidence for the key tenet in the host-guest interactions of structural chemistry—the model of induced fit, rather than possessing only one favorable conformations, ligands often can assume entirely different poses with negligibly small penalty on the binding affinity (the difference between these two conformations is ca. 0.1 kcal/mol, if the obtained AutoDockFR 1.2 scoring function values are compared). In the case of the first conformation, the intermolecular hydrogen bond forms between the NO_2_ group of compound **8** and the NH group of Gln18, as well as between S2-H and the carbonyl oxygen from Val93. For the second conformation, the HBs were formed between the thiol group (S2-H) and carbonyl oxygen of Gln72, as well as between NO_2_ oxygens (of **8**) and amine groups of Gln18 and Ile19. An inspection of Figure 12 provides a detailed overview of the binding modes of selected ligands with the MDM-2 enzyme containing modeled loops regions. It can be seen that all of the examined compounds contain the same structural fragment—they only differ by substituents at the phenyl ring.

For **4**–MDM-2 and **6**–MDM-2 complexes, one can observe the formation of HB between the N4-H from the amide group of the ligand with the carbonyl oxygen of Val93 amino acid. In the case of the **5**–MDM-2 complex, there exists an additional hydrogen bond, where the chlorine atom acts as a Lewis base and the amine group of Gln72 is a hydrogen donor. The formation of additional HBs was observed for the **7**–MDM-2 and **8**–MDM-2 complexes. One is characterized by CF_3_ and NO_2_ groups, acting as electron donors to the positively charged side chain amine group of Lys94, whereas in the case of the second, the S-H group interacts with the carbonyl oxygen of Val93. In both complexes, the formation of a third HB was observed between the nitrogen of the amide group of compounds **7** and **8** and the carbonyl oxygen of the Leu54 residue.

## 3. Materials and Methods

### 3.1. Chemistry

#### 3.1.1. Instruments and Chemicals

All solvents, reagents, and chemicals used during experiments described in this paper were delivered by commercial suppliers (Alchem, Wrocław, Poland; Chemat, Gdańsk, Poland; Archem, Łany, Poland) and were used without further purification. Any dry solvents were received due to standard procedures. Reaction progress was monitored by Thin-Layer Chromatography (TLC) technique, on TLC plates made of 60–254 silica gel, and was visualized by UV light at 254/366 nm. Melting points of final compounds were determined on the Electrothermal Mel-Temp 1101D apparatus (Cole-Parmer, Vernon Hills, IL, USA) using the open capillary method, and were uncorrected. ^1^H NMR (600 MHz) and ^13^C NMR (151 MHz) spectra were recorded using a Bruker 600 MHz NMR spectrometer (Bruker Analytische Messtechnik GmbH, Rheinstetten, Germany) in DMSO-*d*_6_, with tetramethylsilane (TMS) as an internal reference. Chemical shifts (δ) were reported in ppm, and multiplicities of NMR signals are designated as s (singlet), d (doublet), t (triplet), q (quartet), h (hexaplet), and m (multiplet, for unresolved lines). To record and read spectra, the MestReNova v.15.0.0 (Mestrelab Research, S.L.U., Santiago de Compostela, Spain) program was used. FT-IR spectra were measured on a Nicolet iS50 FT-IR Spectrometer (Thermo Fisher Scientific, Waltham, MA, USA). Frequencies were reported in cm^−1^. All samples were solid, and spectra were read by OMNIC Spectra 2.0 (Thermo Fisher Scientific, Waltham, MA, USA). Mass spectra (MS) were recorded using the Bruker Daltonics Compact ESI-Mass Spectrometer (Bruker Daltonik, GmbH, Bremen, Germany), operating in the positive ion mode. The analyzed compounds were dissolved in methanol, methanol/water, or chloroform/methanol mixture. Theoretical monoisotopic masses of ions were calculated (calcd.) using Bruker Compass Data Analysis 4.2 software (Bruker Daltonik GmbH, Bremen, Germany).

#### 3.1.2. Preparation and Experimental Properties of Compounds **2**–**13**

The synthesis protocols and experimental data for compound **1** were already reported—hydrazide [32].

##### General Procedure for Preparation of Compounds **2a**–**c**

A mixture of 5 mmol of hydrazide **1** (1 eq.) and 15 mL of absolute ethanol was stirred for a few minutes. Then, 7.5 mmol of phenyl, methyl, or isopropyl isothiocyanate (1.5 eq.) was added to the mixture, stirred, and refluxed for 12–14 h. The course of the reaction was controlled by TLC. The precipitate was filtered off and washed with ethanol. The crude product was purified by crystallization in methanol.

**2a** 4,6-dimethyl-*N*-{2-oxo-2-[2-(phenylcarbamothioyl)hydrazinyl]ethyl}-2-sulfanylpyridine-3-carboxamide.

White solid, yield: 67%, m.p. 242–245 °C. ^1^H NMR (600 MHz, DMSO-*d*_6_) δ ppm: 13.58 (s, 1H, SH), 10.52 (s, 1H, NH), 9.79 (s, 1H, NH), 9.13 (s, 1H, NH), 8.99 (s, 1H, NH), 7.56 (d, 2H, ArH, *J* = 6 Hz) 7.32 (t, 2H, ArH, *J* = 12 Hz), 7.15 (t, 1H, ArH, *J* = 12 Hz), 6.64 (s, 1H, CH_pyridine_), 3.95 (d, 2H, CH_2_, *J* = 6 Hz), 2.33 (s, 3H, CH_3_), 2.13 (s, 3H, CH_3_). ^13^C NMR (151 MHz, DMSO-*d*_6_) δ ppm: 180.76, 173.29, 168.71, 168.61, 149.25, 147.47, 139.39, 136.38, 128.57, 125.50, 125.15, 116.21, 42.71, 19.46, 18.80. FT-IR (selected lines, γ_max_, cm^−1^): 3247, 3162 (NH), 2982 (C-H aliph), 1672, 1622 (C=O). HR-MS (ESI-MS) (*m*/*z*): calcd. for C_17_H_19_N_5_O_2_S_2_ [M + H]^+^: 390.1053; found: 390.1051.

**2b** 4,6-dimethyl-*N*-{2-oxo-2-[2-(methylcarbamothioyl)hydrazinyl]ethyl}-2-sulfanylpyridine-3-carboxamide.

White solid, yield: 81%, m.p. 230–235 °C. ^1^H NMR (600 MHz, DMSO-*d*_6_) δ ppm: 13.52 (s, 1H, SH), 10.18 (s, 1H, NH), 9.36 (s, 1H, NH), 8.75 (t, 1H, NH, *J* = 12 Hz), 7.64 (d, 1H, NH, *J* = 6 Hz), 6.62 (s, 1H, CH_pyridine_), 3.89 (d, 2H, CH_2_, *J* = 6 Hz), 2.87 (d, 3H, CH_3_, *J* = 6 Hz), 2.32 (s, 3H, CH_3_), 2.13 (s, 3H, CH_3_). ^13^C NMR (151 MHz, DMSO-*d*_6_) δ ppm: 182.61, 173.45, 168.79, 168.14, 149.05, 147.43, 136.51, 116.04, 42.34, 31.27, 19.47, 18.75. FT-IR (selected lines, γ_max_, cm^−1^): 3369, 3161 (NH), 2967 (C-H aliph), 1676, 1658 (C=O). HR-MS (ESI-MS) (*m*/*z*): calcd. for C_12_H_17_N_5_O_2_S_2_ [M + H]^+^: 328.0896; found: 328.0894.

**2c** 4,6-dimethyl-*N*-{2-oxo-2-[2-(propan-2-ylcarbamothioyl)hydrazinyl]ethyl}-2-sulfanylpyridine-3-carboxamide.

Light yellow solid, yield: 60%, m.p. 236–240 °C. ^1^H NMR (600 MHz, DMSO-*d*_6_) δ ppm: 13.54 (s, 1H, SH), 10.23 (s, 1H, NH), 9.27 (s, 1H, NH), 9.00 (s, 1H, NH), 8.65 (s, 1H, NH), 6.62 (s, 1H, CH_pyridine_), 4.35 (h, 1H, CH, *J* = 30 Hz), 3.84 (d, 2H, CH_2_, *J* = 6 Hz), 2.32 (s, 3H, CH_3_), 2.12 (s, 3H, CH_3_), 1.09 (d, 6H, 2xCH_3_, *J* = 12 Hz). ^13^C NMR (151 MHz, DMSO-*d*_6_) δ ppm: 173.44, 168.58, 168.43, 167.14, 149.13, 148.90, 147.26, 136.82, 136.41, 116.04, 46.10, 42.77, 41.87, 22.35, 19.40, 18.74. FT-IR (selected lines, γ_max_, cm^−1^): 3330, 3144 (NH), 2974 (C-H aliph), 1718, 1655 (C=O). HR-MS (ESI-MS) (*m*/*z*): calcd. for C_14_H_21_N_5_O_2_S_2_ [M + H]^+^: 356.1209; found: 356.1206.

##### General Procedure for Preparation of Compounds **3a**–**c**

A solution of 1 mmol of appropriate compound **2a**, **2b**, or **2c** in 10 mL of 10% sodium hydroxide was stirred and refluxed for 2 h. The reaction was then cooled with ice and acidified dropwise with 10% hydrochloric acid. The residue was left overnight and then filtered, dried, and crystallized from methanol.

**3a** 4,6-dimethyl-*N*-[(4-phenyl-5-thioxo-4,5-dihydro-1*H*-1,2,4-triazol-3-yl)methyl]-2-sulfanylpyridine-3-carboxamide.

White solid, yield: 70%, m.p. 289–292 °C decomp. ^1^H NMR (600 MHz, DMSO-*d*_6_) δ ppm: 13.26 (s, 1H, SH), 8.60 (t, 1H, NH, *J* = 12 Hz), 7.57–7.50 (m, 5H, ArH), 6.46 (s, 1H, CH_pyridine_), 4.20 (d, 2H, CH_2_, *J* = 6 Hz), 2.26 (s, 3H, CH_3_), 1.90 (s, 3H, CH_3_). ^13^C NMR (151 MHz, DMSO-*d*_6_) δ ppm: 174.30, 168.35, 167.08, 149.83, 148.33, 146.69, 136.81, 133.79, 129.94, 129.80, 128.65, 115.26, 35.27, 19.42, 18.68. FT-IR (selected lines, γ_max_, cm^−1^): 3339, 3193 (N-H), 2951 (C-H aliph.), 1645 (C=O). HR-MS (ESI-MS) (*m*/*z*): calcd. for C_17_H_17_N_5_OS_2_ [M + H]^+^: 372.0947; found: 372.0940.

**3b** 4,6-dimethyl-*N*-[(4-methyl-5-thioxo-4,5-dihydro-1*H*-1,2,4-triazol-3-yl)methyl]-2-sulfanylpyridine-3-carboxamide.

Light yellow solid, yield: 85%, m.p. 282–285 °C decomp. ^1^H NMR (600 MHz, DMSO-*d*_6_) δ ppm: 13.60 (s, 1H, NH), 13.31 (s, 1H, SH), 8.71 (t, 1H, NH, *J* = 12 Hz), 6.51 (s, 1H, CH_pyridine_), 4.48 (d, 2H, CH_2_, *J* = 6 Hz), 3.56 (s, 3H, CH_3_), 2.28 (s, 3H, CH_3_), 2.07 (s, 3H, CH_3_). ^13^C NMR (151 MHz, DMSO-*d*_6_) δ ppm: 174.28, 167.59, 167.16, 150.26, 148.39, 146.55, 137.08, 115.32, 34.37, 30.87, 19.50, 18.67. FT-IR (selected lines, γ_max_, cm^−1^): 3193, 3036 (N-H), 2938 (C-H aliph.), 1655 (C=O). HR-MS (ESI-MS) (*m*/*z*): calcd. for C_12_H_15_N_5_OS_2_ [M + H]^+^: 310.0791; found: 310.0779.

**3c** 4,6-dimethyl-*N*-{[4-(propan-2-yl)-5-thioxo-4,5-dihydro-1*H*-1,2,4-triazol-3-yl]methyl}-2-sulfanylpyridine-3-carboxamide.

Yellow solid, yield: 75%, m.p. 300–305 °C decomp. ^1^H NMR (600 MHz, DMSO-*d*_6_) δ ppm: 13.28 (s, 1H, SH), 8.72 (t, 1H, NH, *J* = 12 Hz), 6.50 (s, 1H, CH_pyridine_), 4.86 (h, 1H, CH, *J = 18 Hz*), 4.49 (d, 2H, CH_2_, *J* = 6 Hz), 2.28 (s, 3H, CH_3_), 2.09 (s, 3H, CH_3_), 1.55 (d, 6H, 2xCH_3_, *J* = 12 Hz). ^13^C NMR (151 MHz, DMSO-*d*_6_) δ ppm: 174.46, 171.64, 167.30, 149.83, 148.16, 146.64, 136.94, 115.27, 48.75, 20.03, 19.47, 18.67. FT-IR (selected lines, γ_max_, cm^−1^): 3297, 3034 (N-H), 2952 (C-H aliph), 1662 (C=O). HR-MS (ESI-MS) (*m*/*z*): calcd. for C_14_H_19_N_5_OS_2_ [M + H]^+^: 338.1104; found: 338.1093.

##### General Procedure for Preparation of Compounds **4**–**13**

0.16 mL of 36% formaldehyde was added to a solution of 1 mmol of 1,2,4-triazole derivative (**3a**, **3b**, or **3c**) in 30 mL of ethanol. The obtained mixture was stirred at room temperature for several minutes. Then, 1 mmol of the appropriate piperazine derivative was added to the flask. The resulting mixture was stirred for 4 h at room temperature and then left overnight. The obtained precipitate was filtered off and allowed to dry, then the obtained product was crystallized from methanol.

**4** 4,6-dimethyl-*N*-[(4-phenyl-5-thioxo-2-{[4-phenylpiperazinyl]methyl}-4,5-dihydro-1*H*-1,2,4-triazol-3-yl)methyl]-2-sulfanylpyridine-3-carboxamide.

Light yellow solid, yield: 71%, m.p. 200–203 °C decomp. ^1^H NMR (600 MHz, DMSO-*d*_6_) δ ppm: 13.26 (s, 1H, SH), 8.65 (t, 1H, NH, *J* = 12 Hz), 7.59–7.53 (m, 5H, ArH), 7.21–7.19 (m, 2H, ArH), 6.94–6.93 (m, 2H, ArH), 6.79–6.77 (m, 1H, ArH), 6.46 (s, 1H, CH_pyridine_), 5.15 (s, 2H, CH_2_), 4.24 (d, 2H, CH_2_, *J* = 6 Hz), 3.14 (m, 4H, CH_2-piperazine_), 2.91 (m, 4H, CH_2-piperazine_), 2.26 (s, 3H, CH_3_), 1.93 (s, 3H, CH_3_). ^13^C NMR (151 MHz, DMSO-*d*_6_) δ ppm: 174.35, 169.39, 167.00, 151.58, 148.53, 146.72, 136.83, 134.28, 129.86, 129.35, 128.67, 119.47, 116.12, 115.22, 69.11, 50.36, 48.82, 35.14, 19.40, 18.68. FT-IR (selected lines, γ_max_, cm^−1^): 3377 (N-H), 2835 (C-H aliph.), 1668 (C=O). HR-MS (ESI-MS) (*m*/*z*): calcd. for C_28_H_31_N_7_OS_2_ [M + H]^+^: 546.2104; found: 546.2128.

**5** 4,6-dimethyl-*N*-[(4-phenyl-5-thioxo-2-{[4-(3-chlorophenyl)piperazinyl]methyl}-4,5-dihydro-1*H*-1,2,4-triazol-3-yl)methyl]-2-sulfanylpyridine-3-carboxamide.

Light yellow solid, yield: 82%, m.p. 214–218 °C decomp. ^1^H NMR (600 MHz, DMSO-*d*_6_) δ ppm: 13.25 (s, 1H, SH), 8.64 (t, 1H, NH, *J* = 6 Hz), 7.58–7.52 (m, 5H, ArH) 7.21–7.18 (m, 1H, ArH), 6.93–6.89 (m, 2H, ArH), 6.78–6.77 (m, 1H, ArH), 6.45 (s, 1H, CH_pyridine_), 5.14 (s, 2H, CH_2_), 4.23 (d, 2H, CH_2_, *J* = 6 Hz), 3.18 (m, 4H, CH_2-piperazine_), 2.88 (m, 4H, CH_2-piperazin_e), 2.25 (s, 3H, CH_3_), 1.91 (s, 3H, CH_3_). ^13^C NMR (151 MHz, DMSO-*d*_6_) δ ppm: 174.34, 169.37, 167.06, 152.77, 148.57, 148.35, 146.74, 136.82, 134.27, 134.25, 130.87, 130.09, 129.86, 128.66, 118.64, 115.22, 115.12, 114.40, 69.06, 50.19, 48.21, 35.14, 19.43, 18.69. FT-IR (selected lines, γ_max_, cm^−1^): 3196 (N-H), 2828 (C-H aliph.), 1645 (C=O). HR-MS (ESI-MS) (*m*/*z*): calcd. for C_28_H_30_ClN_7_OS_2_ [M + H]^+^: 580.1715; found: 580.1732.

**6** 4,6-dimethyl-*N*-[(4-phenyl-5-thioxo-2-{[4-(4-methylphenyl)piperazinyl]methyl}-4,5-dihydro-1*H*-1,2,4-triazol-3-yl)methyl]-2-sulfanylpyridine-3-carboxamide.

White solid, yield: 74%, m.p. 205–208 °C decomp. ^1^H NMR (600 MHz, DMSO-*d*_6_) δ ppm: 13.25 (s, 1H, SH), 8.65 8.64 (t, 1H, NH, *J* = 12 Hz), 7.58–7.52 (m, 5H, ArH), 7.01–7.00 (m, 2H, ArH), 6.83–6.81 (m, 2H, ArH), 6.45 (s, 1H, CH_pyridine_), 5.13 (s, 2H, CH_2_), 4.24 (d, 2H, CH_2_, *J* = 6 Hz), 3.07 (m, 4H, CH_2-piperazine_), 2.89 (m, 4H, CH_2-piperazine_), 2.25 (s, 3H, CH_3_), 2.19 (s, 3H, CH_3_), 1.92 (s, 3H, CH_3_). ^13^C NMR (151 MHz, DMSO-*d*_6_) δ ppm:174.34, 169.36, 167.06, 149.50, 148.52, 148.34, 146.72, 136.83, 134.28, 130.08, 129.86, 129.82, 129.79, 128.66, 128.28, 116.39, 115.22, 69.11, 50.36, 49.31, 35.14, 20.53, 19.43, 18.68. FT-IR (selected lines, γ_max_, cm^−1^): 3224 (N-H), 2833 (C-H aliph.), 1652 (C=O). HR-MS (ESI-MS) (*m*/*z*): calcd. for C_29_H_33_N_7_OS_2_ [M + H]^+^: 560.2261; found: 560.2283.

**7** 4,6-dimethyl-*N*-[(4-phenyl-5-thioxo-2-{[4-(3-trifluoromethylphenyl)piperazinyl-]methyl}-4,5-dihydro-1*H*-1,2,4-triazol-3-yl)methyl]-2-sulfanylpyridine-3-carboxamide.

White solid, yield: 67%, m.p. 222–226 °C decomp. ^1^H NMR (600 MHz, DMSO-*d*_6_) δ ppm: 13.26 (s, 1H, SH), 8.64 (t, 1H, NH, *J* = 12 Hz), 7.59–7.50 (m, 5H ArH), 7.42–7.40 (m, 1H ArH), 7.23–7.22 (m, 1H, ArH), 7.16 (m, 1H, ArH), 7.08–7.06 (m, 1H, ArH), 6.45 (s, 1H, CH_pyridine_), 5.16 (s, 2H, CH_2_), 4.24 (d, 2H, CH_2_, *J* = 6 Hz), 3.24 (m, 4H, CH_2-piperazine_), 2.91 (m, 4H, CH_2-piperazine_), 2.26 (s, 3H, CH_3_), 1.92 (s, 3H, CH_3_). ^13^C NMR (151 MHz, DMSO-*d*_6_) δ ppm: 174.33, 169.39, 168.36, 167.06, 151.75, 149.84, 148.57, 148.36, 146.73, 136.82, 134.26, 133.80, 130.43, 129.87, 129.79, 128.66, 125.80, 123.99, 119.47, 115.21, 111.49, 69.05, 50.20, 48.16, 35.15, 19.43, 18.68. FT-IR (selected lines, γ_max_, cm^−1^): 3205 (N-H), 2830 (C-H aliph.), 1646 (C=O). HR-MS (ESI-MS) (*m*/*z*): calcd. for C_29_H_30_F_3_N_7_OS_2_ [M + H]^+^: 614.1978; found: 614.2005.

**8** 4,6-dimethyl-*N*-[(4-phenyl-5-thioxo-2-{[4-(4-nitrophenyl)piperazinyl]methyl}-4,5-dihydro-1*H*-1,2,4-triazol-3-yl)methyl]-2-sulfanylpyridine-3-carboxamide.

Yellow solid, yield: 76%, m.p. 231–234 °C decomp. ^1^H NMR (600 MHz, DMSO-*d*_6_) δ ppm: 13.25 (s, 1H, SH), 8.64 (t, 1H, NH, *J* = 12 Hz), 8.04 (d, 2H, ArH, *J* = 6 Hz), 7.57–7.51 (m, 5H, ArH), 7.03 (d, 2H, ArH, *J* = 6 Hz), 6.43 (s, 1H, CH_pyridine_), 5.16 (s, 2H, CH_2_), 4.22 (d, 2H, CH2, *J* = 6 Hz), 3.50 (m, 4H, CH_2-piperazine_), 2.89 (m, 4H, CH_2-piperazine_), 2.26 (s, 3H, CH_3_), 1.88 (s, 3H, CH_3_). ^13^C NMR (151 MHz, DMSO-*d*_6_) δ ppm: 174.31, 169.38, 167.02, 155.08, 148.60, 148.37, 146.70, 137.26, 136.79, 134.24, 130.08, 129.84, 128.65, 126.21, 115.19, 113.07, 68.94, 50.04, 46.73, 35.12, 19.40, 18.67. FT-IR (selected lines, γ_max_, cm^−1^): 3175 (N-H), 2844 (C-H aliph.), 1645 (C=O). HR-MS (ESI-MS) (*m*/*z*): calcd. for C_28_H_30_N_8_O_3_S_2_ [M + H]^+^: 591.1955; found: 519.1978.

**9** 4,6-dimethyl-*N*-[(4-methyl-5-thioxo-2-{[4-(3-chlorophenyl)piperazinyl]methyl}-4,5-dihydro-1*H*-1,2,4-triazol-3-yl)methyl]-2-sulfanylpyridine-3-carboxamide.

White solid, yield: 69%, m.p. 230–233 °C decomp. ^1^H NMR (600 MHz, DMSO-*d*_6_) δ ppm: 13.28 (s, 1H, SH), 8.73 (t, 1H, NH, *J* = 12 Hz), 7.19–7.13 (m, 1H, ArH), 6.88–6.84 (m, 2H, ArH), 6.76–6.73 (m, 1H, ArH), 6.48 (s, 1H, CH_pyridine_), 5.05 (s, 2H, CH_2_), 4.51 (d, 2H, CH_2_, *J* = 6 Hz), 3.60 (s, 3H, CH_3_), 3.13 (m, 4H, CH_2-piperazine_), 2.77 (m, 4H, CH_2-piperazine_), 2.25 (s, 3H, CH_3_), 2.05 (s, 3H, CH_3_). ^13^C NMR (151 MHz, DMSO-*d*_6_) δ ppm: 174.28, 168.60, 167.14, 152.74, 150.26, 148.42, 146.55, 137.08, 134.23, 130.84, 118.57, 115.30, 114.34, 68.77, 50.11, 48.16, 34.37, 32.02, 19.29, 18.70. FT-IR (selected lines, γ_max_, cm^−1^): 3194 (N-H), 2834 (C-H aliph.), 1656 (C=O). HR-MS (ESI-MS) (*m*/*z*): calcd. for C_23_H_28_ClN_7_OS_2_ [M + H]^+^: 518.1558; found: 518.1554.

**10** 4,6-dimethyl-*N*-[(4-methyl-5-thioxo-2-{[4-(4-nitrophenyl)piperazinyl]methyl}-4,5-dihydro-1*H*-1,2,4-triazol-3-yl)methyl]-2-sulfanylpyridine-3-carboxamide.

Yellow solid, yield: 88%, m.p. 250–253 °C decomp. ^1^H NMR (600 MHz, DMSO-*d*_6_) δ ppm: 13.30 (s, 1H, SH), 8.73 (t, 1H, NH, *J* = 12 Hz), 8.02 (d, 2H, ArH, *J* = 6 Hz), 7.00 (d, 2H, ArH, *J* = 6 Hz), 6.47 (s, 1H, CH_pyridine_), 5.09 (s, 2H, CH_2_), 4.51 (d, 2H, CH_2_, *J* = 6 Hz), 3.61 (s, 3H, CH_3_), 3.46 (m, 4H, CH_2-piperazine_), 2.79 (m, 4H, CH_2-piperazine_), 2.27 (s, 3H, CH_3_), 2.04 (s, 3H, CH_3_). ^13^C NMR (151 MHz, DMSO-*d*_6_) δ ppm: 174.27, 168.61, 167.11, 155.04, 149.14, 148.44, 146.58, 137.22, 137.01, 126.19, 115.26, 113.07, 68.66, 49.97, 46.76, 34.25, 32.03, 19.51, 18.66. FT-IR (selected lines, γ_max_, cm^−1^): 3260 (N-H), 2847 (C-H aliph.), 1655 (C=O). HR-MS (ESI-MS) (*m*/*z*): calcd. for C_23_H_28_N_8_O_3_S_2_ [M + H]+: 529.1799; found: 529.1815.

**11** 4,6-dimethyl-*N*-[(4-methyl-5-thioxo-2-{[4-(4-methylphenyl)piperazinyl]methyl}-4,5-dihydro-1*H*-1,2,4-triazol-3-yl)methyl]-2-sulfanylpyridine-3-carboxamide.

White solid, yield: 94%, m.p. 246–249 °C decomp. ^1^H NMR (600 MHz, DMSO-*d*_6_) δ ppm: 13.31 (s, 1H, SH), 8.77 (t, 1H, NH, *J* = 12 Hz), 7.00 (d, 2H, ArH, *J* = 6 Hz) 6.80 (d, 2H, ArH, *J* = 6 Hz), 6.50 (s, 1H, CH_pyridine_), 5.06 (s, 2H, CH_2_), 4.53 (d, 2H, CH_2_, *J* = 6 Hz), 3.63 (s, 3H, CH_3_), 3.04 (m, 4H, CH_2-piperazine_), 2.80 (m, 4H, CH_2-piperazine_), 2.27 (s, 3H, CH_3_), 2.18 (s, 3H, CH_3_), 2.09 (s, 3H, CH_3_). ^13^C NMR (151 MHz, DMSO-*d*_6_) δ ppm: 174.30, 168.60, 167.14, 149.46, 149.05, 148.41, 146.61, 137.06, 129.79, 128.21, 116.34, 115.30, 68.83, 50.29, 49.25, 34.25, 32.01, 20.49, 19.57, 18.69. FT-IR (selected lines, γ_max_, cm^−1^): 3199 (N-H), 2830 (C-H aliph.), 1660 (C=O). HR-MS (ESI-MS) (*m*/*z*): calcd. for C_24_H_31_N_7_OS_2_ [M + H]^+^: 498.2104; found: 498.2126.

**12** 4,6-dimethyl-*N*-[(4-(propan-2-yl)-5-thioxo-2-{[4-(3-chlorophenyl)piperazinyl]-methyl}-4,5-dihydro-1*H*-1,2,4-triazol-3-yl)methyl]-2-sulfanylpyridine-3-carboxamide.

White solid, yield: 75%, m.p. 211–214 °C decomp. ^1^H NMR (600 MHz, DMSO-*d*_6_) δ ppm: 13.27 (s, 1H, SH), 8.77 (t, 1H, NH, *J* = 12 Hz), 7.20–7.17 (m, 1H, ArH), 6.91–6.87 (m, 2H, ArH), 6.77–6.76 (m, 1H, ArH), 6.49 (s, 1H, CH_pyridine_), 5.06 (s, 2H, CH_2_), 4.90 (h, 1H, CH, *J = 30 Hz*), 4.55 (d, 2H, CH_2_, *J* = 6 Hz), 3.14 (m, 4H, CH_2-piperazine_), 2.79 (m, 4H, CH_2-piperazine_), 2.28 (s, 3H, CH_3_), 2.13 (s, 3H, CH_3_), 1.58 (d, 6H, 2xCH_3_, *J* = 12 Hz). ^13^C NMR (151 MHz, DMSO-*d*_6_) δ ppm: 174.50, 171.65, 167.28, 152.73, 148.61, 148.34, 146.64, 136.99, 134.23, 130.87, 118.55, 114.97, 114.35, 68.38, 50.17, 49.77, 48.16, 19.85, 19.57, 18.70. FT-IR (selected lines, γ_max_, cm^−1^): 3299 (N-H), 2839 (C-H aliph.), 1659 (C=O). HR-MS (ESI-MS) (*m*/*z*): calcd. for C_25_H_32_ClN_7_OS_2_ [M + H]^+^: 546.1871; found: 546.1882.

**13** 4,6-dimethyl-*N*-[(4-(propan-2-yl)-5-thioxo-2-{[4-(4-nitrophenyl)piperazinyl]-methyl}-4,5-dihydro-1*H*-1,2,4-triazol-3-yl)methyl]-2-sulfanylpyridine-3-carboxamide.

Yellow solid, yield: 65%, m.p. 216–217 °C decomp. ^1^H NMR (600 MHz, DMSO-*d*_6_) δ ppm: 13.28 (s, 1H, SH), 8.74 (t, 1H, NH, *J* = 12 Hz), 8.02 (d, 2H, ArH, *J* = 12 Hz), 7.00 (d, 2H, ArH, *J* = 12 Hz), 6.47 (s, 1H, CH_pyridine_), 5.08 (s, 2H, CH_2_), 4.88 (h, 1H, CH, *J* = 12 Hz), 4.53 (d, 2H, CH_2_, *J* = 6 Hz), 3.45 (m, 4H, CH_2-piperazine_), 2.79 (m, 4H, CH_2-piperazine_), 2.27 (s, 3H, CH_3_), 2.10 (s, 3H, CH_3_), 1.57 (d, 6H, 2xCH_3_, *J* = 12 Hz). ^13^C NMR (151 MHz, DMSO-*d*_6_) δ ppm: 174.48, 171.65, 167.22, 155.08, 148.65, 148.34, 146.99, 137.29, 136.97, 126.13, 115.21, 113.06, 68.24, 50.02, 49.80, 46.69, 19.81, 19.57, 18.69. FT-IR (selected lines, γ_max_, cm^−1^): 3437 (N-H), 2848 (C-H aliph.), 1672 (C=O). HR-MS (ESI-MS) (*m*/*z*): calcd. for C_25_H_32_N_8_O_3_S_2_ [M + H]^+^: 557.2112; found: 557.2129.

### 3.2. Biological Assays

#### 3.2.1. Cell Lines and Conditions

The biological activity of the newly synthesized compounds was assessed in the context of anticancer activity. For this purpose, several gastrointestinal cancer lines were selected: gastric cancer (EPG 257, which belongs to the collection of the Laboratory of Cell Culture and Advanced Therapy at the University of Environmental and Life Sciences in Wrocław), and four colorectal cancer cell lines (LoVo, Caco-2, and HT-29) were obtained from the European Collection of Authenticated Cell Cultures (ECACC) and were delivered by Sigma-Aldrich, a certified distributor for the biobank. Additionally, LoVo cells were used to develop a doxorubicin-resistant subline, referred to as LoVo/Dx. Resistance was induced through gradual adaptation to increasing concentrations of doxorubicin, starting from 0.1 µg/mL and incrementally raised to 0.8 µg/mL. After reaching the maximum concentration, the cells were maintained in a medium containing 0.4 µg/mL doxorubicin for a period of three months to stabilize the resistant phenotype. This procedure allowed for the selection of cells capable of surviving prolonged exposure to the drug, mimicking clinically relevant drug resistance mechanisms. The resulting LoVo/Dx subline, resistant to doxorubicin, was cultured in DMEM/F-12 medium and utilized in assays evaluating the effectiveness of novel compounds against multidrug-resistant cancer cells. The LoVo/Dx line originated from the biobank and laboratory collection of Cell Culture and Advanced Therapy at the University of Environmental and Life Sciences in Wrocław, where the resistant phenotype was confirmed through functional assays such as reduced accumulation of rhodamine 123 and maintained viability in the presence of doxorubicin. The non-cancerous cell control was normal colonic epithelial cells (CCD 841 CoTr from American Type Culture Collection, (ATCC, Manassas, VA, USA)). Cell cultures were performed under standard conditions (5% CO_2_, 95% humidity, and 37 °C). Each cell line was cultured in the recommended medium: (1) HT-29 in McCoy’s 5a, (2) Caco-2 in Minimal Essential Medium (MEM, Biological Industries, Beit Haemek, Israel), (3) CCD 841 CoTr in Dulbecco’s Modified Eagle Medium (DMEM, Biological Industries, Beit Haemek, Israel), (4) LoVo in Dulbecco’s Modified Eagle Medium/F-12 (DMEM/F-12; Biological Industries, Beit Haemek, Israel), (5) LoVo/Dx in DMEM/F-12, and (6) EPG in DMEM. Culture media were supplemented with 10% fetal bovine serum (FBS, Sigma-Aldrich, Darmstadt, Germany) and antibiotics (penicillin and streptomycin; Sigma-Aldrich, Darmstadt, Germany). For assays, trypsinized (TrypLE solution, Thermo Fisher Scientific, Waltham, MA, USA) cells were seeded at appropriate densities of 10,000 cells per well for MTT and determination of apoptotic and necrotic cell numbers; 30,000 cells per well for the rhodamine accumulation assay; and 500,000 cells per well for ELISA tests.

#### 3.2.2. Tested Compounds

The tested compounds and 5-fluorouracil (5-FU, Sigma-Aldrich, Darmstadt, Germany) were dissolved to a concentration of 10 mM in dimethyl sulfoxide (DMSO, Sigma-Aldrich, Darmstadt, Germany). Stocks of reference drugs were also prepared to a concentration of 10 mM: verapamil (Sigma-Aldrich, Darmstadt, Germany) in methanol (Sigma-Aldrich, Darmstadt, Germany), doxorubicin in distilled water, and cisplatin (Sigma-Aldrich, Darmstadt, Germany) in physiological saline (Biological Industries, Beit Haemek, Israel). Stocks were stored at −80 °C until use, but for no longer than 3 months. Before performing biological tests, they were prepared at concentrations ranging from 1 to 500 μM in the media recommended for the cell line.

#### 3.2.3. Viability Assay

The cytotoxicity of the compounds and drugs used as controls was assessed using the MTT assay. The assay also included a DMSO control. After seeding and allowing the cells to adhere overnight, they were treated with the tested compounds and drugs for another 24 h. After this time, the supernatant was removed, and a 1 mg/mL MTT solution was added for 2 h in 5% CO_2_, 95% humidity, at 37 °C. The supernatant was then removed, and isopropanol was added to dissolve the purple crystals over 30 min by shaking. Absorbance was measured at 570 nm in a Multiskan GO microplate reader (Thermo Fisher Scientific; Waltham, MA, USA). All reagents used for the assays were purchased from Sigma-Aldrich, Darmstadt, Germany.

#### 3.2.4. Rh-123 Assay

Using the HT-29, LoVo, and LoVo/Dx cell lines, P-glycoprotein activity was assessed in the Rh-123 assay for compounds **5** and **6**, which had the best CC_50_ values. The activity was measured for cells treated with a 50 µM concentration of the compounds and drugs in the recommended media for each cell line (without FBS) in a volume of 100 µL for 24 h. In this test, verapamil served as the control. After the incubation period, a 10 mM Rhodamine-123 (Rh-123) solution was prepared in a 1:1 DMSO mixture. Rh-123 was added to the wells to a final concentration of 12.5 µM and incubated for 60 min. The cells were then lysed with 150 µL/well of 20 mM Tris-HCl, pH 7.7, containing 0.2% sodium dodecyl sulfate (SDS). Fluorescence was measured at 485 nm excitation and 538 nm emission using a Biotek Synergy H1 microplate reader (Agilent Technologies, Inc., Santa Clara, CA, USA). All reagents used for the assays were purchased from Sigma-Aldrich, Darmstadt, Germany.

#### 3.2.5. Detection of Apoptosis

The assessment of cell death—apoptosis was performed after 24 h of incubation of HT-29 cells with a concentration of 1 µM of compounds 5 and 6 and doxorubicin. Fluorescein-conjugated annexin V in PBS with Mg^2+^ and Ca^2+^ ions (Thermo Fisher Scientific, Waltham, MA, USA) was then used. To ensure that all cells (both live and dead) were counted, 10 µM Hoechst (Sigma-Aldrich, Darmstadt, Germany) was added to stain cell nuclei. The culture was then incubated for 20 min at 37 °C. Apoptosis analysis was performed under a fluorescence microscope (EVOS FL, Thermo Fisher Scientific, Waltham, MA, USA), using 10× magnification, in triplicate for each compound. The open-source ImageJ platform with the Fiji plugin [57] was used to analyze the number of all cells, as well as those in the apoptotic and necrotic phases.

The fluorescence signal was interpreted as follows: green fluorescence indicated apoptotic cells (annexin V staining), red fluorescence corresponded to necrotic cells, and blue fluorescence (Hoechst staining) marked all cell nuclei.

#### 3.2.6. Preparation of Cell Lysates for Human Caspase-3 (Active) ELISA Kit (KHO1091) and p53 Human ELISA Kit (BMS256)

To assess the level of caspase-3 and p53 protein, HT-29 cell lysates were used after 24 h of incubation with compounds **5** and **6**. The supernatant was collected in the prepared and labeled tubes, the cells from the wells were scraped, and the wells were washed with cold PBS, which was also collected. The tubes were then centrifuged, the supernatant was removed, and the cell pellet was lysed in a cell extraction vessel for 30 min on ice, with centrifugation every 10 min. The lysate was transferred to centrifuge tubes and centrifuged at 13,000 rpm for 10 min at 4 °C. The supernatant was transferred to clean tubes in a microcentrifuge.

The following kits were used: Human Caspase-3 (active) ELISA Kit (KHO1091) and p53 Human ELISA Kit (BMS256), used following the manufacturer’s instructions. ELISA kits were purchased from Thermo Fisher Scientific (Waltham, MA, USA). The absorbance was measured using a Multiskan GO microplate reader (Thermo Fisher Scientific, Waltham, MA, USA) at wavelengths specific to each kit, as recommended by the manufacturer.

#### 3.2.7. Cytokine Levels (IL-1β and IL-6)

Cytokine levels (IL-1β and IL-6) in the supernatant were measured for HT-29 and EPG cells treated with compounds **5** and **6**, according to the instructions provided by the manufacturer of the ELISA kits were purchased from Abcam (Cambridge, UK). The absorbance was measured using a Multiskan GO microplate reader (Thermo Fisher Scientific, Waltham, MA, USA) at wavelengths specific to each kit, as recommended by the manufacturer.

#### 3.2.8. Statistical Analysis

The statistical evaluation was conducted using Statistica v13.3 [58] and GraphPad PRISM [59] software. All graphs were prepared in Excel. Data were normally distributed and had equal variance, allowing for the use of one-way ANOVA with post hoc Tukey’s test. Significance was set at *p* < 0.05.

Results are expressed as the mean ± SD (standard deviation) relative to the respective control (E/E_0_), where E represents the mean result for the measured sample, and E_0_ represents the mean result for the control. The control consisted of cell culture incubated solely with the appropriate medium, without the addition of tested compounds or control drugs.

Biological assays were performed in three independent replicates, each involving four samples. ELISA determinations were repeated three times. For cytotoxicity assessment, mathematical models were developed using the Statistica v13.3 program, from which the CC_50_ was calculated. The CC_50_ represents the concentration that inhibits 50% of cell viability. Additionally, the selectivity index (SI) was calculated as the ratio of the CC_50_ value determined for healthy cells (CCD 841 CoTr) to the CC_50_ value determined for cancer cells. The SI provides a measure of the compound’s selectivity toward cancer cells over non-cancerous cells, with higher values indicating greater selectivity.

### 3.3. Computational Methodology

The models of the synthesized *N*-Mannich bases were constructed using the Avogadro [60] and SAMSON 2022 R2 [61] programs. The geometry optimization was performed based on Density Functional Theory (DFT) [29,30] using ωB97-XD exchange-correlation functional [62] in conjunction with def2-TZVP basis set [63]. Subsequently, harmonic frequencies were computed to confirm that the obtained structures correspond with the minima on the Potential Energy Surface (PES). The simulations were carried out in vacuo and with a solvent reaction field to reproduce the polar environment influence on the metric and electronic structure parameters. The IEF-PCM model was applied for this purpose [64,65], and water as a solvent. Next, the wavefunctions for further Quantum Theory of Atoms in Molecules (QTAIM) [66,67] were generated in both phases using the above-mentioned level of theory. The quantum-chemical simulations were performed using the Gaussian 16 Rev. C.0.1 suite of programs [68]. The QTAIM theory application enabled the detection of non-covalent interactions present in the compounds studied. The electron density at Bond Critical Points (BCPs), as well as its Laplacian, was calculated. In addition, the QTAIM atomic charges were computed to show electron density distribution changes upon the substitution. The QTAIM analysis was performed in both phases. The DFT results were visualized using the VMD 1.9.3 program. The QTAIM analysis was performed with the assistance of the AIMAll program [69].

In the next step, the molecular docking method was applied to detect the binding affinity and host-guest interactions [31]. The structures of the caspase-3 (PDB code: 1RE1) [70] and MDM-2 (PDB code: 3JZK) [71] enzymes with cocrystallized ligands (nicotinic acid aldehyde and chromenotriazolopyrimidine, respectively) investigated in the docking study were extracted from the Protein Data Bank (PDB) database [72,73]. The calculation of the binding pockets volume and their visualization was carried out using the PyVol 1.7.8, a PyMOL 3.0.0 plugin software [74] with minimum radius and maximum radius parameters for 1RE1 and 3JZK set to 1.2 Å and 5.0 Å. The minimum volume parameter was set to 200 Å for both. The electrostatic potential (ESP) maps were generated using the APBS server [75], and the PyMOL 3.0.0 [76] suite of programs. The calculations of molecular and van der Waals volumes were performed using a spherical probe with the assistance of the Molovol 1.1.1 [77] program. In the next step, the macromolecules and ligands were prepared for the docking studies according to the standard procedure. The structures of ligands and reference compounds optimized with the solvent reaction field were taken for further molecular docking experiments (the influence of the polar environment on the metric parameters was reproduced and incorporated). The AutoDockTools 1.5.7 suite of programs and scripts [78] was used to add polar hydrogens and assign partial charges to the proteins and ligands. The molecular docking studies were carried out using the AutoDockFR 1.2 software [79]. The protocol utilized in the study consisted of 150 independent runs and 3,500,000 energy evaluations. For caspase-3 (1RE1) protein, the computational box center was set to (37.076, 93.936, 19.202), whereas its dimensions were equal to 20.50, 16.00, 20.50, respectively (all values in Å). In the case of MDM-2 (3JZK) macromolecule, the box was centered at (9.948, −13.818, 24.733) and was of the size of 15.50, 14.75, 19.25 (the same up to 0.25 Å in both models of MDM-2 enzyme (the raw and the modeled)). To confirm that the prepared setup works properly, the redocking experiment was performed. It indicated the validity of the chosen protocol: RMSD values for caspase-3 (1RE1) and MDM-2 (3JZK) native ligands were equal to 0.83 and 0.72 Å, respectively [80]. For the MDM-2 protein, an additional modeling was performed concerning the missing fragments’ reproduction (therefore, in the manuscript we use the notation model 1 and model 2). The modeling of the missing loops region was performed without the use of the template, with the assistance of the ChimeraX interface to Modeller 10.6 [81,82]. As a result, ten different models of the MDM-2 enzyme were obtained (the largest RMSD value between two models in the obtained set was equal to 0.389 Å in the MDM-2 protein). The model chosen for further modeling was picked according to the smallest zDOPE metric value. The chosen ligand–protein poses were visualized using the ChimeraX 1.8 (Molecular graphics and analyses performed with UCSF ChimeraX, developed by the Resource for Biocomputing, Visualization, and Informatics at the University of California, San Francisco, with support from National Institutes of Health R01-GM129325 and the Office of Cyber Infrastructure and Computational Biology, National Institute of Allergy and Infectious Diseases) [83] and LigPlot+ [84] programs, whereas the protein binding pockets and their electrostatic potential were visualized using an open-source PyMOL 3.0.0 [76].

## 4. Conclusions

In the present study, ten new *N*-Mannich bases derived from 1,2,4-triazole were successfully synthesized and characterized by detailed spectral analyses. All compounds were screened for their anticancer activity. The compound **6**-bearing phenyl group at the *N*-4 position and the 4-methylphenyl piperazine moiety at the *N*-2 position of the 1,2,4-triazole scaffold was found as the most potent cytotoxic agent of the series with CC_50_ values of 57.70 ± 5.40 µM against EPG and 75.10 ± 5.10 µM against Caco-2, approximately two- to five-fold more effective than the standard drugs cisplatin and 5-FU. The low toxicity of compound **6** towards normal CCD 841 CoTr cells confirms its safety profile. Further evaluation showed the good ability of compound **6** to the inhibition of the efflux function of P-glycoprotein in P-gp-expressing cell lines (HT-29, LoVo, and LoVo/Dx). Moreover, *N*-Mannich base **6** was found to exert its considerable pro-apoptotic activity through a significant increase in the caspase-3 and p53 protein levels. An application of QTAIM theory qualitatively demonstrated the network of non-covalent interactions in the compounds studied. Molecular docking study revealed the binding affinity of all the studied compounds to MDM-2 protein, suggesting that the p53–MDM-2 pathway may be involved in compound **6**-induced apoptosis in HT-29 cancer cells. Thus, from the present study, it can be concluded that compound **6** could be considered a promising lead candidate for further anticancer studies.

## Figures and Tables

**Table 1:** Cytotoxic activity of tested compounds and reference drugs against the selected cell lines evaluated in the MTT assay and presented as the cytotoxic concentration (CC_50_ ± SD) that causes a 50% reduction in viable cells. SD is the standard deviation. SI is the selectivity index, which is the ratio of the cytotoxic concentration (CC_50_) against healthy cells (CCD 841 CoTr) to the cytotoxic concentration against cancer cells.

Comp.	CCD 841 CoTr	EPG		Caco-2		LoVo		LoVo/Dx		HT-29	
	CC_50_ ± SD ^a^ (µM)	CC_50_ ± SD ^a^ (µM)	SI	CC_50_ ± SD ^a^ (µM)	SI	CC_50_ ± SD ^a^ (µM)	SI	CC_50_ ± SD ^a^ (µM)	SI	CC_50_ ± SD ^a^ (µM)	SI
**4**	236.20 ± 3.50	140.70 ± 2.50	1.68	132.90 ± 2.70	1.78	200.60 ± 4.60	1.18	201.20 ± 4.10	1.17	187.60 ± 1.60	1.26
**5**	220.80 ± 2.40	83.00 ± 4.60	2.66	227.60 ± 5.20	0.97	Non-toxic ^b^	>>1 ^c^	183.50 ± 2.70	1.20	102.50 ± 2.70	2.15
**6**	387.80 ± 6.50	57.70 ± 5.40	6.72	75.10 ± 5.10	5.16	163.40 ± 4.60	2.37	195.20 ± 5.70	1.99	122.80 ± 3.90	3.16
**7**	486.80 ± 4.60	118.70 ± 4.50	4.10	291.80 ± 6.10	1.67	303.20 ± 7.10	1.61	128.90 ± 5.20	3.78	100.50 ± 4.20	4.84
**8**	Non-toxic ^b^	142.00 ± 6.30	>>1 ^c^	84.30 ± 6.80	>>1 ^c^	177.60 ± 5.30	>>1 ^c^	320.60 ± 6.80	>>1 ^c^	306.10 ± 4.90	>>1 ^c^
**9**	Non-toxic ^b^	231.20 ± 7.10	>>1 ^c^	88.60 ± 7.90	>>1 ^c^	152.50 ± 2.70	>>1 ^c^	260.00 ± 4.10	>>1 ^c^	253.10 ± 5.70	>>1 ^c^
**10**	Non-toxic ^b^	212.40 ± 3.70	>>1 ^c^	87.20 ± 5.20	>>1 ^c^	147.90 ± 3.70	>>1 ^c^	210.20 ± 3.30	>>1 ^c^	175.80 ± 6.40	>>1 ^c^
**11**	270.90 ± 7.10	118.90 ± 3.40	2.28	57.70 ± 4.80	4.69	136.90 ± 5.70	1.98	254.80 ± 4.80	1.06	242.30 ± 4.10	1.12
**12**	342.50 ± 3.40	262.80 ± 5.70	1.30	246.70 ± 4.90	1.39	445.90 ± 2.30	0.77	487.00 ± 5.90	0.70	471.20 ± 3.70	0.73
**13**	436.10 ± 2.70	120.20 ± 8.50	3.63	146.30 ± 3.60	2.98	193.00 ± 3.70	2.26	419.00 ± 6.20	1.04	392.6 ± 7.40	1.11
**Cis-platin**	14.50 ± 2.90	261.50 ± 4.60	0.06	136.40 ± 5.00	0.11	27.40 ± 2.70	0.53	47.80 ± 6.10	0.30	52.80 ± 7.10	0.27
**5-FU**	61.62 ± 3.70	111.20 ± 3.50	0.55	169.60 ± 3.70	0.36	72.20 ± 1.80	0.85	225.70 ± 2.70	0.27	428.50 ± 2.70	0.14

^a^ CC_50_ values were estimated from a 4-parameter logistic model with a Hill slope. Six concentrations of the tested solutions were used (i.e., 1, 5, 10, 50, 150, and 500 μM); ^b^ No toxicity was observed in the tested concentration range of 1–500 μM; ^c^ >> 1 means a very high value due to the lack of cytotoxic effect of the test compound on healthy CCD 841 CoTr cells.

**Table 2:** The effects of compound **6** and doxorubicin on the levels of p53 and caspase-3 proteins in the HT-29 cell line.

Compounds [1 µM]	p53		Caspase-3	
	Conc. ± SD [U/mL]	Fold Change	Conc. ± SD [ng/mL]	Fold Change
**Control**	1.80 ± 0.06	1	0.03 ± 0.08	1
**6**	3.50 ± 0.07	1.94	0.48 ± 0.07	16.00
**Doxorubicin**	7.62 ± 0.17	4.23	0.92 ± 0.23	30.67

**Table 3:** Effects of compound **6** on the level of interleukin-1β and interleukin-6 in the HT-29 cell line.

Compounds [1 µM]	IL-1β Conc. ± SD [pg/mL]	IL-6 Conc. ± SD [pg/mL]
**Control**	0.36 ± 0.08	9.30 ± 0.20
**6**	0.51 ± 0.11	7.80 ± 0.30

## Data Availability

The original contributions presented in this study are included in the article. Further inquiries can be directed to the corresponding authors.

## Supplementary Materials

The following supporting information can be downloaded at: https://www.mdpi.com/article/10.3390/ijms26146572/s1.

## Abbreviations

The following abbreviations are used in this manuscript:

5-FU	5-fluorouracil
A2780	Ovarian cancer cell line
A375	Melanoma cell line
A549	Lung adenocarcinoma cell line
ABC	ATP-binding cassette
ANOVA	Analysis of variance
ATCC	American Type Culture Collection
BA	Binding affinity
Bax	Bcl-2-associated X-protein
BCL-2	B-cell leukemia/lymphoma-2
BCP	Bond critical point
BS	Binding sites
BxPC-3	Pancreatic cancer cell line
C32	Amelanotic melanoma cell line
Ca9-22	Gingival squamous carcinoma cell line
Caco-2	Colorectal adenocarcinoma cell line
CC50	The half maximal cytotoxic concentration
CCD841CoTr	Normal colon epithelial cell line
DFT	Density functional theory
DMEM	Dulbecco’s modified Eagle medium
DMEM/F-12	Dulbecco’s modified Eagle medium/nutrient mixture F-12
DMSO	Dimethyl sulfoxide
DNA	Deoxyribonucleic acid
ECACC	European Collection of Authenticated Cell Cultures
ELISA	Enzyme-linked immunosorbent assay
EPG	Gastric adenocarcinoma cell lines
ESI-MS	Electrospray ionization mass spectrometry
ESP	Electrostatic potential
FBS	Fetal bovine serum
FT-IR	Fourier-transform infrared spectroscopy
HB	Hydrogen bond
hCA	Carbonic anhydrase
HepG2	Liver cancer cell line
HL-60	Leukemia cell line
HR-MS	High-resolution mass spectrometry
HT-29	Primary colorectal adenocarcinoma cell line
IARC	International Agency for Research on Cancer
IC50	The half maximal inhibitory concentration
IEF-PCM	Integral equation formalism–polarizable continuum model
IL-6	Interleukin 6
IL-1β	Interleukin 1β
ISO	International Organization of Standardization
K562	Chronic myelogenous leukemia (CML) cell line
LoVo	Colorectal adenocarcinoma cell line
LoVo/Dx	Doxorubicin-resistant colorectal adenocarcinoma cell line
MEM	Minimal essential medium
MCF-7	Breast cancer cell line
MCF-7/Dx	Doxorubicin-resistant breast cancer cell line
MCR	Multicomponent reaction
MDM-2	Murine double minute 2
MDR-1	Multidrug-resistant protein 1
MS	Mass spectrometry
MTT	3-(4,5-Dimethylthiazol-2-yl)-2,5-diphenyltetrazolium bromide
MV	Molecular volume
NMR	Nuclear magnetic resonance
NUGC	Gastric cancer cell line
OVCAR-3	Ovarian cancer cell line
PBS	Phosphate-buffered saline
PC-3	Pancreatic cancer cell line
PES	Potential energy surface
P-gp	P-glycoprotein
QTAIM	Quantum Theory of Atoms in Molecules
RCP	Ring critical points
Rh-123	Rhodamine-123
SAR	Structure–activity relationship
SD	Standard deviation
SDS	Sodium dodecyl sulfate
SI	Selectivity index
SNB-19	Glioma cell line
TLC	Thin-layer chromatography
TMS	Tetramethylsilane
TRAIL	Tumor necrosis factor-related apoptosis-inducing ligand
