# Supplementary material for: New 1,2,4-Triazole Derivatives with a N-Mannich Base Structure Based on a 4,6-Dimethylpyridine Scaffold as Anticancer Agents: Design, Synthesis, Biological Evaluation, and Molecular Modeling
Source: Int J Mol Sci. 2025 Jul 8;26(14):6572. doi: 10.3390/ijms26146572 (PMC12294558; doi:10.3390/ijms26146572)
Supplement: Supplementary file 1 [file ijms-26-06572-s001.zip › ijms-3722393-supplementary.pdf]

Supplementary Information

New 1,2,4-Triazole Derivatives with a *N*-Mannich Base Structure Based on a 4,6-Dimethylpyridine Scaffold as Anticancer Agents: Design, Synthesis, Biological Evaluation, and Molecular Modeling

Piotr Świątek [1], Teresa Glomb [1,*], Benita Wiatrak [2], Paulina Nowotarska [3], Tomasz Gębarowski [3], Kamil Wojtkowiak [4], Aneta Jezierska [4,*], and Małgorzata Strzelecka [1]

[1] Department of Medicinal Chemistry, Faculty of Pharmacy, Wroclaw Medical University, Borowska 211, 50-556 Wroclaw, Poland; piotr.swiatek@umw.edu.pl (P.Ś.), malgorzata.strzelecka@umw.edu.pl (M.S.)
[2] Department of Pharmacology, Wroclaw Medical University, J. Mikulicza-Radeckiego 2, 50-345 Wroclaw, Poland; benita.wiatrak@umw.edu.pl (B.W.)
[3] Department of Biostructure and Animal Physiology, Wroclaw University of Environmental and Life Sciences, Kożuchowska 1/3, 51-631 Wroclaw, Poland; paulina.nowotarska@upwr.edu.pl (P.N.); tomasz.gebarowski@upwr.edu.pl (T.G.)
[4] Faculty of Chemistry, University of Wrocław, ul. F. Joliot-Curie 14, 50-383 Wrocław, Poland; kamil.wojtkowiak2@uwr.edu.pl (K.W.)

* Correspondence: teresa.glomb@umw.edu.pl (T.G.); aneta.jezierska@uwr.edu.pl (A.J.)

Table of contents:

Table S1. Visualizations of Nuclear Magnetic Resonance (NMR) spectra of compounds **2-13** (DMSO-*d*6)………………………………………………………………………………. 3

Table S2. Visualizations of Fourier-Transform Infrared (FT-IR) spectra of compounds **2-13**…………………………………………………………………………………… 19

Figure S1. Optimized structures of the *N*-Mannich bases obtained as a result of DFT/ωB97XD/def2-TZVP simulations *in vacuo*……………………………………….. 24

Scheme S1. The core parts of the studied *N*-Mannich bases. Some hydrogen atoms are omitted for clarity. The atom numbering scheme was prepared especially for the study and QTAIM analyses……………………………………………………………………………….. 26

Table S3. Selected metric parameters (see Scheme S1) of five investigated compounds (denoted as **4**, **6**, **8**, **11** and **13**) obtained at the DFT/ωB97XD/Def2-TZVP level of theory *in vacuo* and using PCM solvation model…………………………………………………. 27

Table S4. Partial atomic charges for selected atoms (see Scheme S1) according to the QTAIM theory (the data is given for the gas phase results)………………………………………… 32

Table S5. Electron density and its Laplacian at selected Bond Critical Points (BCPs) derived from the QTAIM theory. For details, see Scheme S1. The data is given for gas phase results……………………………………………………………………………………. 33

Figure S2. QTAIM molecular graphs of the *N*-Mannich bases obtained as a result of DFT/ωB97XD/def2-TZVP simulations *in vacuo*………………………………………… 38

Figure S3. QTAIM molecular graphs of the *N*-Mannich bases obtained as a result of DFT/ωB97XD/def2-TZVP simulations with the PCM solvation model………………….. 43

Table S6. Binding affinity (kcal/mol) and molecular volume (Å) values from the molecular docking experiments for native ligands (YIN and NA3) as well as the synthesized set of *N*-Mannich bases……………………………………………………………………………… 48

XYZ coordinates for simulations *in vacuo* and with solvent reaction field (IEF-PCM) and water as a solvent…………………………………………………………………………… 49

Table S1. Visualizations of Nuclear Magnetic Resonance (NMR) spectra of compounds **2-13** (DMSO-*d*$_6$).

Comp.	^{1}H NMR, ^{13}C NMR spectra of 2-13
2a	

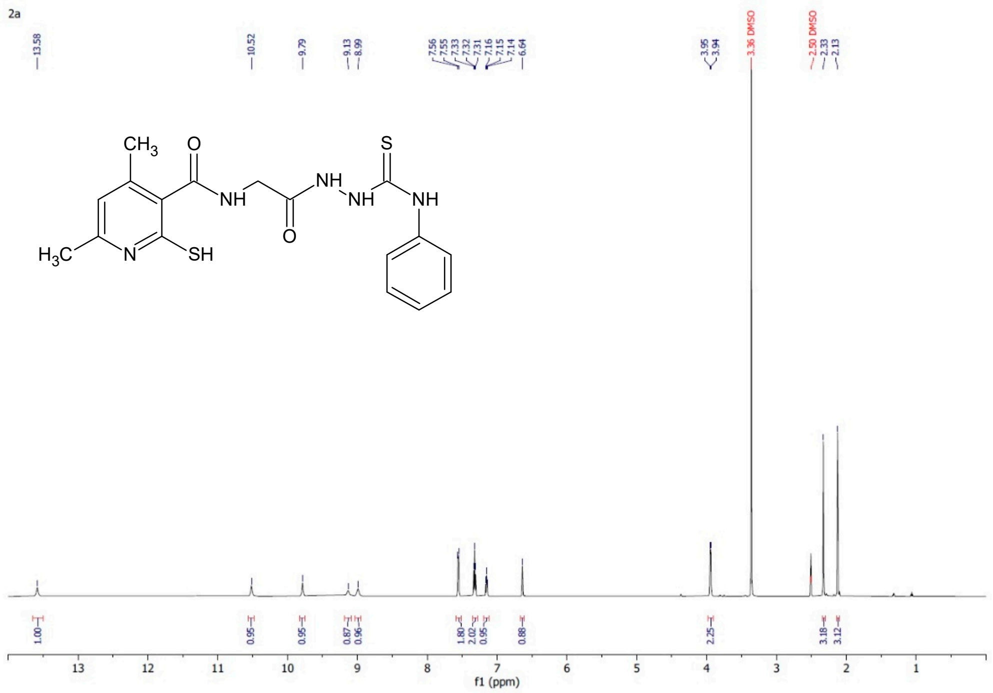

^{1}H NMR of 2a

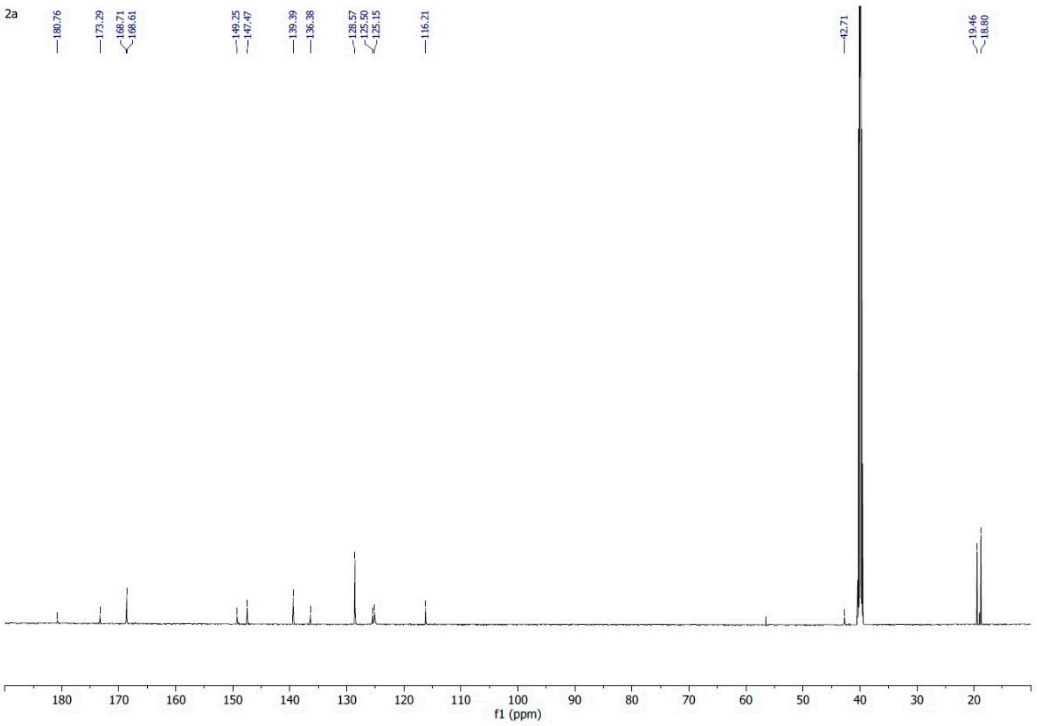

^{13}C NMR of 2a

2b

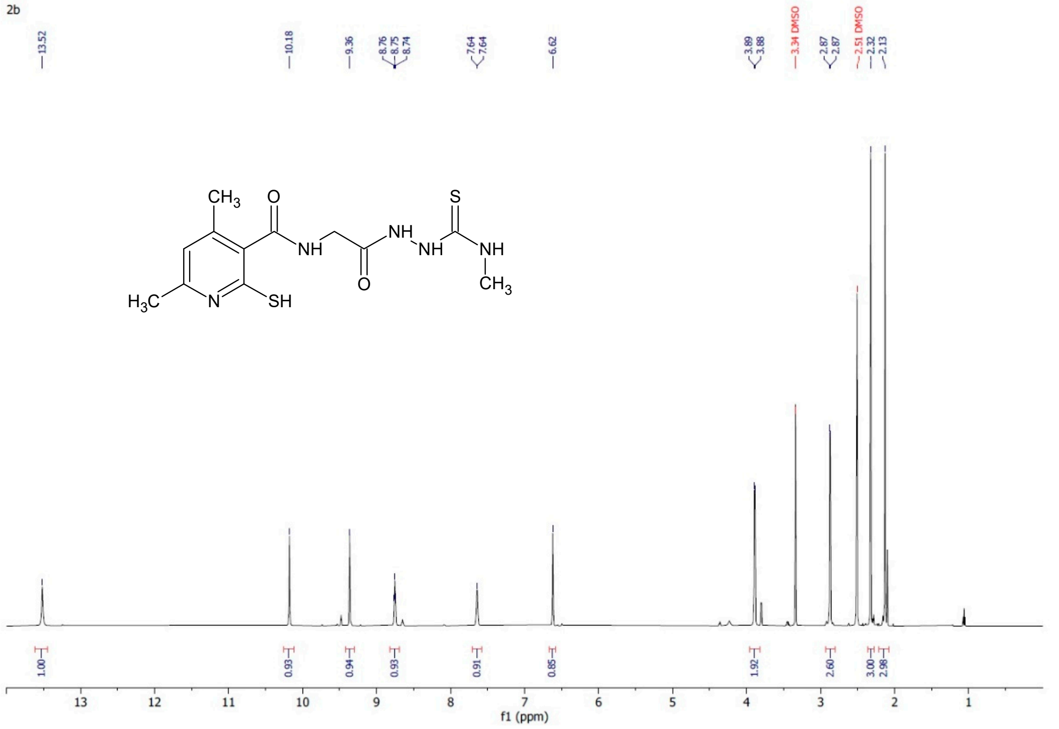

^{1}H NMR of 2b

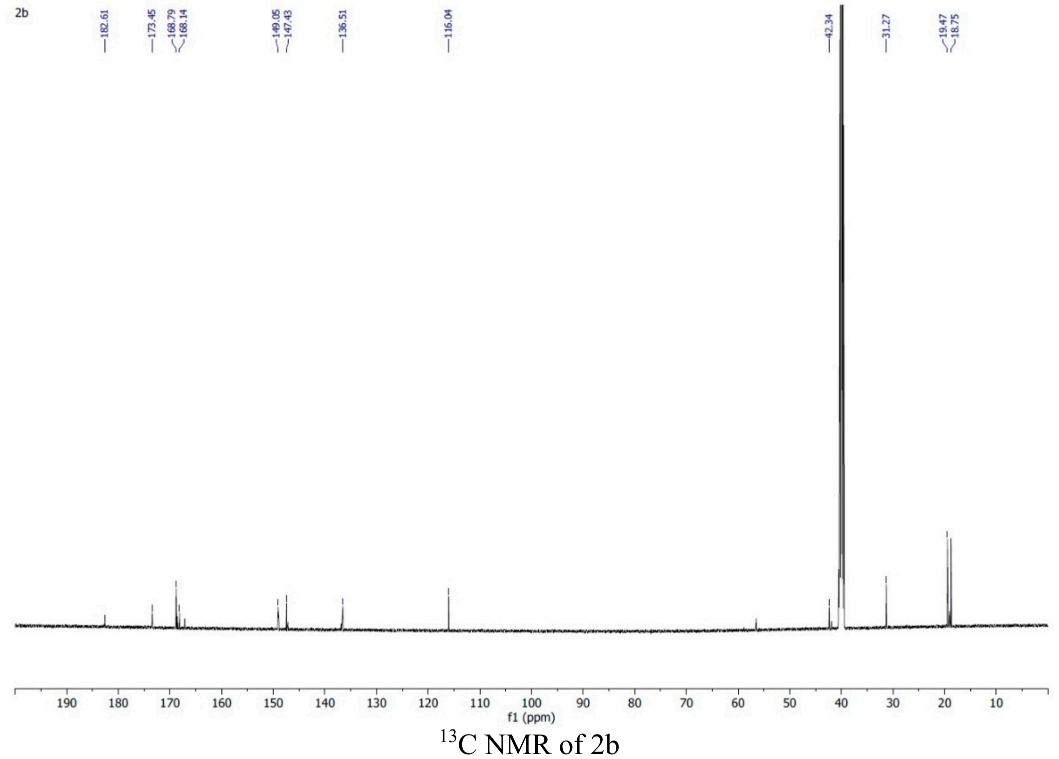

^{13}C NMR of 2b

2c

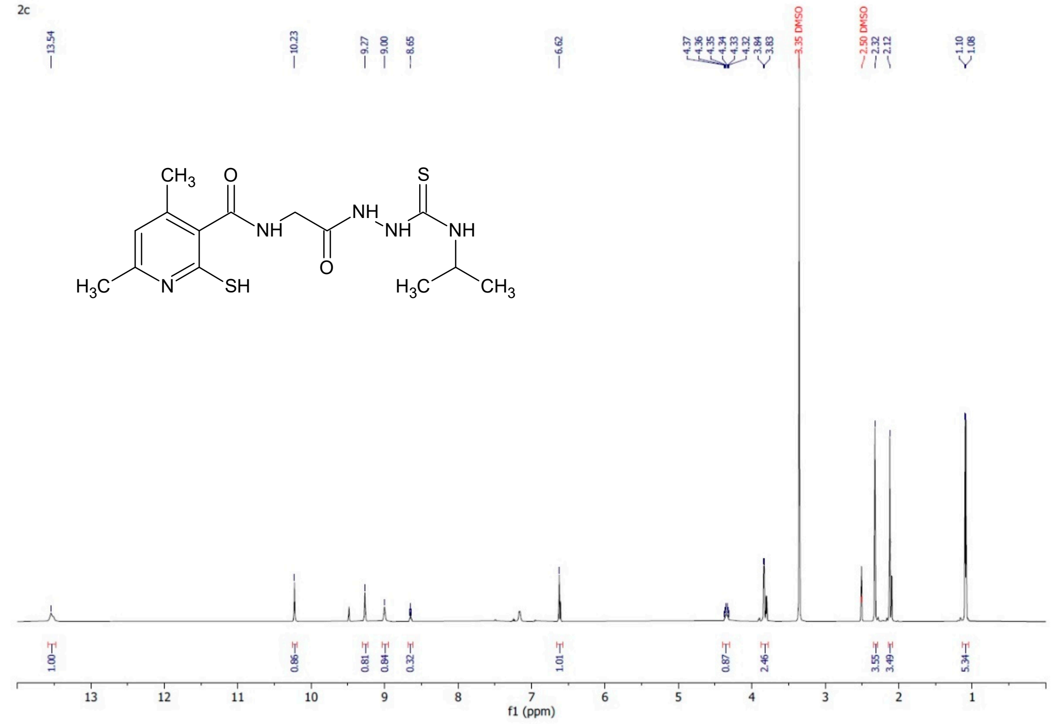

^{1}H NMR of 2c

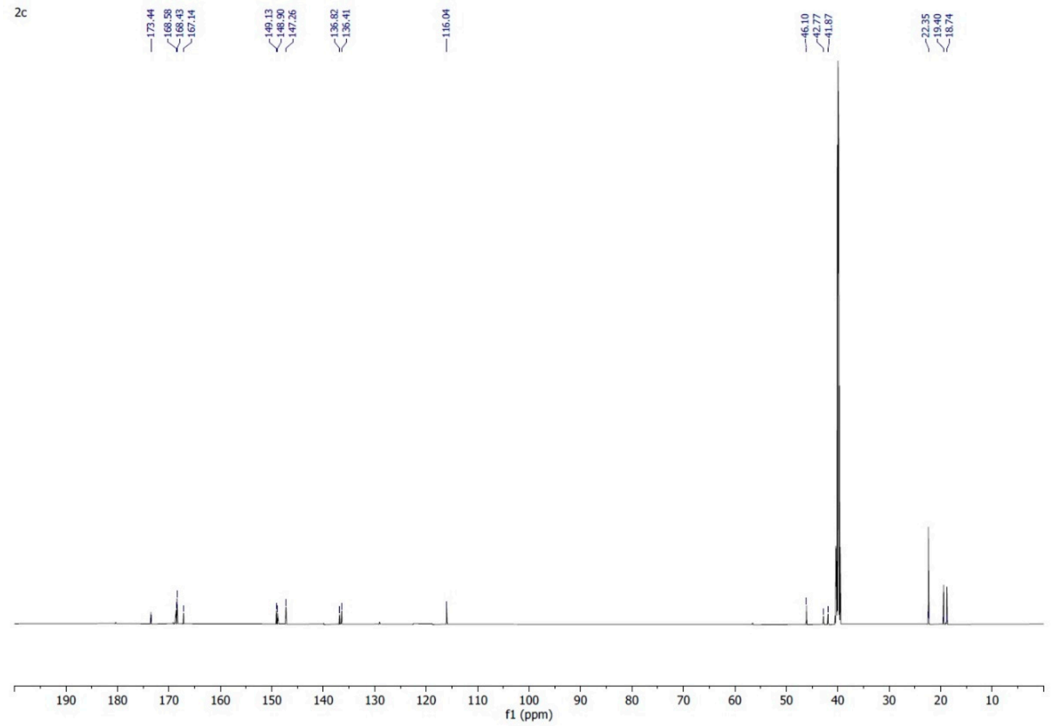

^{13}C NMR of 2c

3a

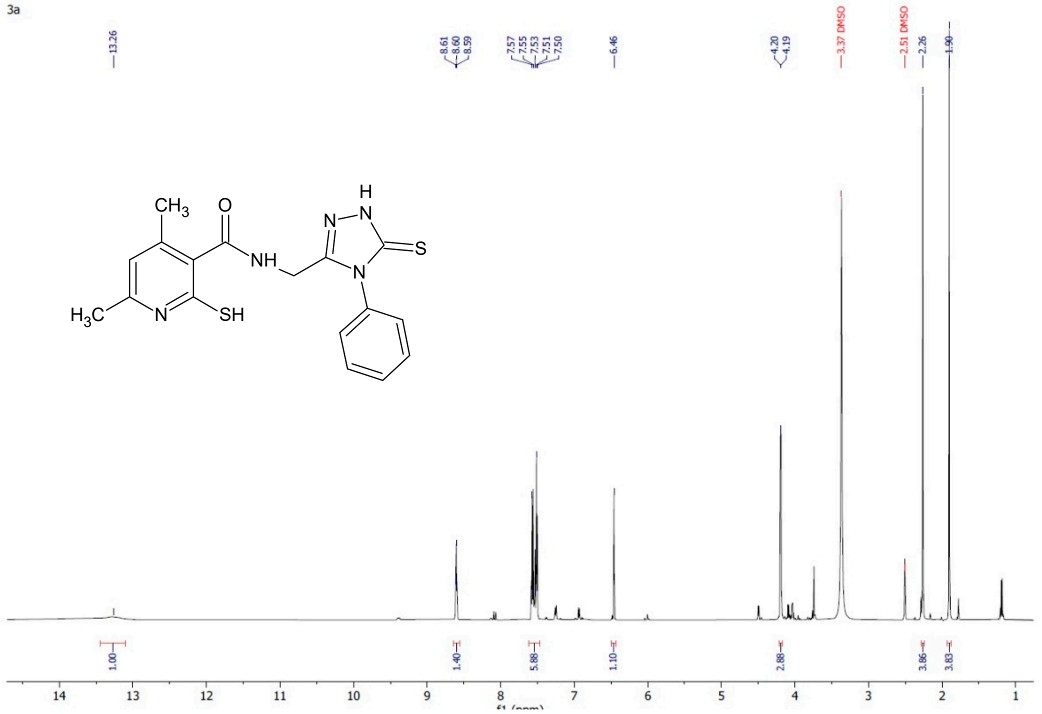

^{1}H NMR of 3a

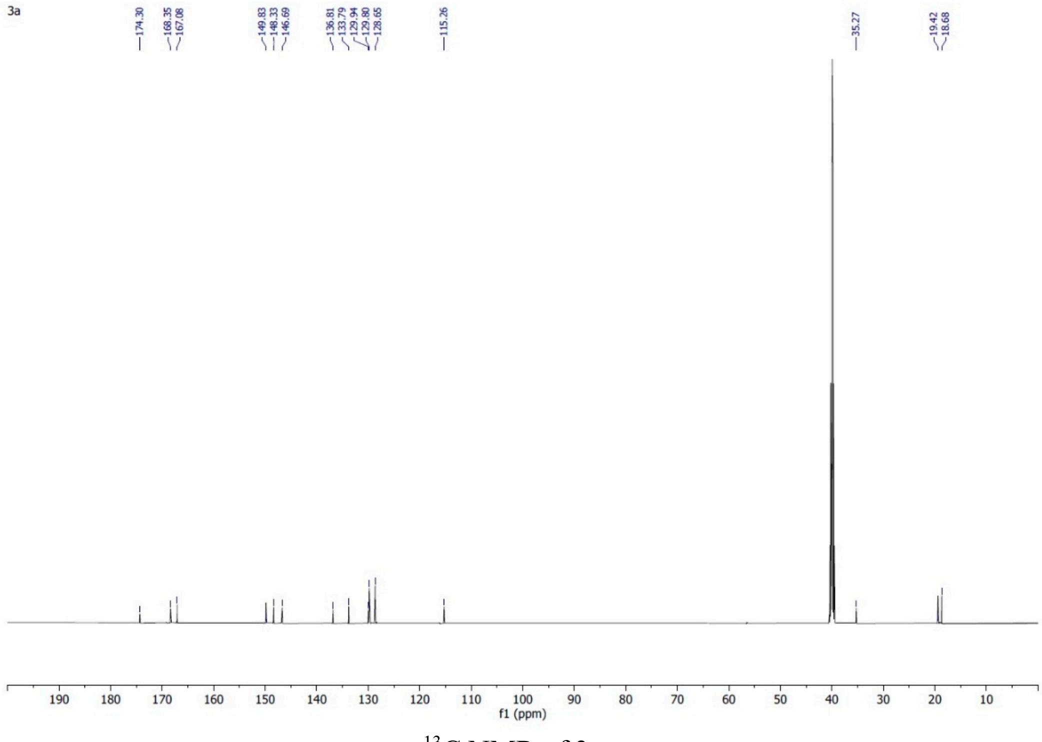

^{13}C NMR of 3a

3b	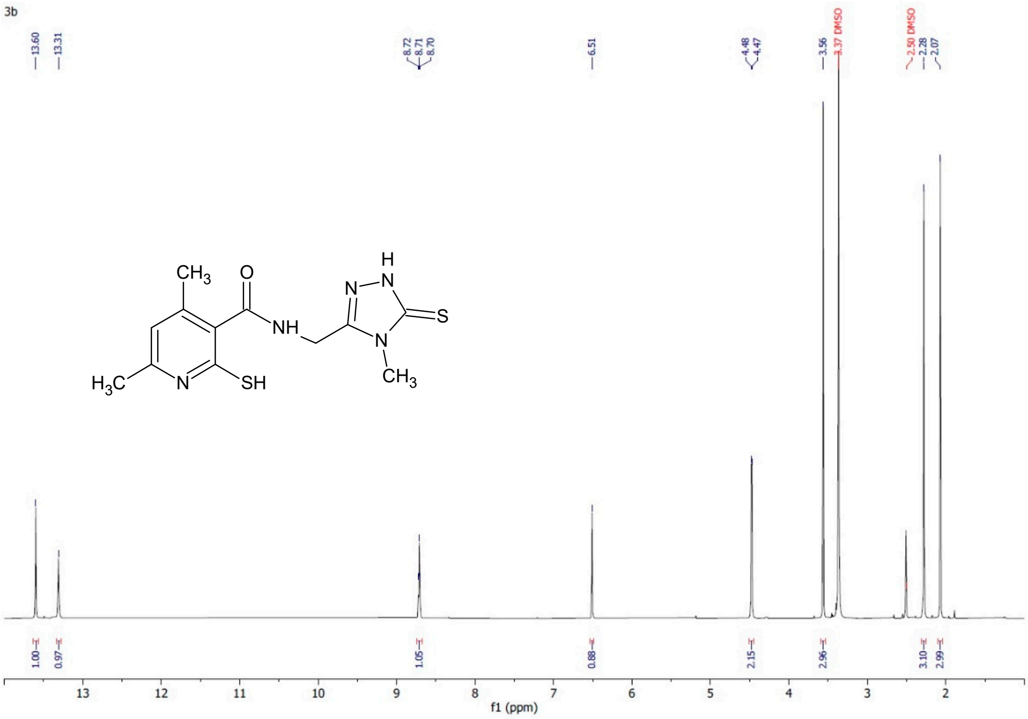 ^{1}H NMR of 3b ^{13}C NMR of 3b 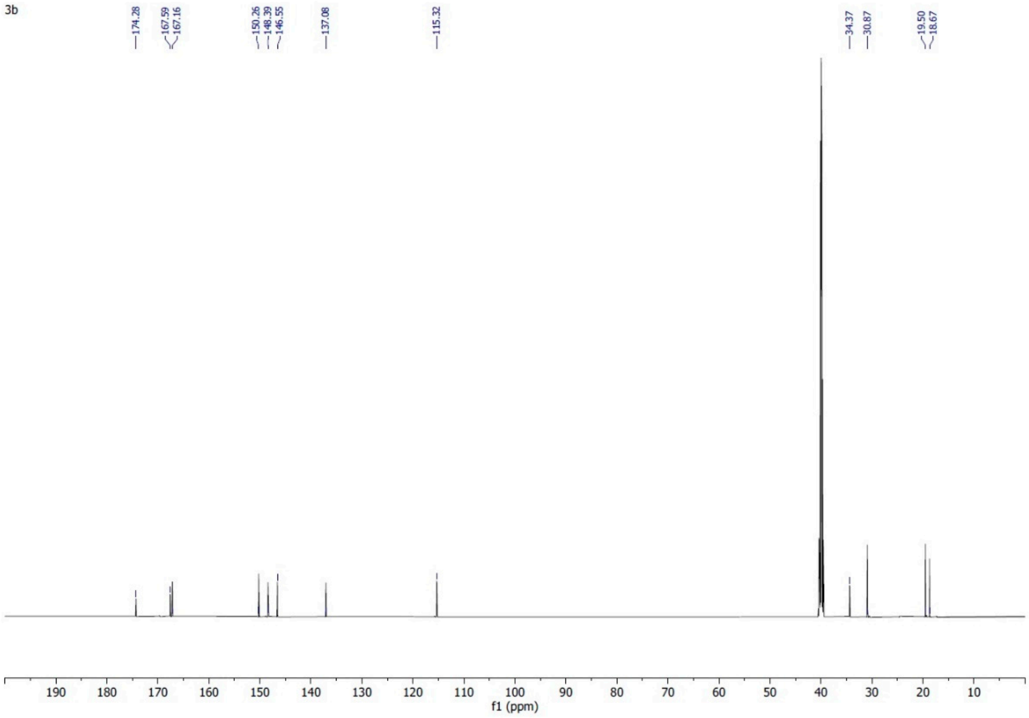

3c	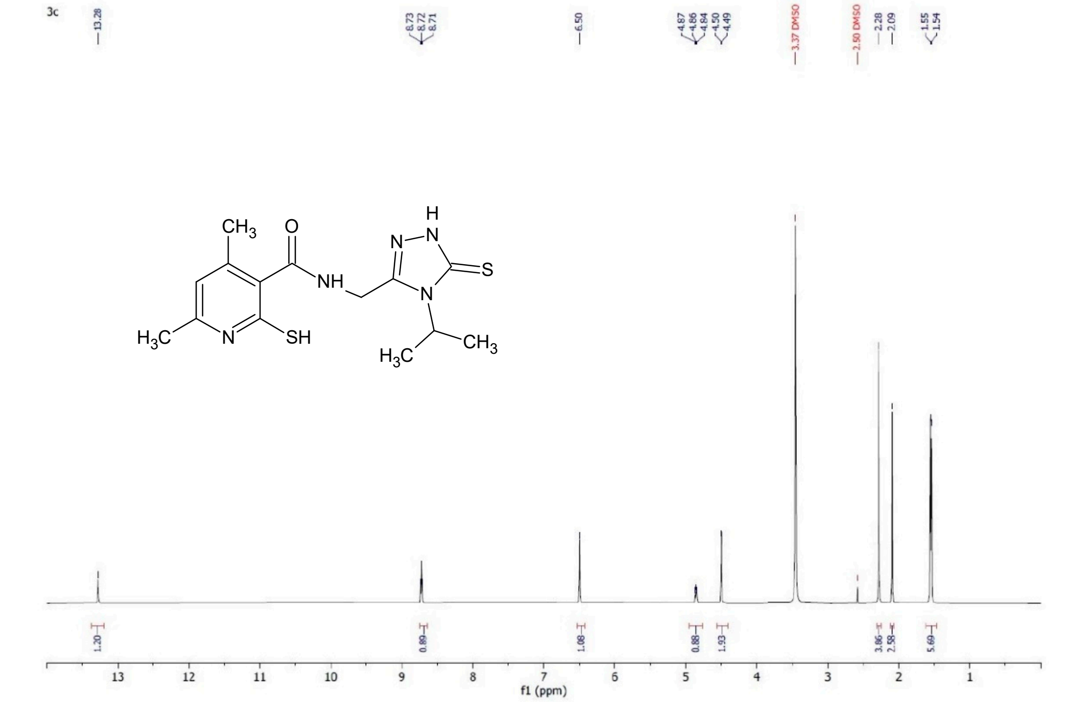<br><br>^{1}H NMR of 3c<br>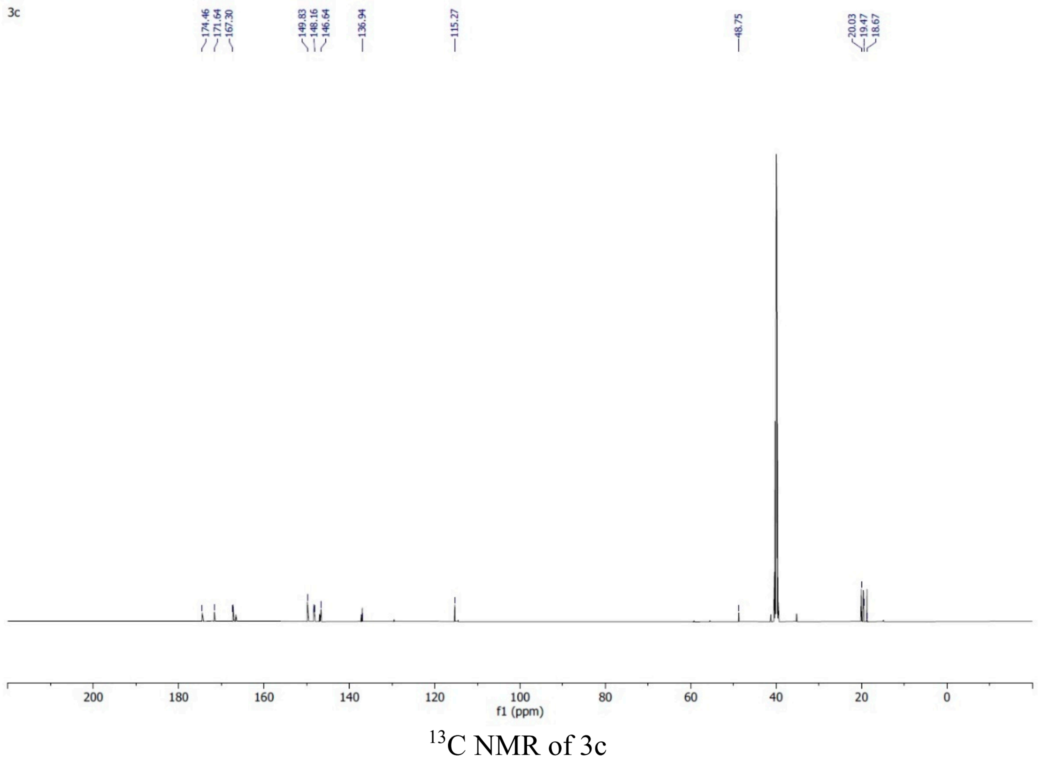<br><br>^{13}C NMR of 3c

4

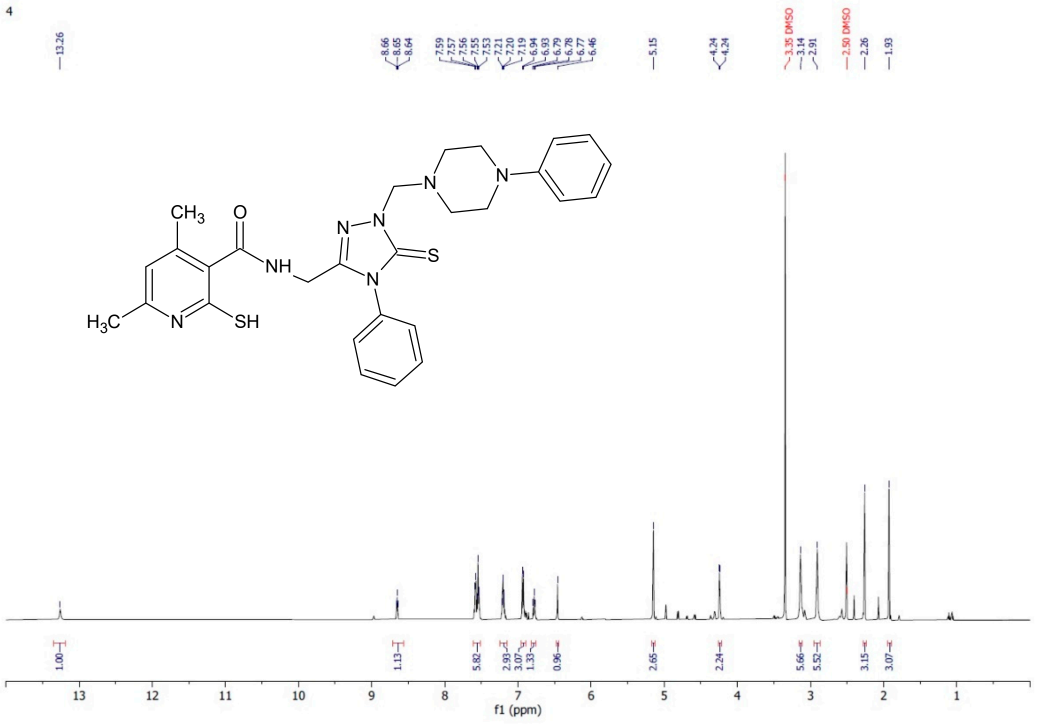

^{1}H NMR of 4

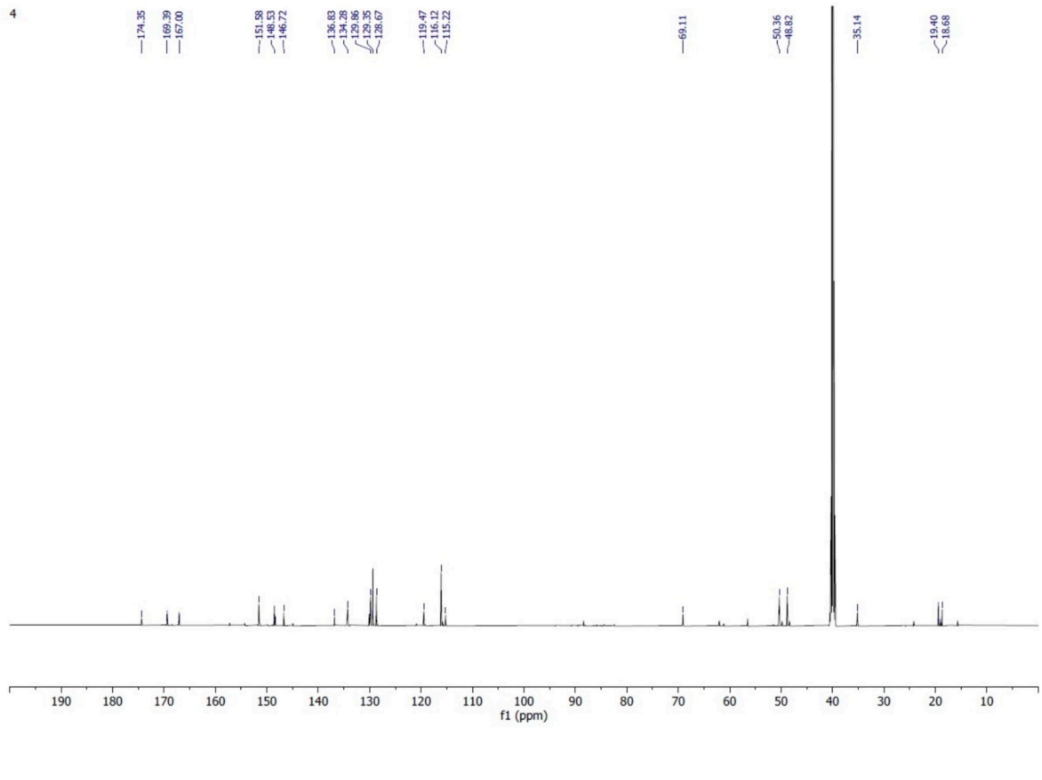

^{13}C NMR of 4

5

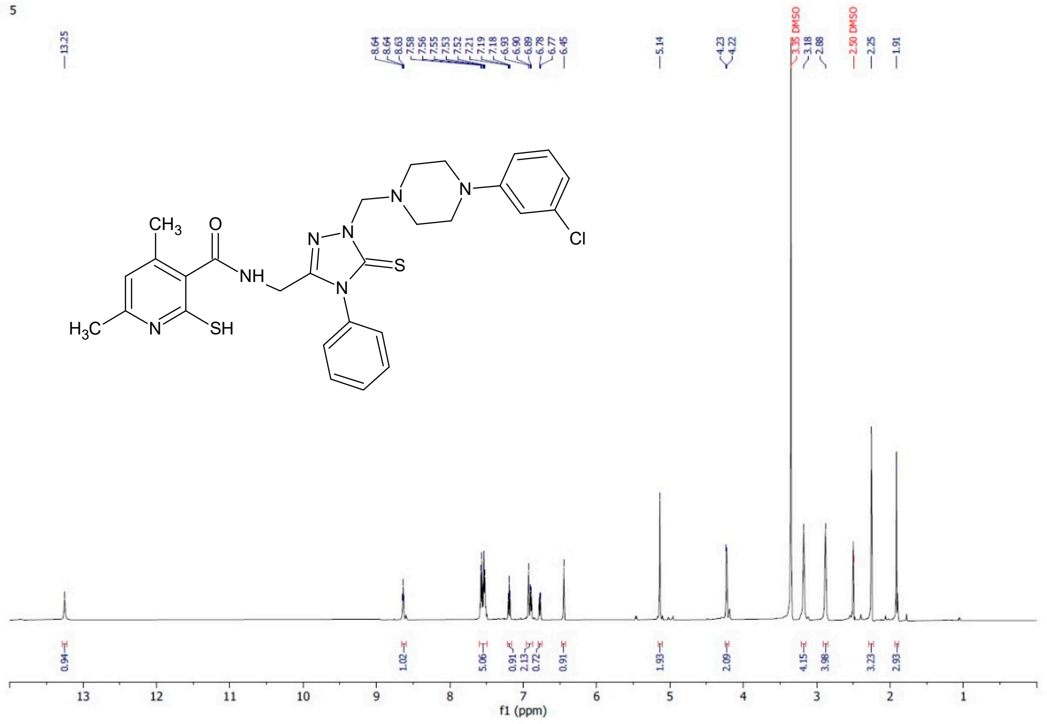

^{1}H NMR of 5

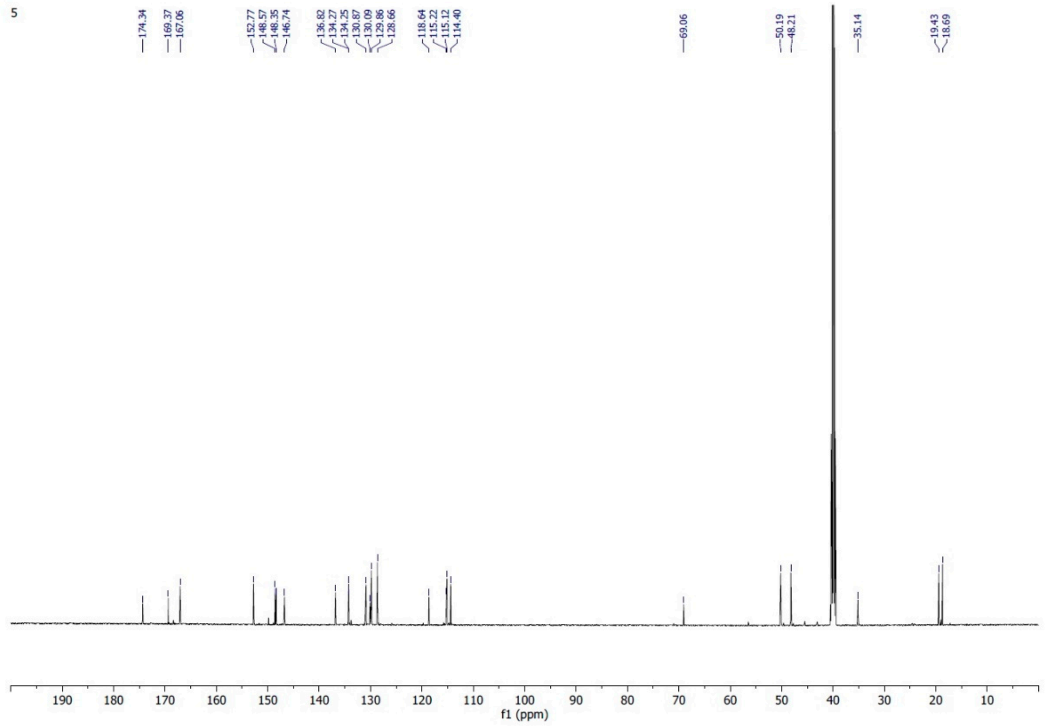

^{13}C NMR of 5

6	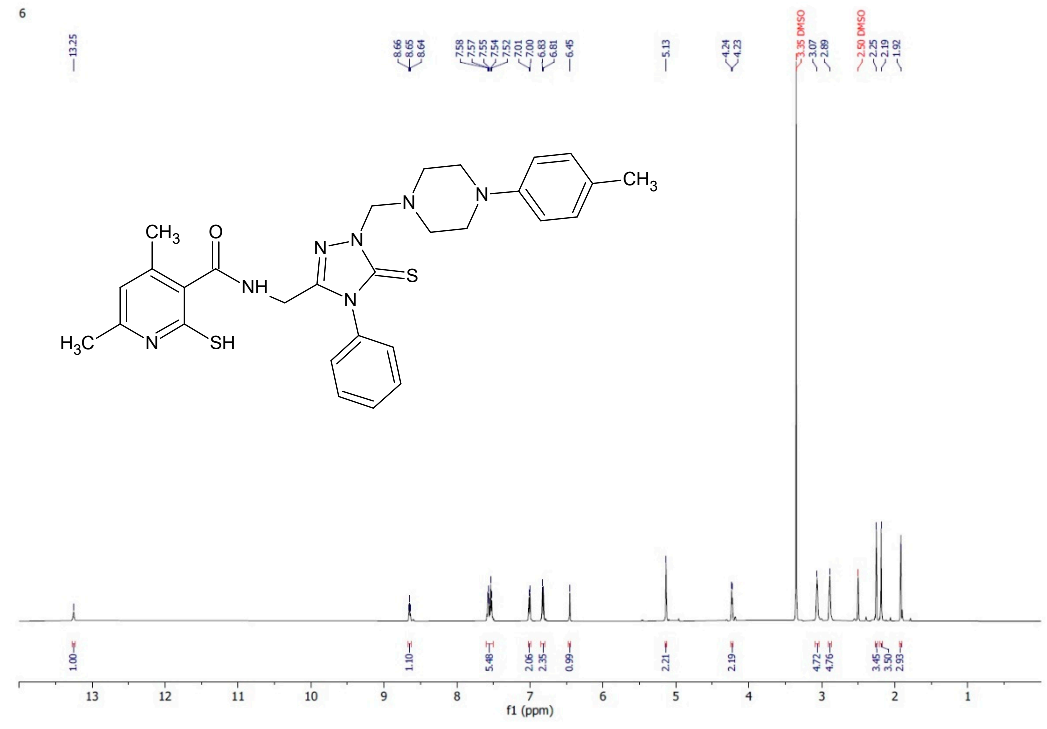 ^{1}H NMR of 6<br>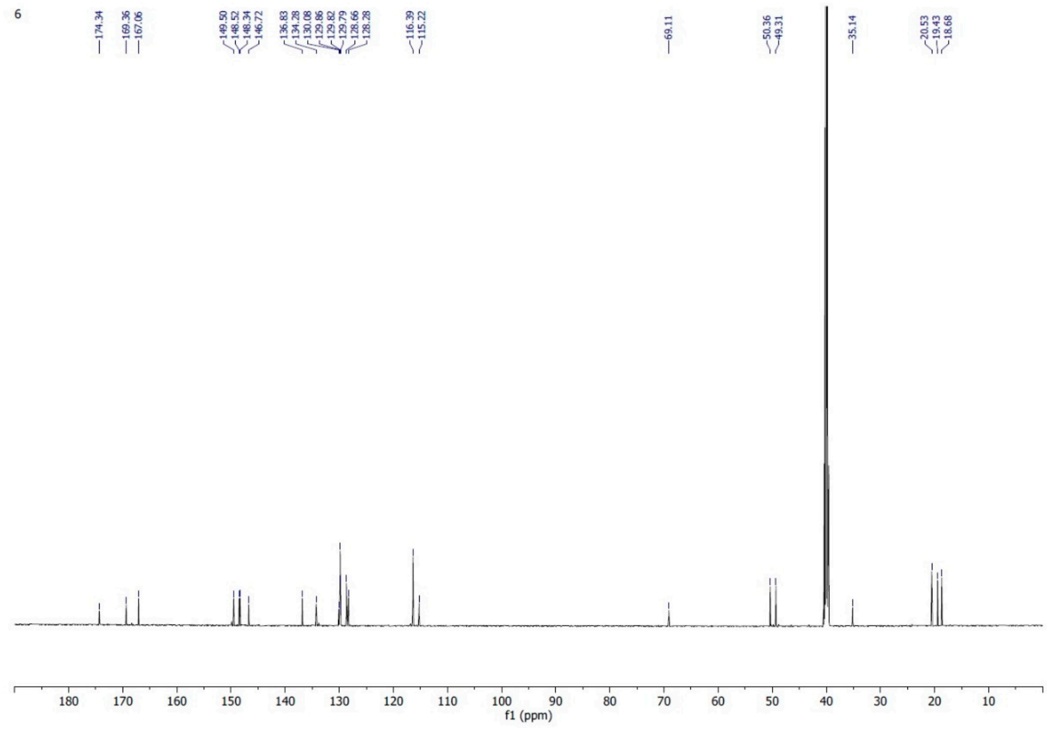 ^{13}C NMR of 6

7

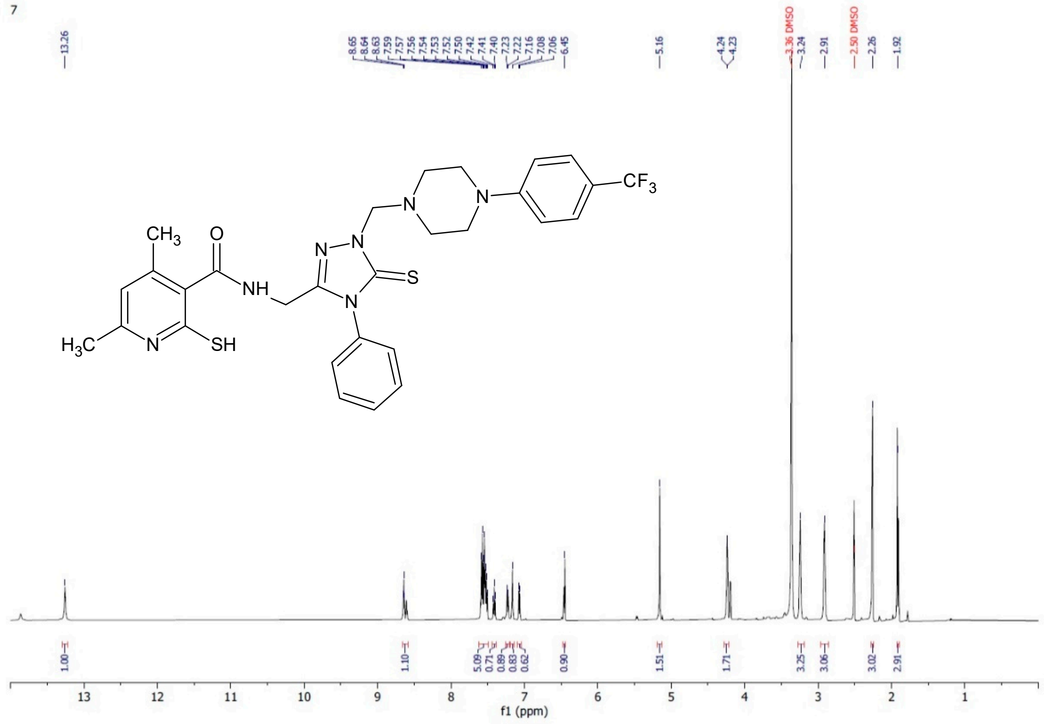

^{1}H NMR of 7

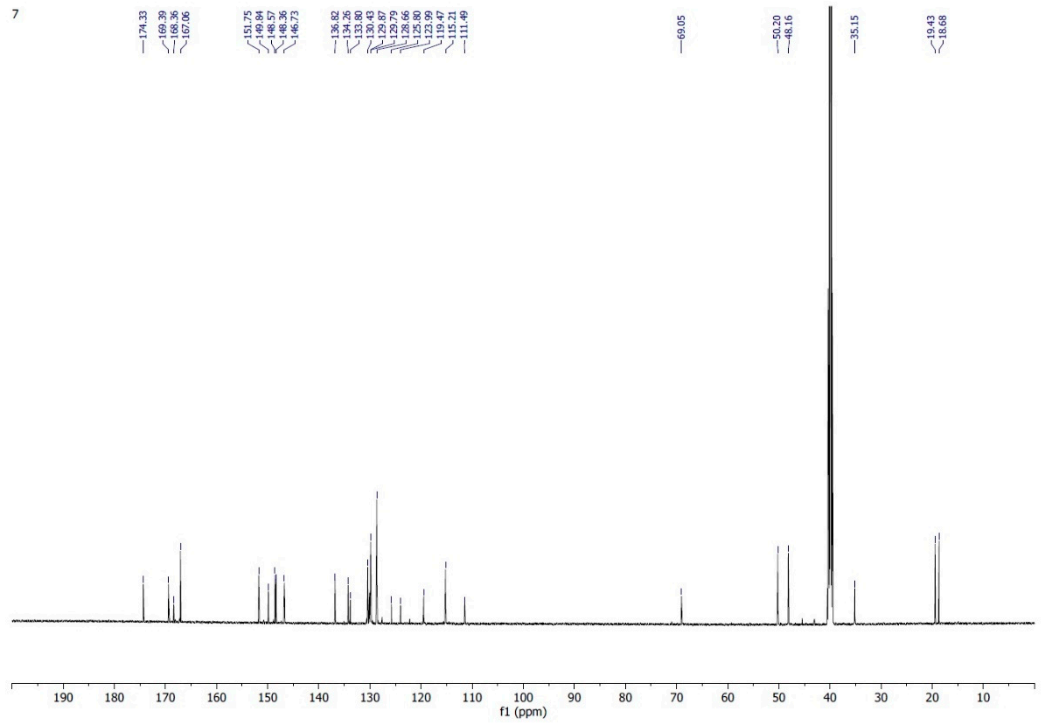

^{13}C NMR for 7

8

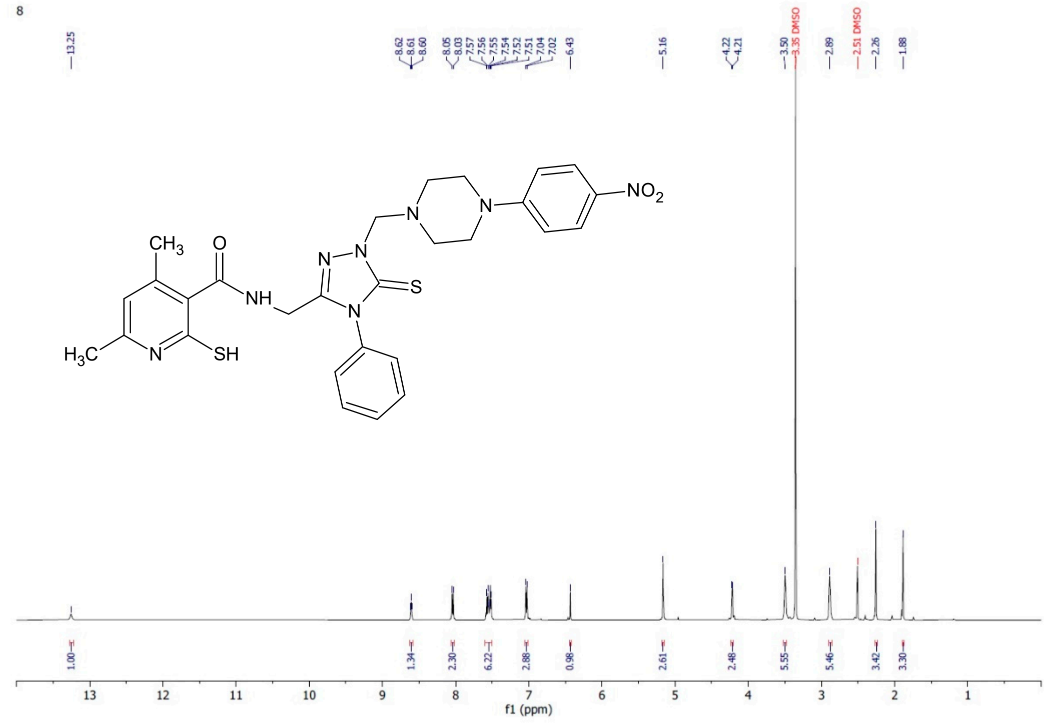

^{1}H NMR for 8

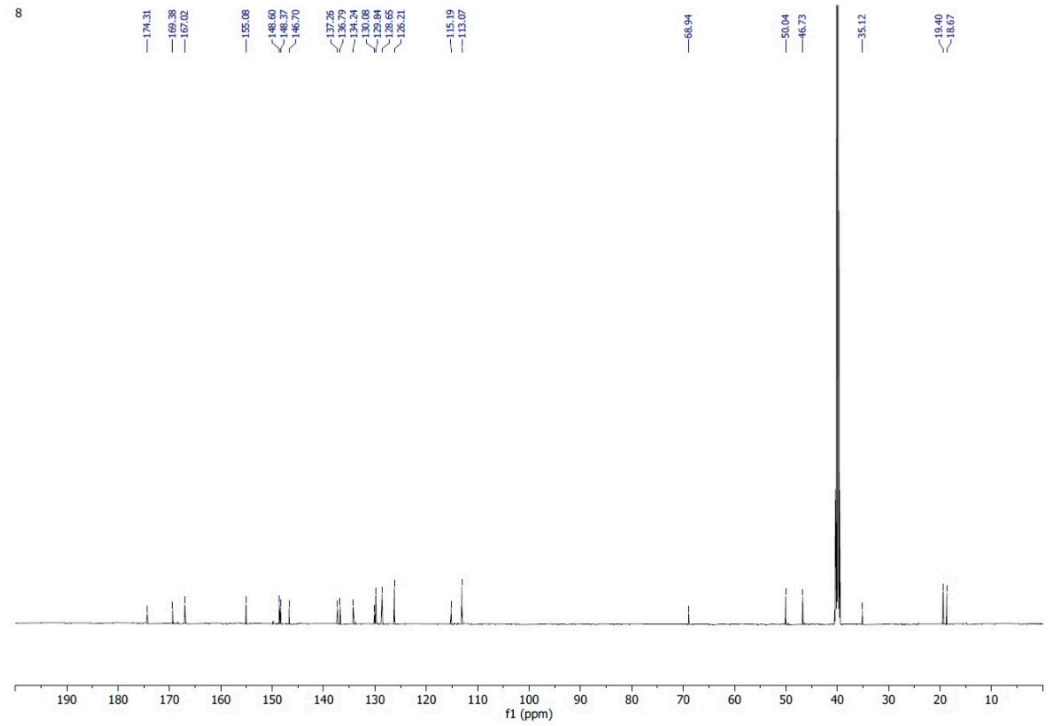

^{13}C NMR for 8

9

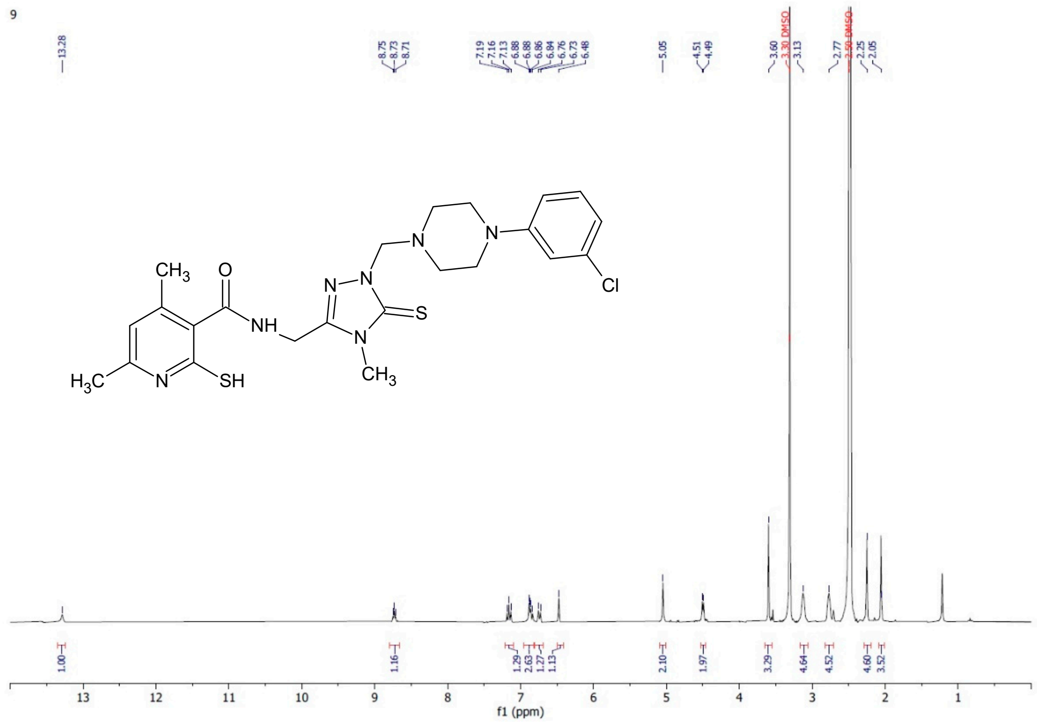

^{1}H NMR for 9

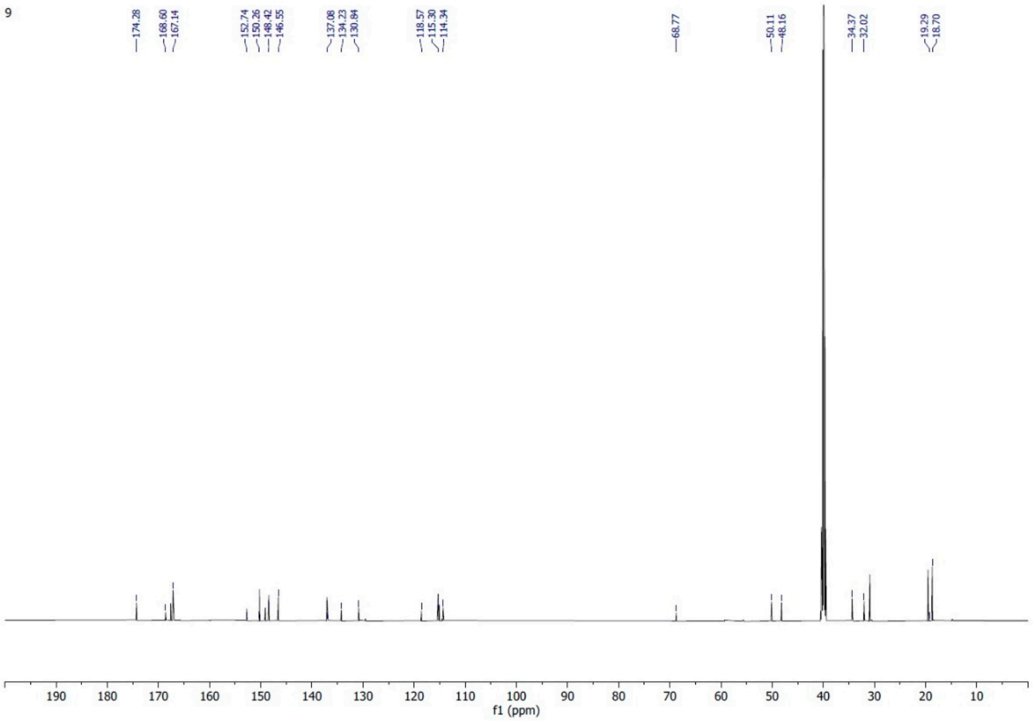

^{13}C NMR for 9

10

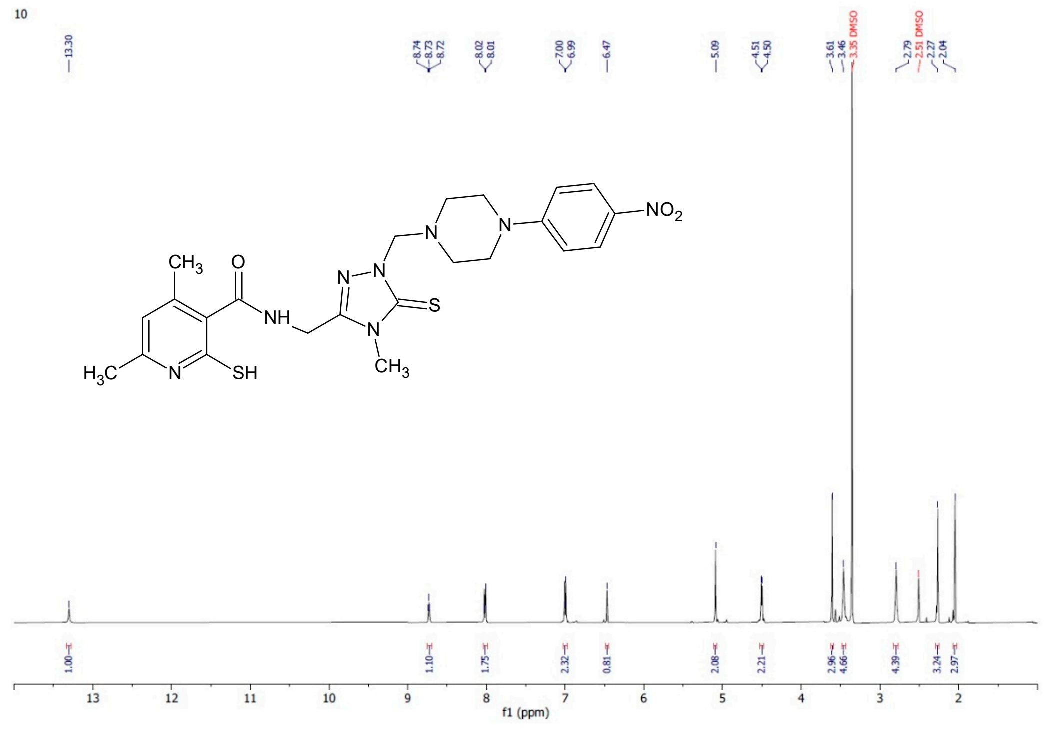

^{1}H NMR for 10

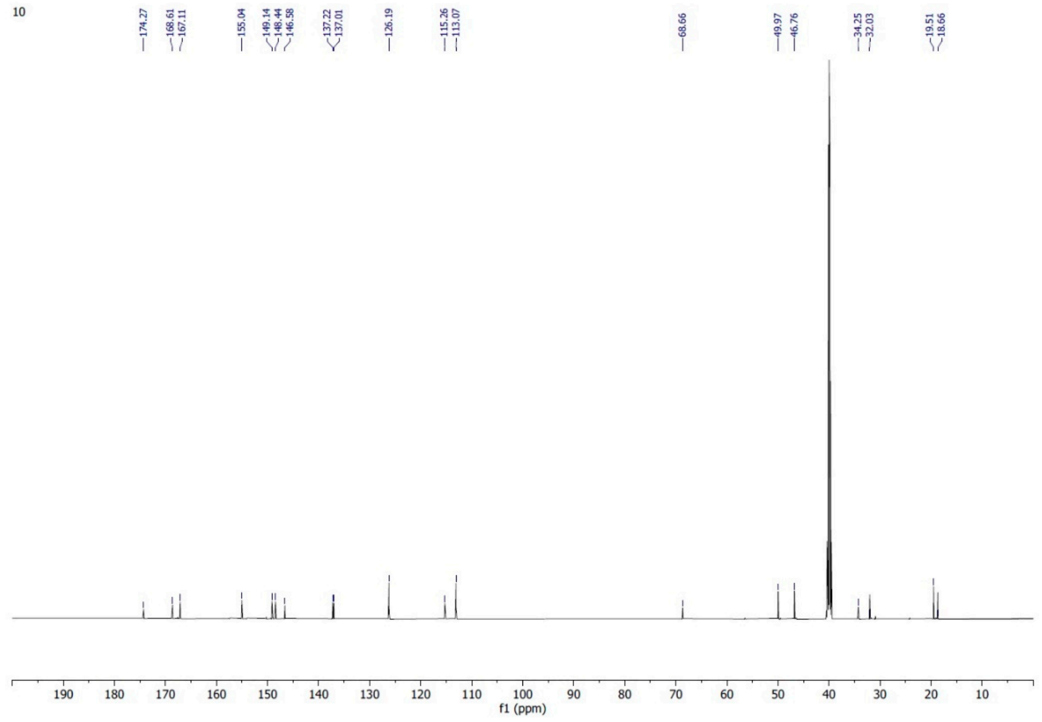

^{13}C NMR for 10

11

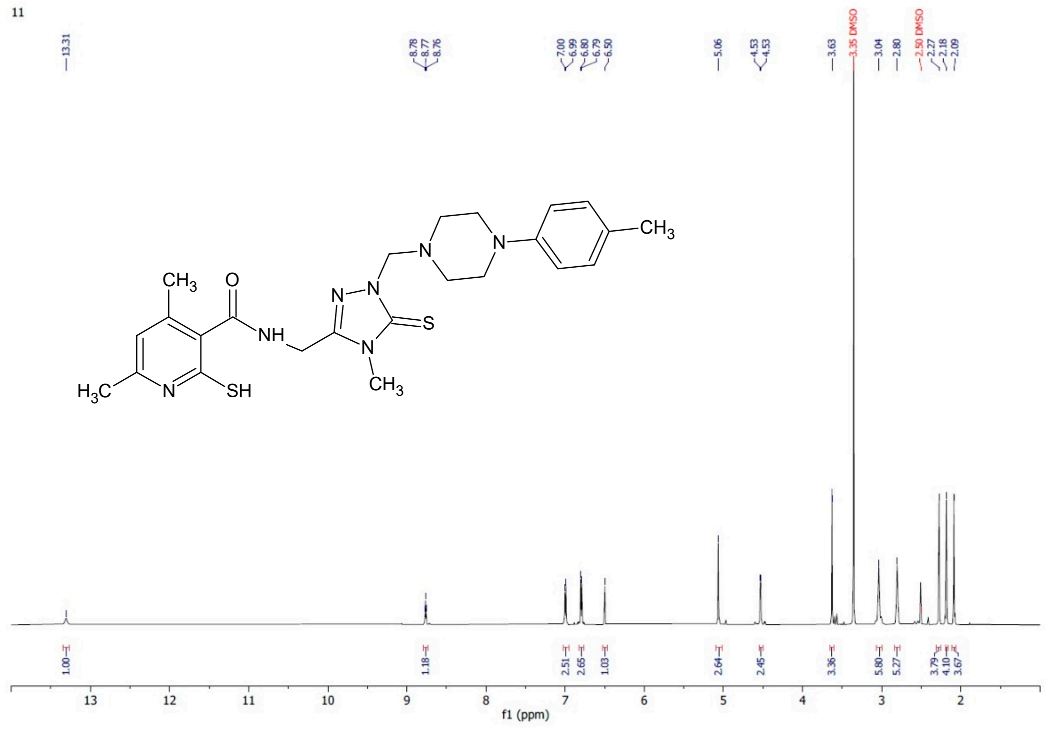

^{1}H NMR for 11

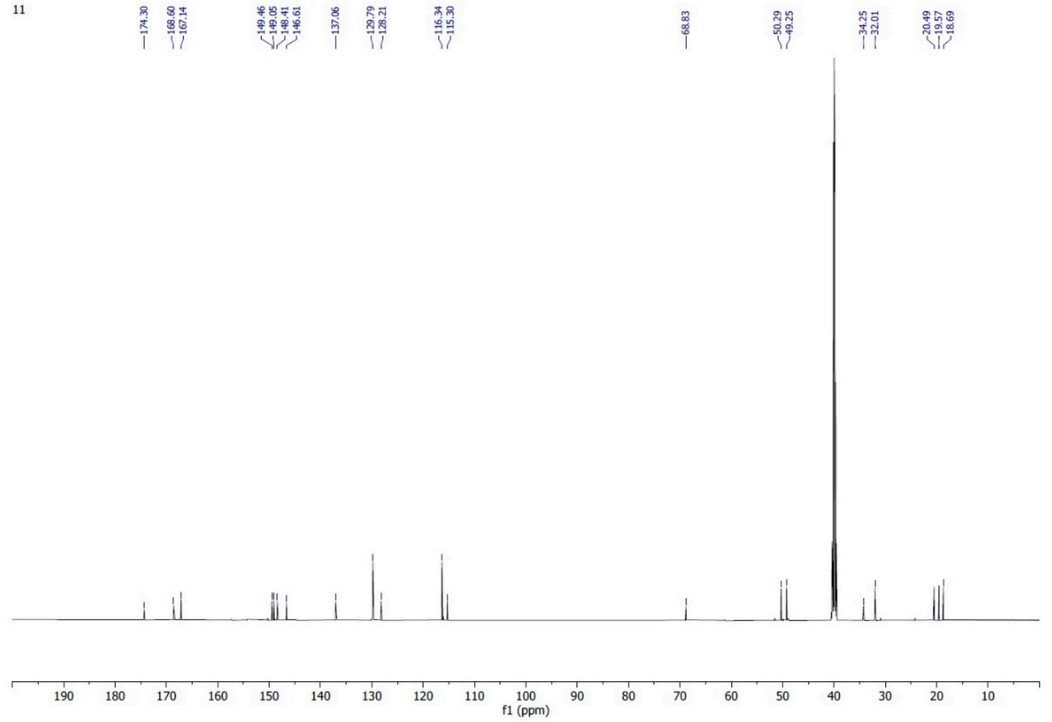

^{13}C NMR for 11

12

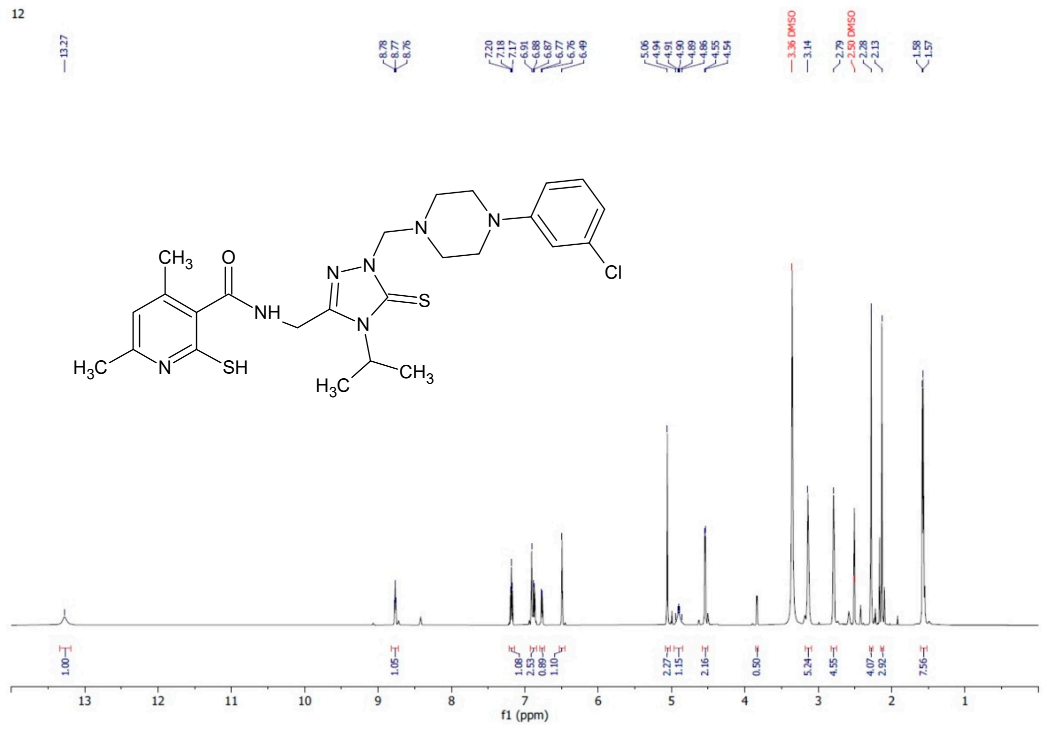

^{1}H NMR for 12

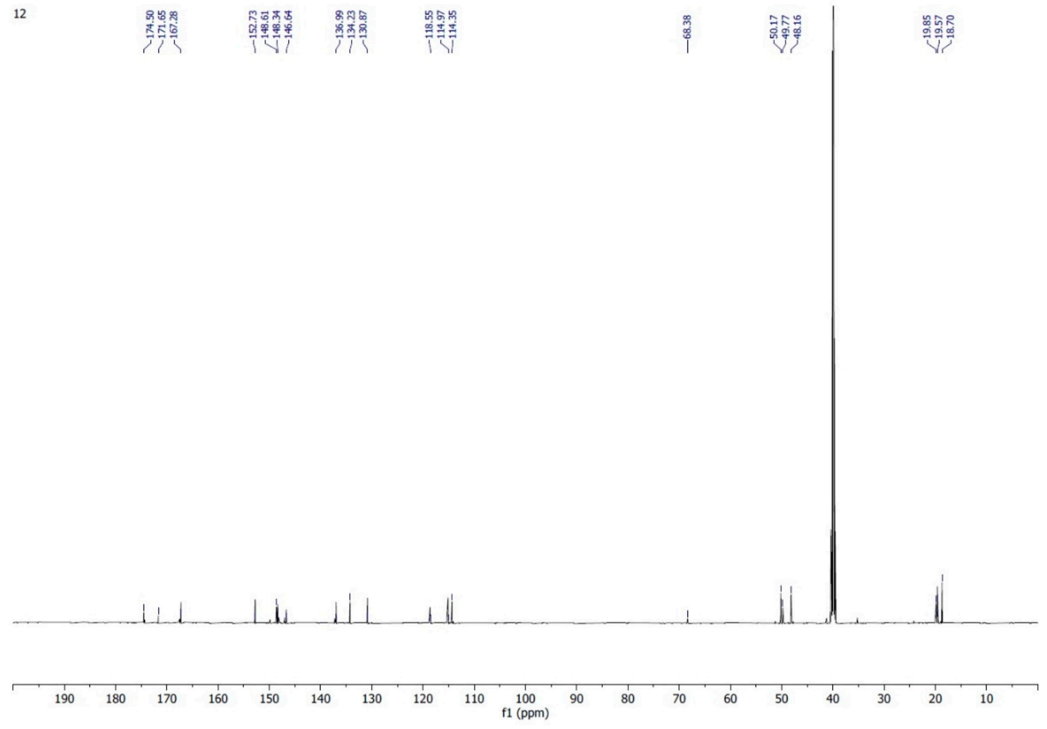

^{13}C NMR for 12

13	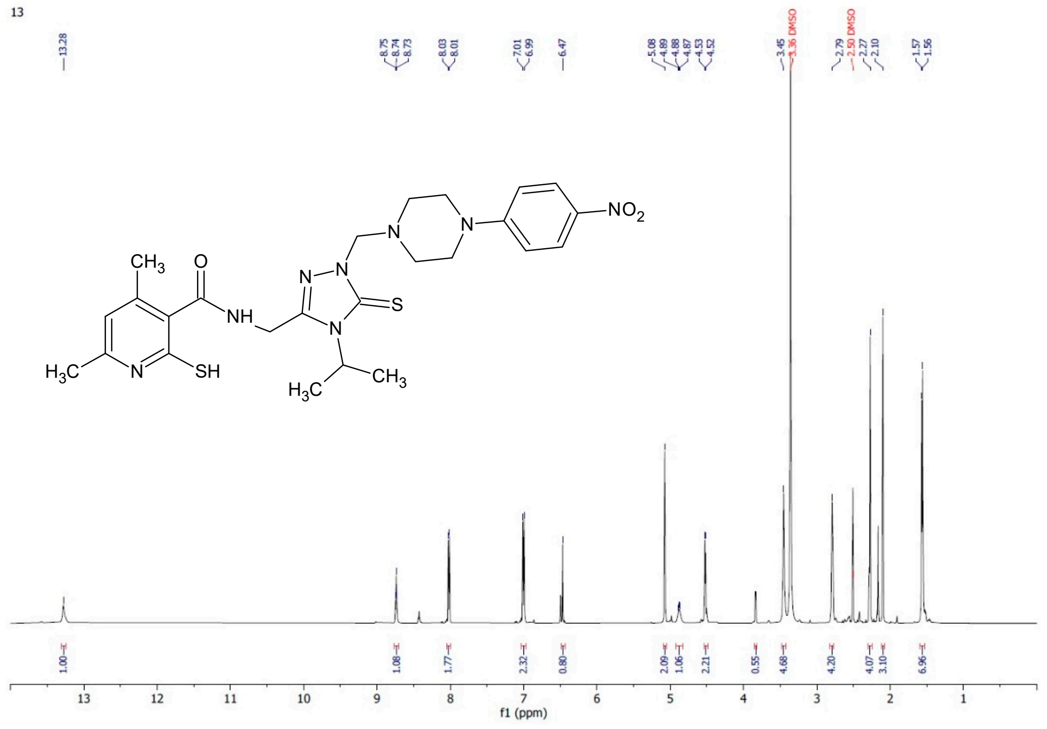 ^{1}H NMR for 13 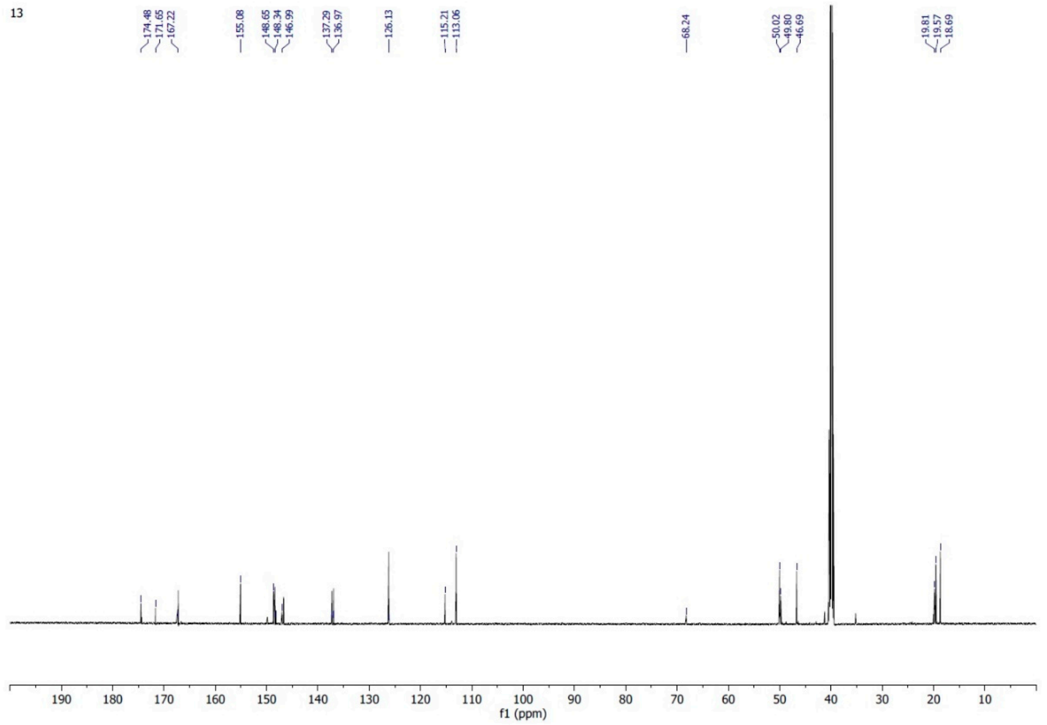 ^{13}C NMR for 13

Table S2. Visualizations of Fourier-Transform Infrared (FT-IR) spectra of compounds **2-13**.

Comp.	ATR-FT-IR spectra
2a	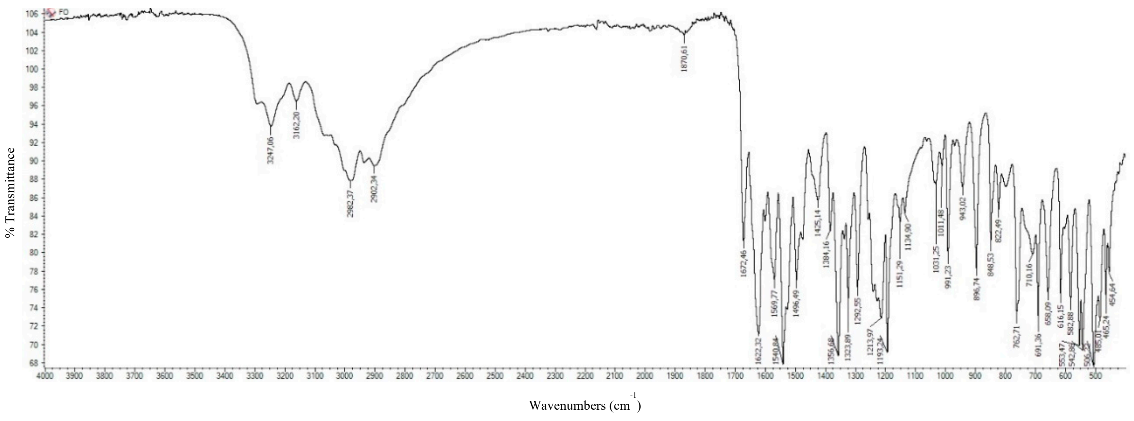
2b	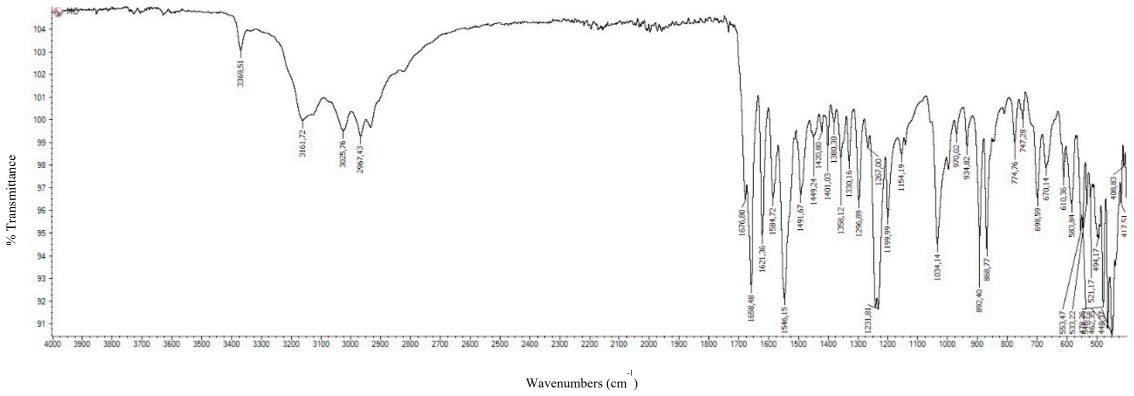
2c	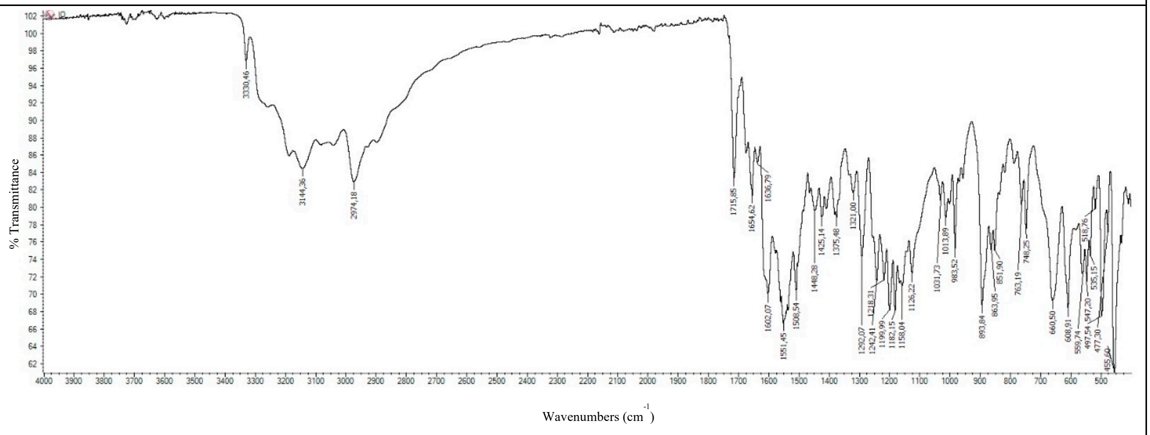

3a

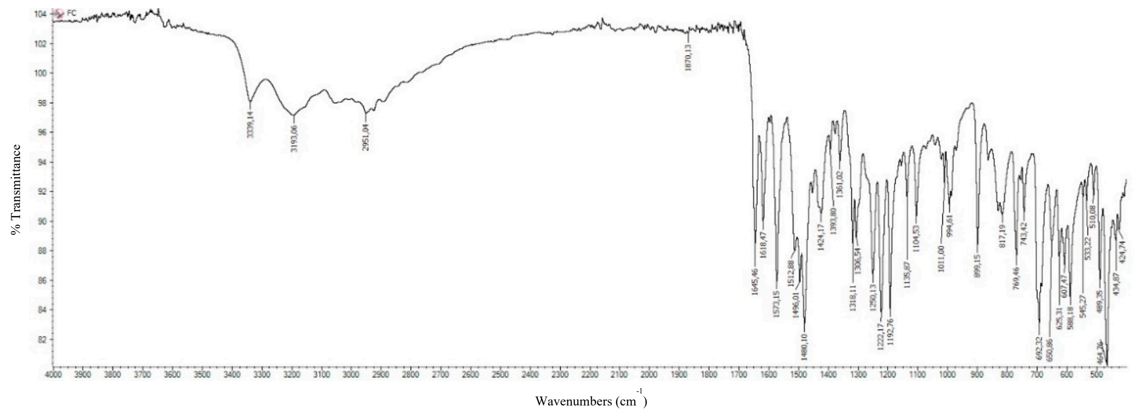

FC
% Transmittance
3339,14
3193,06
2951,04
1870,13
Wavenumbers (cm-1)

3b

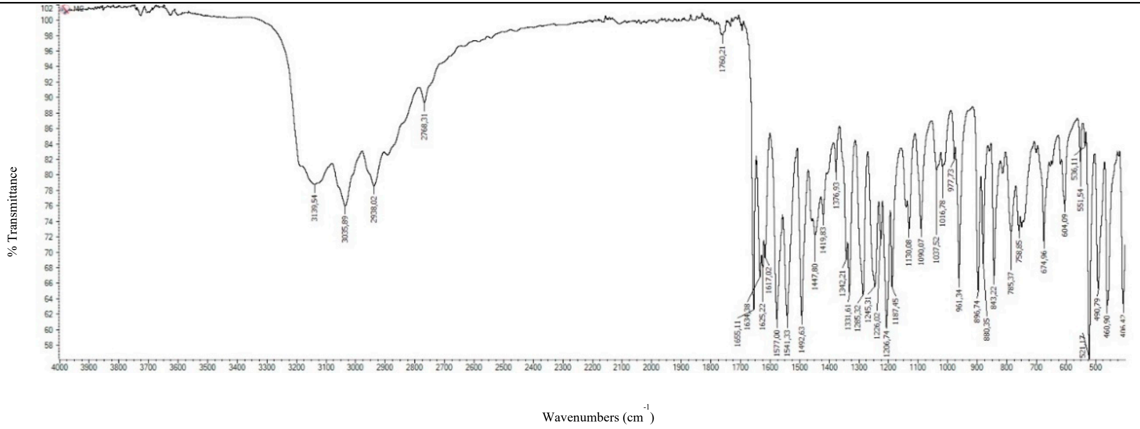

% Transmittance
3139,54
3035,89
2938,02
2768,31
1760,21
Wavenumbers (cm-1)

3c

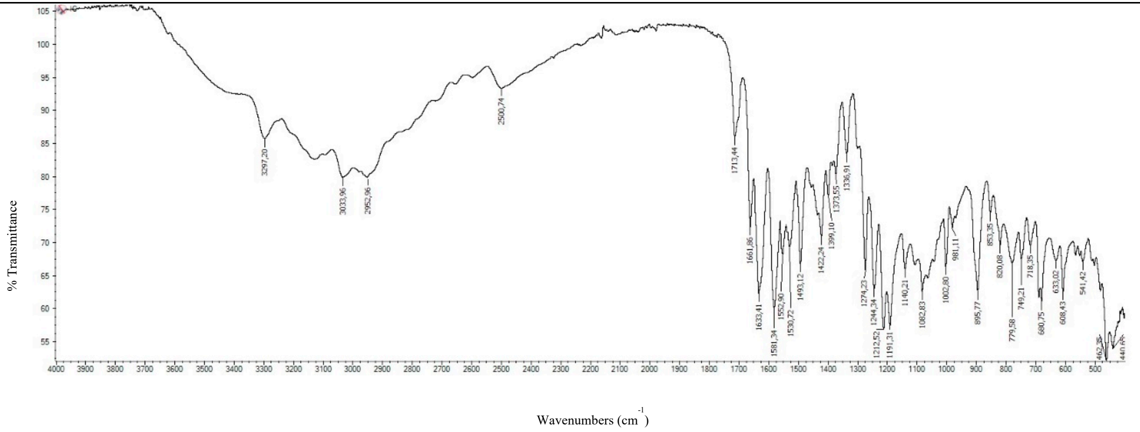

% Transmittance
3297,20
3033,96
2952,96
2500,74
1713,44
Wavenumbers (cm-1)

4

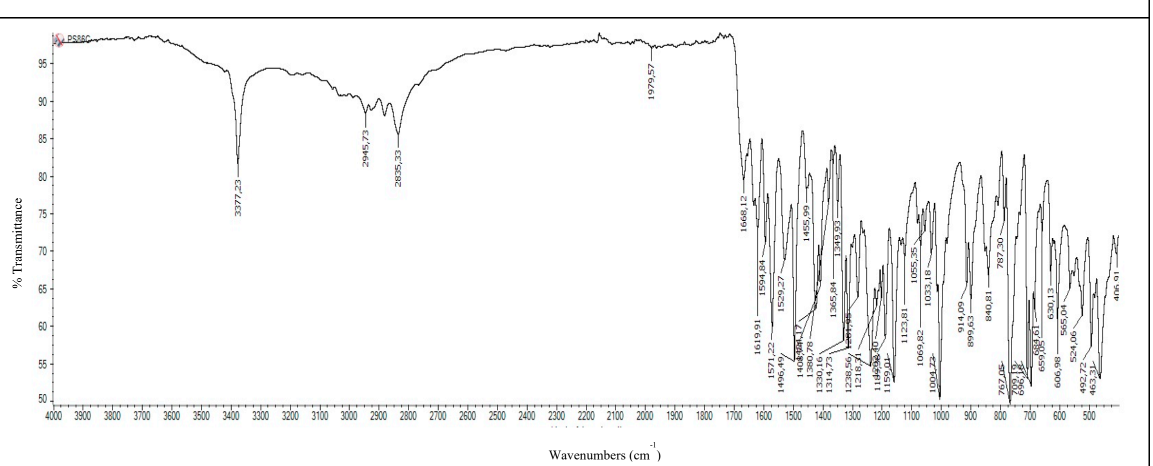

PS86C
% Transmittance
3377,23
2945,73
2835,33
1979,57
1668,12
Wavenumbers (cm-1)

5	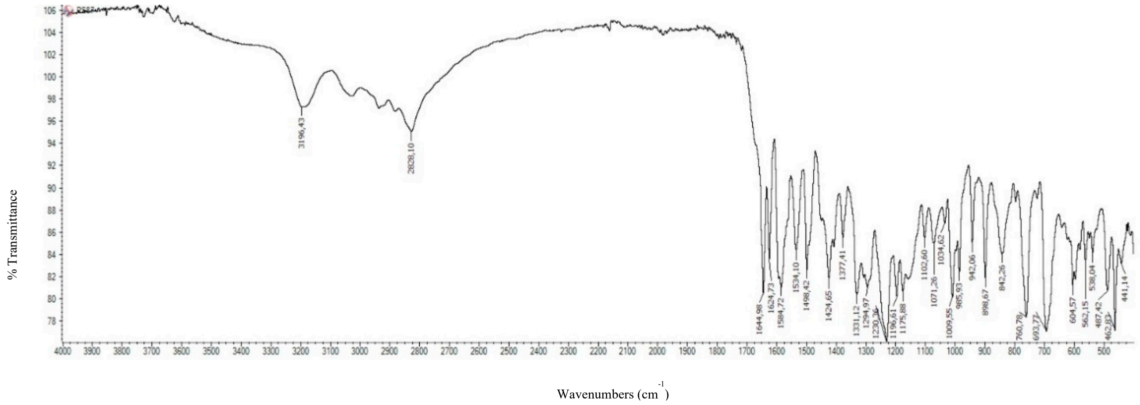% Transmittance<br>Wavenumbers (cm-1)<br>3196,43<br>2828,10
6	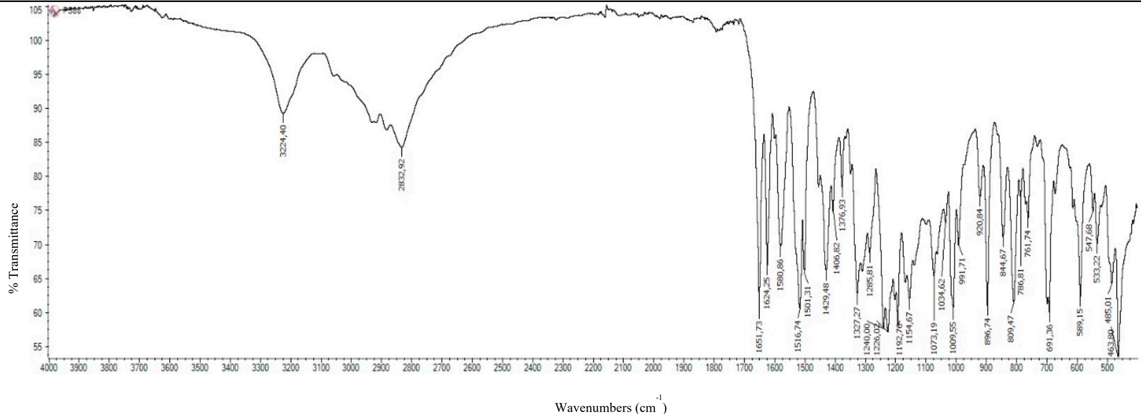% Transmittance<br>Wavenumbers (cm-1)<br>3224,40<br>2832,92
7	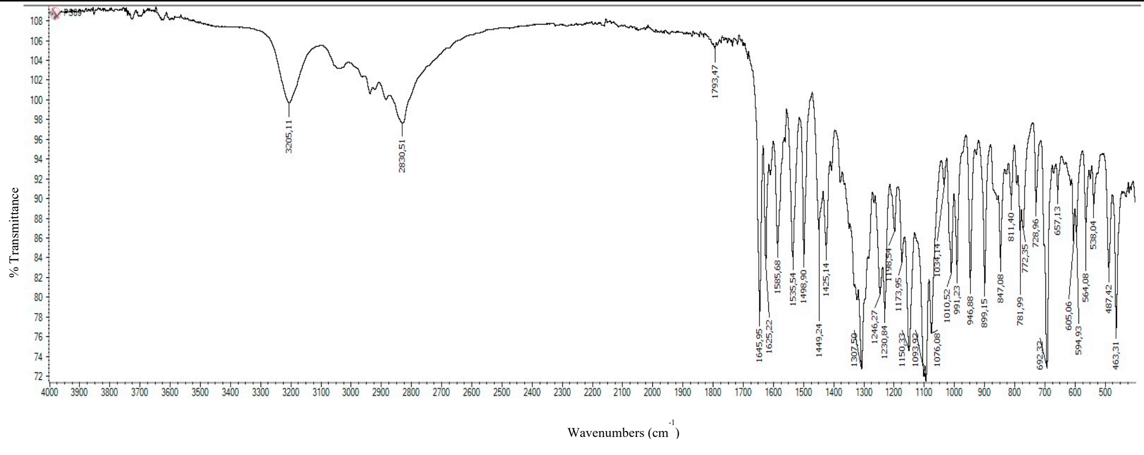% Transmittance<br>Wavenumbers (cm-1)<br>3205,11<br>2830,51<br>1793,47
8	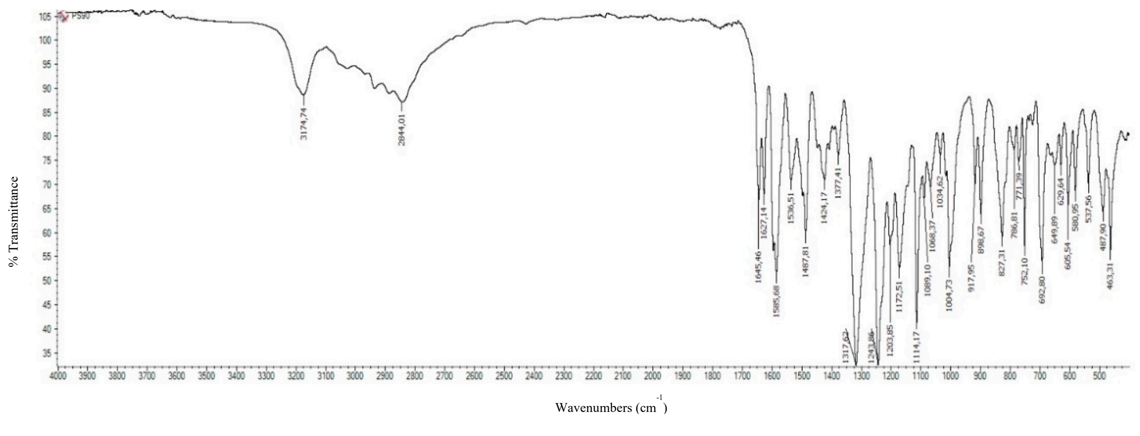% Transmittance<br>Wavenumbers (cm-1)<br>3174,74<br>2844,01

9	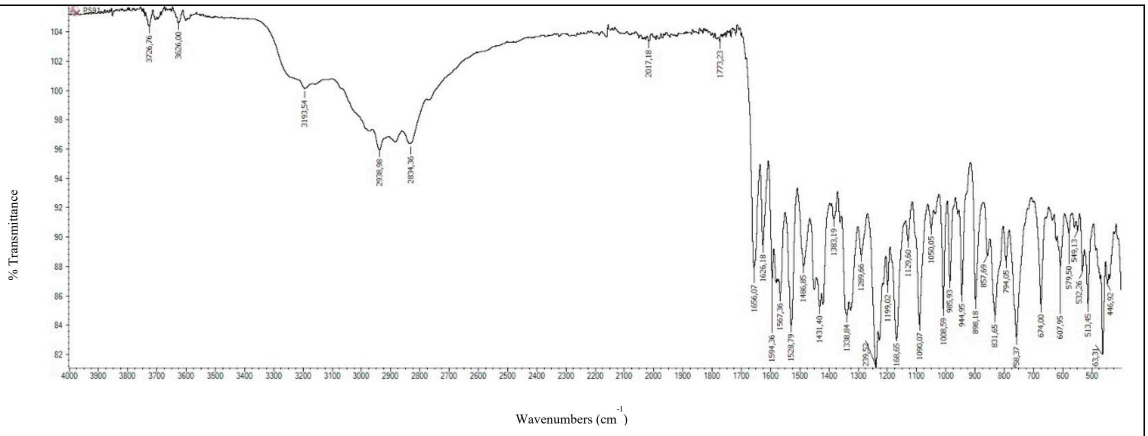
10	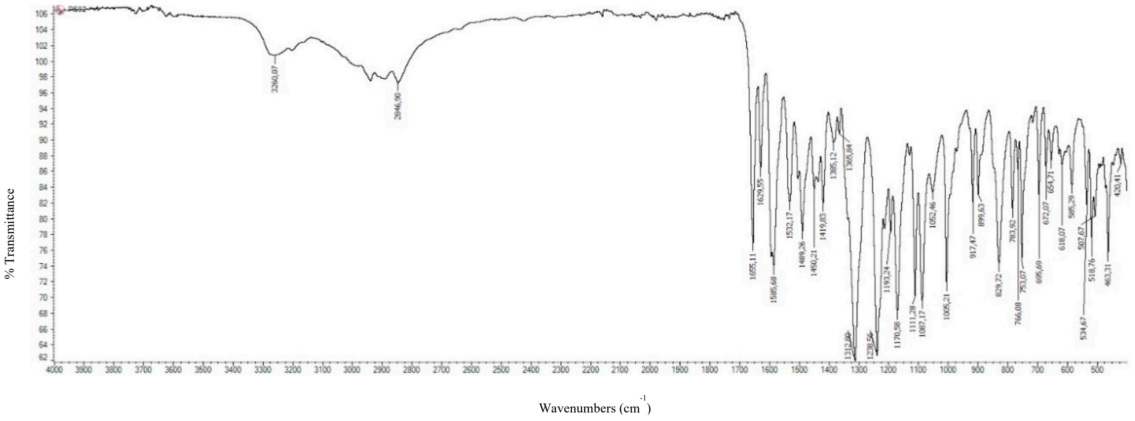
11	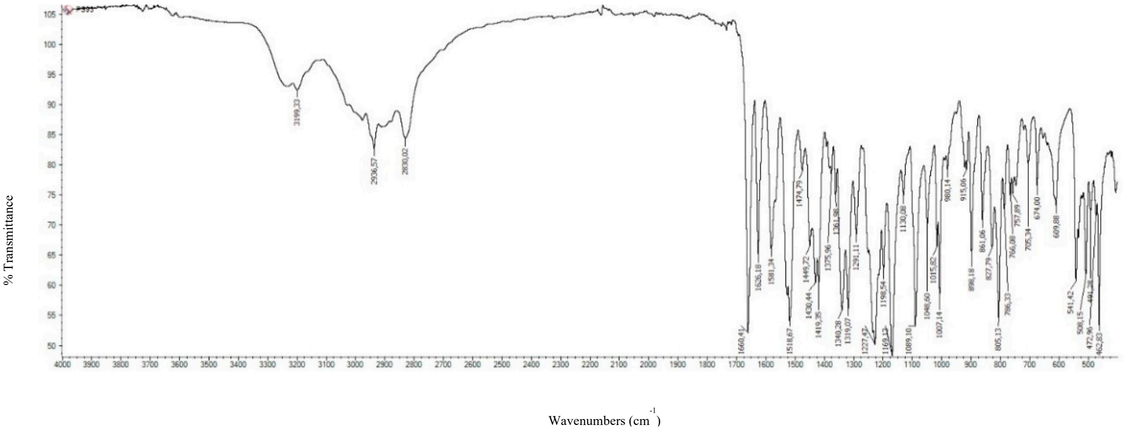
12	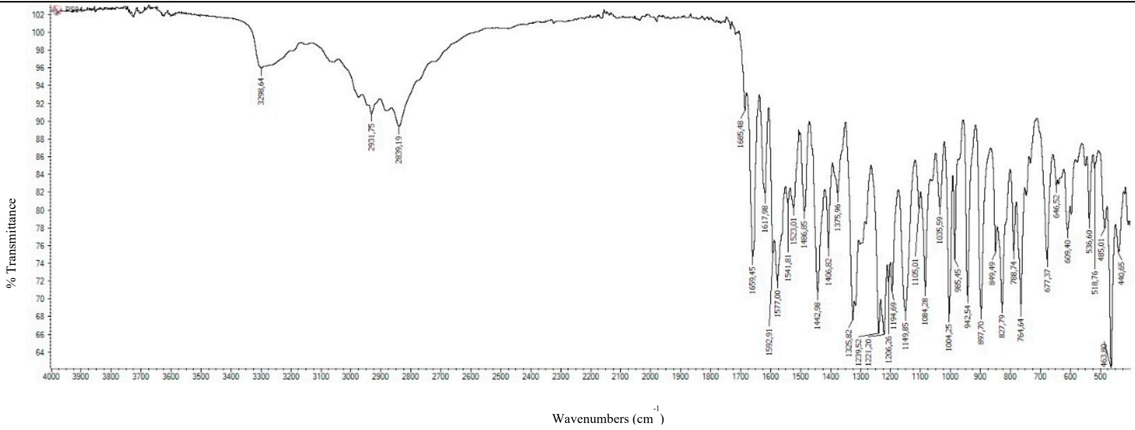

13	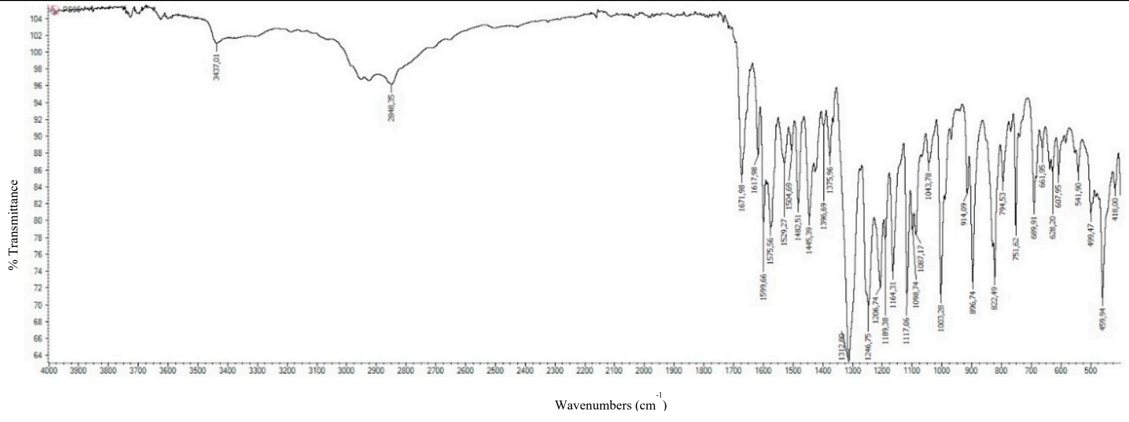 % Transmittance<br>Wavenumbers (cm^{-1})<br>3437,01<br>2846,35<br>1671,98<br>1617,98<br>1599,66<br>1575,56<br>1529,27<br>1504,69<br>1482,51<br>1445,39<br>1396,69<br>1375,96<br>1312,80<br>1246,75<br>1206,74<br>1189,38<br>1164,31<br>1117,06<br>1098,74<br>1087,17<br>1043,78<br>1003,28<br>914,09<br>896,74<br>822,49<br>794,53<br>751,62<br>689,91<br>661,95<br>628,20<br>607,95<br>541,90<br>499,47<br>459,94<br>418,00

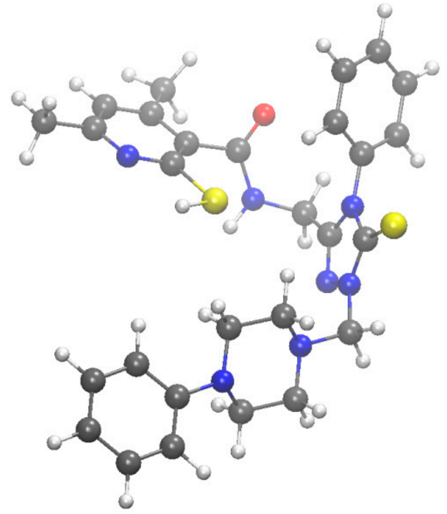

Compound 4

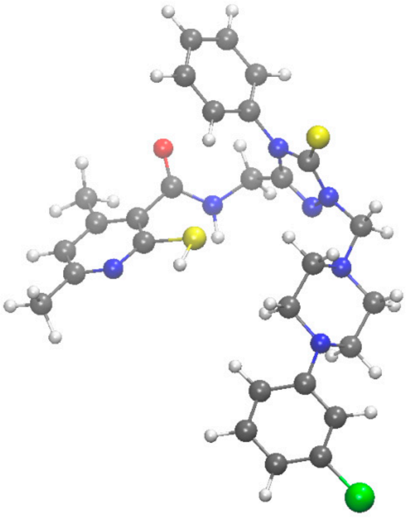

Compound 5

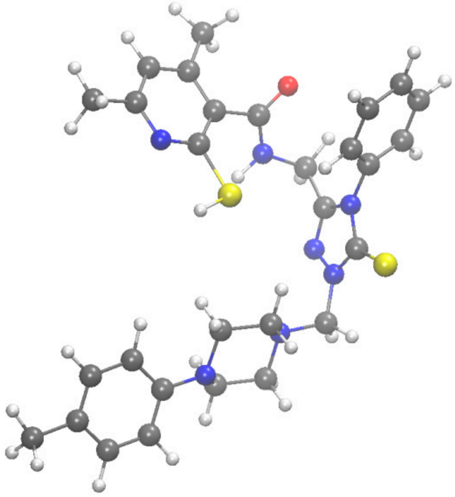

Compound 6

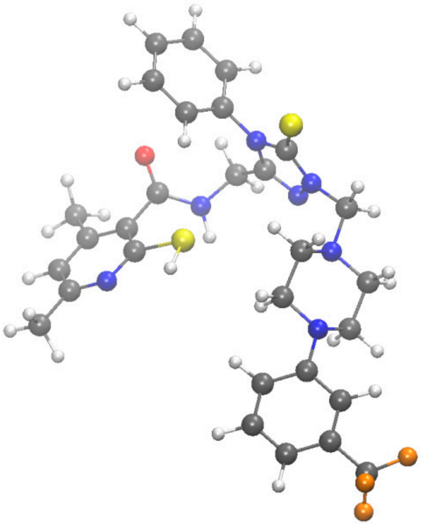

Compound 7

Figure S1. Optimized structures of the *N*-Mannich bases obtained as a result of DFT/ωB97XD/def2-TZVP simulations *in vacuo*. Color coding: carbon—grey, oxygen—red, nitrogen—blue, sulphur—yellow, hydrogen—white, chlorine—green, fluorine—orange.

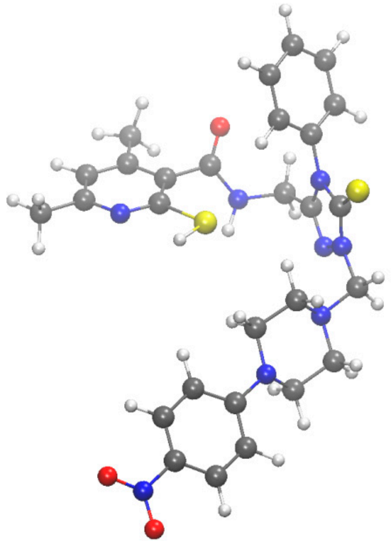

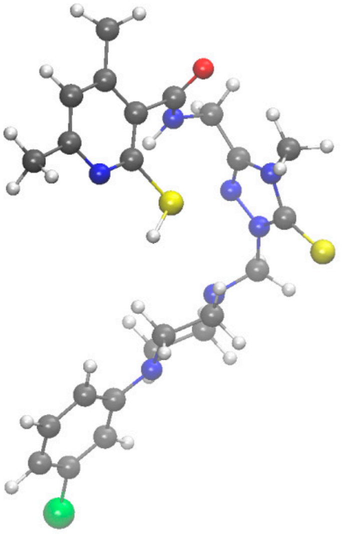

Compound 8 **Compound 9**

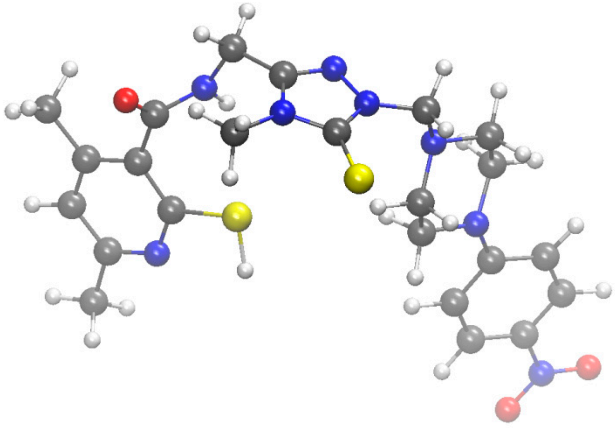

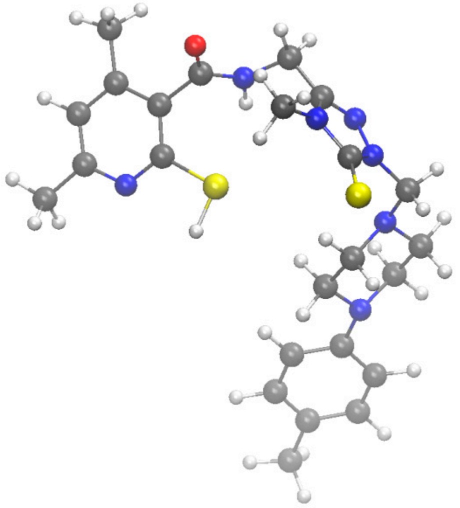

Compound 10 **Compound 11**

Figure S1 (Continuation). Optimized structures of the *N*-Mannich bases obtained as a result of DFT/ωB97XD/def2-TZVP simulations *in vacuo*. Color coding: carbon—grey, oxygen—red, nitrogen—blue, sulphur—yellow, hydrogen—white, chlorine—green, fluorine—orange.

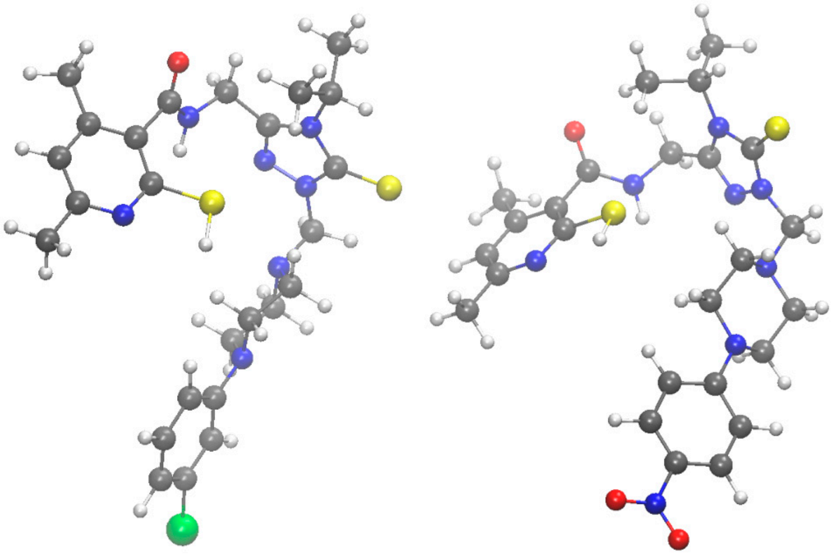

Compound 12 **Compound 13**

Figure S1 (Continuation). Optimized structures of the *N*-Mannich bases obtained as a result of DFT/ωB97XD/def2-TZVP simulations *in vacuo*. Color coding: carbon—grey, oxygen—red, nitrogen—blue, sulphur—yellow, hydrogen—white, chlorine—green, fluorine—orange.

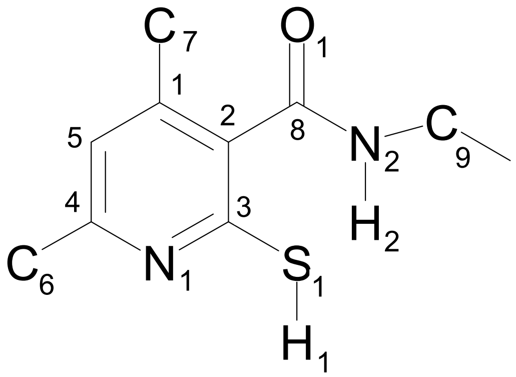

Scheme S1. Core part of the studied *N*-Mannich bases. Some hydrogen atoms are omitted for clarity. The atom numbering scheme was prepared especially for the study and QTAIM analyses.

Table S3. Selected metric parameters (see Scheme S1) of five investigated compounds (denoted as **4**, **6**, **8**, **11** and **13**) obtained at the DFT/ωB97XD/Def2-TZVP level of theory *in vacuo* and using PCM solvation model.

**Metric parameters**	***in vacuo***	**IEF-PCM/water**
	**Compound 4**	
**Bond length in [Å]**		
**C1-C2**	1.3972	1.3950
**C2-C3**	1.3989	1.3966
**C3-N1**	1.3237	1.3239
**N1-C4**	1.3315	1.3348
**C4-C5**	1.3863	1.3867
**C5-C1**	1.3870	1.3875
**C4-C6**	1.4984	1.4981
**C1-C7**	1.5003	1.4998
**C3-S1**	1.7781	1.7774
**S1-H1**	1.3384	1.3384
**C2-C8**	1.5022	1.5014
**C8-O1**	1.2128	1.2188
**C8-N2**	1.3606	1.3512
**N2-H2**	1.0068	1.0068
**N2-C9**	1.4482	1.4469
**Valence angle [°]**		
**C3-S1-H1**	93.2	94.3
**C2-C8-O1**	122.0	122.0
**C2-C8-N2**	115.0	114.5
**C8-N2-C9**	122.5	123.6
**Dihedral angle [°]**		
**C7-C1-C2-C8**	2.3	2.3
**C1-C2-C8-O1**	52.0	63.4
**C2-C8-N2-C9**	-168.7	-171.5
**O1-C8-N2-H2**	177.4	-178.4

Table S3 (Continuation). Selected metric parameters (see Scheme S1) of five investigated compounds (denoted as **4**, **6**, **8**, **11** and **13**) obtained at the DFT/ωB97XD/Def2-TZVP level of theory *in vacuo* and using PCM solvation model.

**Metric parameters**	***in vacuo***	**IEF-PCM/water**
	**Compound 6**	
**Bond length in [Å]**		
**C1-C2**	1.3973	1.3953
**C2-C3**	1.3990	1.3969
**C3-N1**	1.3237	1.3240
**N1-C4**	1.3314	1.3347
**C4-C5**	1.3863	1.3868
**C5-C1**	1.3871	1.3874
**C4-C6**	1.4984	1.4981
**C1-C7**	1.5003	1.4998
**C3-S1**	1.7780	1.7778
**S1-H1**	1.3384	1.3383
**C2-C8**	1.5022	1.5013
**C8-O1**	1.2129	1.2190
**C8-N2**	1.3606	1.3512
**N2-H2**	1.0068	1.0067
**N2-C9**	1.4482	1.4470
**Valence angle [°]**		
**C3-S1-H1**	93.2	94.2
**C2-C8-O1**	122.0	122.0
**C2-C8-N2**	115.0	114.5
**C8-N2-C9**	122.5	123.7
**Dihedral angle [°]**		
**C7-C1-C2-C8**	2.3	2.5
**C1-C2-C8-O1**	51.6	62.2
**C2-C8-N2-C9**	-168.6	-171.5
**O1-C8-N2-H2**	177.3	-177.9

Table S3 (Continuation). Selected metric parameters (see Scheme S1) of five investigated compounds (denoted as **4**, **6**, **8**, **11** and **13**) obtained at the DFT/ωB97XD/Def2-TZVP level of theory *in vacuo* and using PCM solvation model.

**Metric parameters**	***in vacuo***	**IEF-PCM/water**
	**Compound 8**	
**Bond length in [Å]**		
**C1-C2**	1.3973	1.3955
**C2-C3**	1.3982	1.3968
**C3-N1**	1.3233	1.3240
**N1-C4**	1.3317	1.3346
**C4-C5**	1.3865	1.3867
**C5-C1**	1.3869	1.3873
**C4-C6**	1.4982	1.4980
**C1-C7**	1.5002	1.4998
**C3-S1**	1.7792	1.7781
**S1-H1**	1.3385	1.3383
**C2-C8**	1.5020	1.5011
**C8-O1**	1.2125	1.2188
**C8-N2**	1.3609	1.3512
**N2-H2**	1.0066	1.0066
**N2-C9**	1.4479	1.4468
**Valence angle [°]**		
**C3-S1-H1**	93.1	94.2
**C2-C8-O1**	122.1	122.0
**C2-C8-N2**	114.9	114.5
**C8-N2-C9**	122.6	123.7
**Dihedral angle [°]**		
**C7-C1-C2-C8**	2.4	2.5
**C1-C2-C8-O1**	52.8	61.6
**C2-C8-N2-C9**	-168.7	-171.9
**O1-C8-N2-H2**	177.9	-177.7

Table S3 (Continuation). Selected metric parameters (see Scheme S1) of five investigated compounds (denoted as **4**, **6**, **8**, **11** and **13**) obtained at the DFT/ωB97XD/Def2-TZVP level of theory *in vacuo* and using PCM solvation model.

**Metric parameters**	***in vacuo***	**IEF-PCM/water**
	**Compound 11**	
**Bond length in [Å]**		
**C1-C2**	1.3951	1.3941
**C2-C3**	1.3981	1.3963
**C3-N1**	1.3232	1.3238
**N1-C4**	1.3321	1.3351
**C4-C5**	1.3870	1.3870
**C5-C1**	1.3870	1.3875
**C4-C6**	1.4984	1.4981
**C1-C7**	1.5001	1.4997
**C3-S1**	1.7763	1.7771
**S1-H1**	1.3381	1.3381
**C2-C8**	1.5004	1.5003
**C8-O1**	1.2156	1.2209
**C8-N2**	1.3577	1.3494
**N2-H2**	1.0068	1.0068
**N2-C9**	1.4491	1.4480
**Valence angle [°]**		
**C3-S1-H1**	93.5	94.4
**C2-C8-O1**	122.3	122.1
**C2-C8-N2**	114.7	114.5
**C8-N2-C9**	122.9	123.9
**Dihedral angle [°]**		
**C7-C1-C2-C8**	2.0	2.2
**C1-C2-C8-O1**	58.5	66.4
**C2-C8-N2-C9**	-168.8	-171.2
**O1-C8-N2-H2**	176.4	-179.1

Table S3 (Continuation). Selected metric parameters (see Scheme S1) of five investigated compounds (denoted as **4**, **6**, **8**, **11** and **13**) obtained at the DFT/ωB97XD/Def2-TZVP level of theory *in vacuo* and using PCM solvation model.

**Metric parameters**	***in vacuo***	**IEF-PCM/water**
	**Compound 13**	
**Bond length in [Å]**		
**C1-C2**	1.3958	1.3943
**C2-C3**	1.3976	1.3964
**C3-N1**	1.3232	1.3241
**N1-C4**	1.3321	1.3350
**C4-C5**	1.3870	1.3869
**C5-C1**	1.3870	1.3875
**C4-C6**	1.4982	1.4981
**C1-C7**	1.5001	1.4997
**C3-S1**	1.7772	1.7767
**S1-H1**	1.3385	1.3385
**C2-C8**	1.5010	1.5009
**C8-O1**	1.2154	1.2208
**C8-N2**	1.3557	1.3483
**N2-H2**	1.0070	1.0070
**N2-C9**	1.4480	1.4473
**Valence angle [°]**		
**C3-S1-H1**	93.2	94.2
**C2-C8-O1**	122.4	122.1
**C2-C8-N2**	114.5	114.3
**C8-N2-C9**	123.3	124.0
**Dihedral angle [°]**		
**C7-C1-C2-C8**	1.7	1.9
**C1-C2-C8-O1**	57.2	65.8
**C2-C8-N2-C9**	-171.1	-172.2
**O1-C8-N2-H2**	179.6	-177.5

Table S4. Partial atomic charges for selected atoms (see Scheme S1) according to the QTAIM theory (the data is given for the gas phase results).

Compound	qC1	qC2	qC3	qN1	qC4	qC5	qC6	qC7	qS1	qH1	qC8	qO1	qN2	qH2	qC9
**4**	0.0113	-0.0330	0.4354	-1.1891	0.5379	-0.0379	-0.0034	-0.0003	0.0417	0.0414	1.4996	-1.2041	-1.1820	0.4262	0.3890
**5**	0.0112	-0.0328	0.4358	-1.1893	0.5382	-0.0376	-0.0032	-0.0006	0.0407	0.0402	1.4997	-1.2038	-1.1820	0.4259	0.3894
**6**	0.0110	-0.0327	0.4352	-1.1892	0.5384	-0.0378	-0.0033	-0.0002	0.0417	0.0416	1.4998	-1.2042	-1.1817	0.4263	0.3885
**7**	0.0115	-0.0328	0.4360	-1.1895	0.5378	-0.0374	-0.0032	-0.0005	0.0401	0.0395	1.5007	-1.2056	-1.1819	0.4258	0.3892
**8**	0.0118	-0.0326	0.4383	-1.1893	0.5378	-0.0370	-0.0032	-0.0005	0.0369	0.0392	1.4998	-1.2029	-1.1811	0.4253	0.3891
**9**	0.0118	-0.0345	0.4374	-1.1872	0.5376	-0.0369	-0.0032	-0.0029	0.0439	0.0441	1.4946	-1.2104	-1.1846	0.4270	0.3875
**10**	0.0119	-0.0341	0.4388	-1.1872	0.5373	-0.0365	-0.0034	-0.0028	0.0397	0.0438	1.4935	-1.2100	-1.1844	0.4266	0.3877
**11**	0.0113	-0.0344	0.4376	-1.1868	0.5376	-0.0375	-0.0032	-0.0033	0.0469	0.0443	1.4944	-1.2107	-1.1840	0.4274	0.3868
**12**	0.0114	-0.0331	0.4365	-1.1886	0.5378	-0.0374	-0.0031	-0.0025	0.0512	0.0375	1.5027	-1.2110	-1.1898	0.4336	0.3921
**13**	0.0114	-0.0330	0.4376	-1.1889	0.5377	-0.0368	-0.0033	-0.0026	0.0467	0.0367	1.5029	-1.2104	-1.1893	0.4327	0.3925

Table S5. Electron density and its Laplacian at selected Bond Critical Points (BCPs) derived from the QTAIM theory. For details see Scheme S1. The data is given for gas phase results.

Compound	Bond	Electron density (ρ= a.u.)	Laplacian ($\nabla^2{}_\rho$= a.u.)
		in vacuo	
4	**C1-C2**	0.3142	-0.9073
	**C2-C3**	0.3125	-0.8920
	**C3-N1**	0.3611	-1.2294
	**N1-C4**	0.3504	-1.1473
	**C4-C5**	0.3245	-0.9721
	**C5-C1**	0.3223	-0.9582
5	**C1-C2**	0.3142	-0.9073
	**C2-C3**	0.3126	-0.8926
	**C3-N1**	0.3611	-1.2295
	**N1-C4**	0.3503	-1.1466
	**C4-C5**	0.3245	-0.9720
	**C5-C1**	0.3223	-0.9585

Table S5 (Continuation). Electron density and its Laplacian at selected Bond Critical Points (BCPs) derived from the QTAIM theory. For details see Scheme S1.

Compound	Bond	Electron density (ρ= a.u.)	Laplacian ($\nabla^2{}_{\rho}$= a.u.)
		in vacuo	
**6**	**C1-C2**	0.3141	-0.9072
	**C2-C3**	0.3124	-0.8918
	**C3-N1**	0.3610	-1.2292
	**N1-C4**	0.3505	-1.1477
	**C4-C5**	0.3245	-0.9722
	**C5-C1**	0.3223	-0.9582
**7**	**C1-C2**	0.3141	-0.9073
	**C2-C3**	0.3127	-0.8929
	**C3-N1**	0.3611	-1.2296
	**N1-C4**	0.3503	-1.1463
	**C4-C5**	0.3245	-0.9719
	**C5-C1**	0.3223	-0.9586

Table S5 (Continuation). Electron density and its Laplacian at selected Bond Critical Points (BCPs) derived from the QTAIM theory. For details see Scheme S1.

**Compound**	**Bond**	**Electron density (ρ= a.u.)**	**Laplacian (∇^2_ρ= a.u.)**
		in vacuo	
**8**	**C1-C2**	0.3141	-0.9074
	**C2-C3**	0.3128	-0.8935
	**C3-N1**	0.3614	-1.2309
	**N1-C4**	0.3501	-1.1451
	**C4-C5**	0.3244	-0.9716
	**C5-C1**	0.3224	-0.9587
**9**	**C1-C2**	0.3151	-0.9116
	**C2-C3**	0.3131	-0.8947
	**C3-N1**	0.3615	-1.2321
	**N1-C4**	0.3500	-1.1459
	**C4-C5**	0.3240	-0.9693
	**C5-C1**	0.3222	-0.9579

Table S5 (Continuation). Electron density and its Laplacian at selected Bond Critical Points (BCPs) derived from the QTAIM theory. For details see Scheme S1.

Compound	Bond	Electron density (ρ= a.u.)	Laplacian ($\nabla^2{}_\rho$= a.u.)
		in vacuo	
10	C1-C2	0.3150	-0.9113
	C2-C3	0.3132	-0.8952
	C3-N1	0.3618	-1.2334
	N1-C4	0.3498	-1.1447
	C4-C5	0.3240	-0.9692
	C5-C1	0.3223	-0.9582
11	C1-C2	0.3153	-0.9123
	C2-C3	0.3131	-0.8945
	C3-N1	0.3614	-1.2317
	N1-C4	0.3500	-1.1463
	C4-C5	0.3240	-0.9690
	C5-C1	0.3222	-0.9576

Table S5 (Continuation). Electron density and its Laplacian at selected Bond Critical Points (BCPs) derived from the QTAIM theory. For details see Scheme S1.

Compound	Bond	Electron density (ρ= a.u.)	Laplacian (∇^2_{ρ}= a.u.)
		in vacuo	
**12**	**C1-C2**	0.3150	-0.9112
	**C2-C3**	0.3131	-0.8949
	**C3-N1**	0.3612	-1.2301
	**N1-C4**	0.3501	-1.1459
	**C4-C5**	0.3242	-0.9699
	**C5-C1**	0.3221	-0.9575
**13**	**C1-C2**	0.3149	-0.9107
	**C2-C3**	0.3133	-0.8958
	**C3-N1**	0.3615	-1.2315
	**N1-C4**	0.3499	-1.1444
	**C4-C5**	0.3241	-0.9696
	**C5-C1**	0.3222	-0.9579

Compound 4

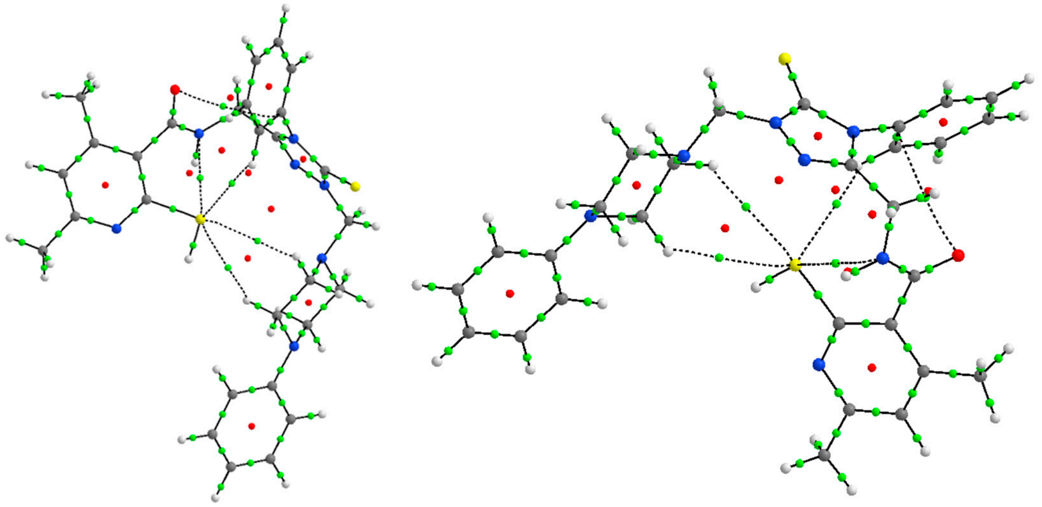

Compound 5

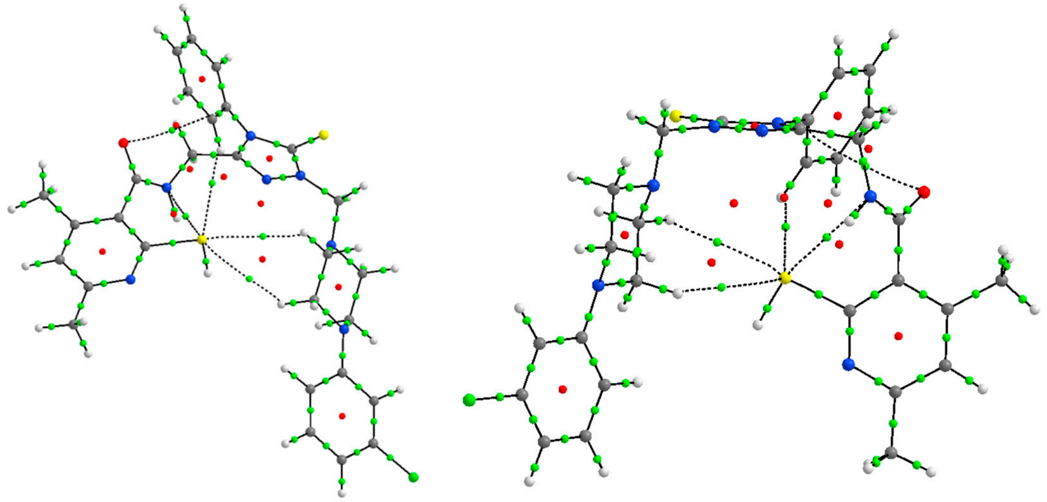

Figure S2. QTAIM molecular graphs of the *N*-Mannich bases obtained as a result of DFT/ωB97XD/def2-TZVP simulations *in vacuo* (two presentations to show the detected network of noncovalent interactions). Color coding: carbon—grey, oxygen—red, nitrogen—blue, sulphur—yellow, hydrogen—white, chlorine—green, fluorine—green (compound 7). Dotted lines indicate detected noncovalent interactions. Green and red spheres indicate the presence of Bond and Ring Critical Points (BCPs and RCPs), respectively.

Compound 6

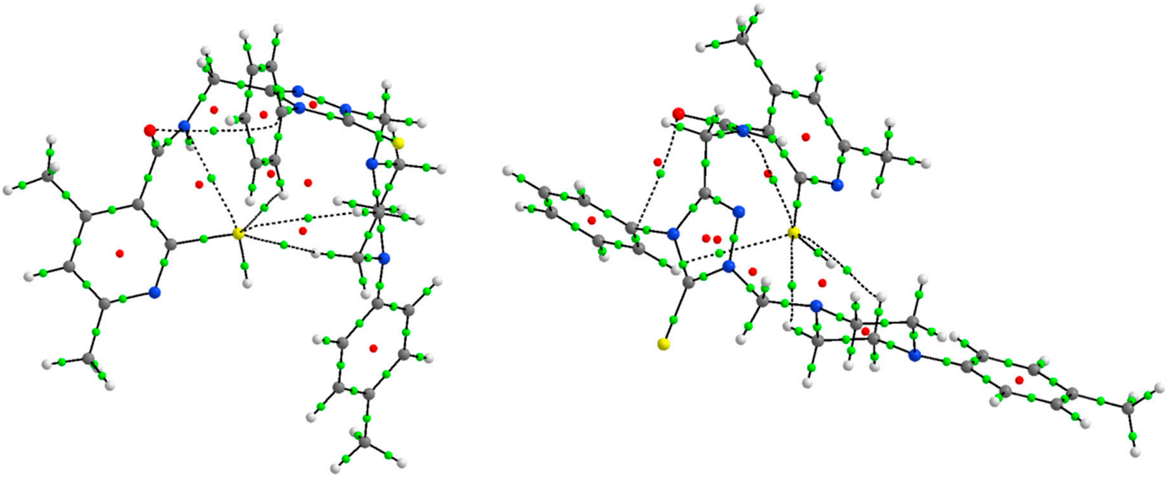

Compound 7

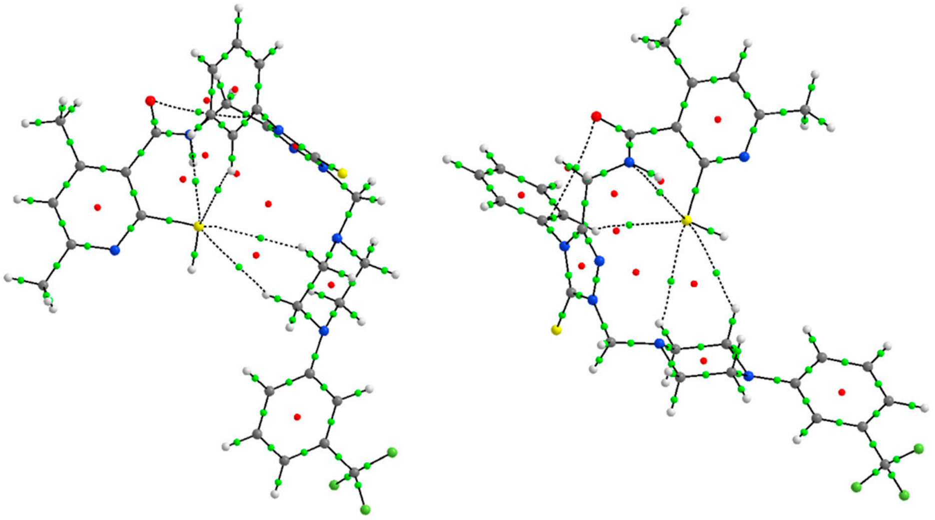

Figure S2 (Continuation). QTAIM molecular graphs of the *N*-Mannich bases obtained as a result of DFT/ωB97XD/def2-TZVP simulations *in vacuo* (two presentations to show the detected network of noncovalent interactions). Color coding: carbon—grey, oxygen—red, nitrogen—blue, sulphur—yellow, hydrogen—white, chlorine—green, fluorine—green (compound 7). Dotted lines indicate detected noncovalent interactions. Green and red spheres indicate the presence of Bond and Ring Critical Points (BCPs and RCPs), respectively.

Compound 8

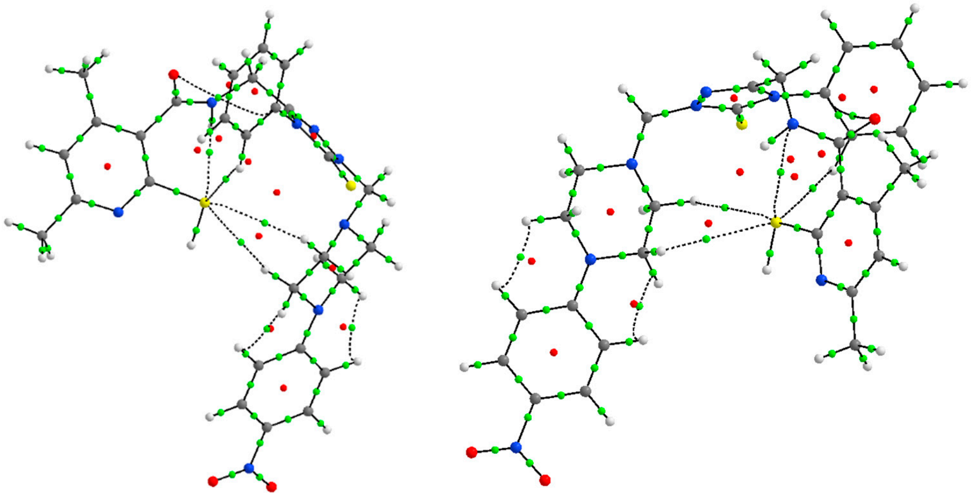

Compound 9

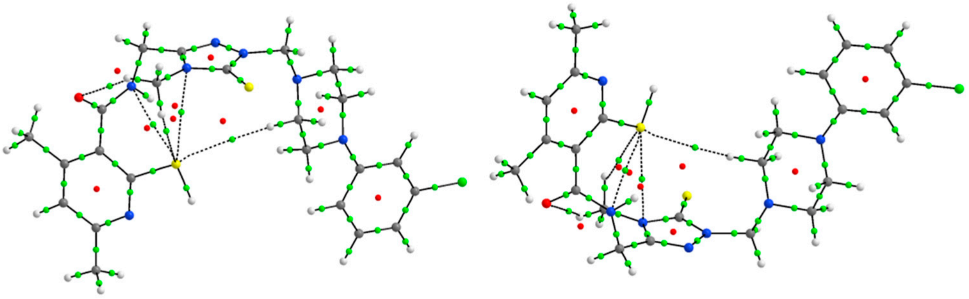

Figure S2 (Continuation). QTAIM molecular graphs of the *N*-Mannich bases obtained as a result of DFT/ωB97XD/def2-TZVP simulations *in vacuo* (two presentations to show the detected network of noncovalent interactions). Color coding: carbon—grey, oxygen—red, nitrogen—blue, sulphur—yellow, hydrogen—white, chlorine—green, fluorine—green (compound 7). Dotted lines indicate detected noncovalent interactions. Green and red spheres indicate the presence of Bond and Ring Critical Points (BCPs and RCPs), respectively.

Compound 10

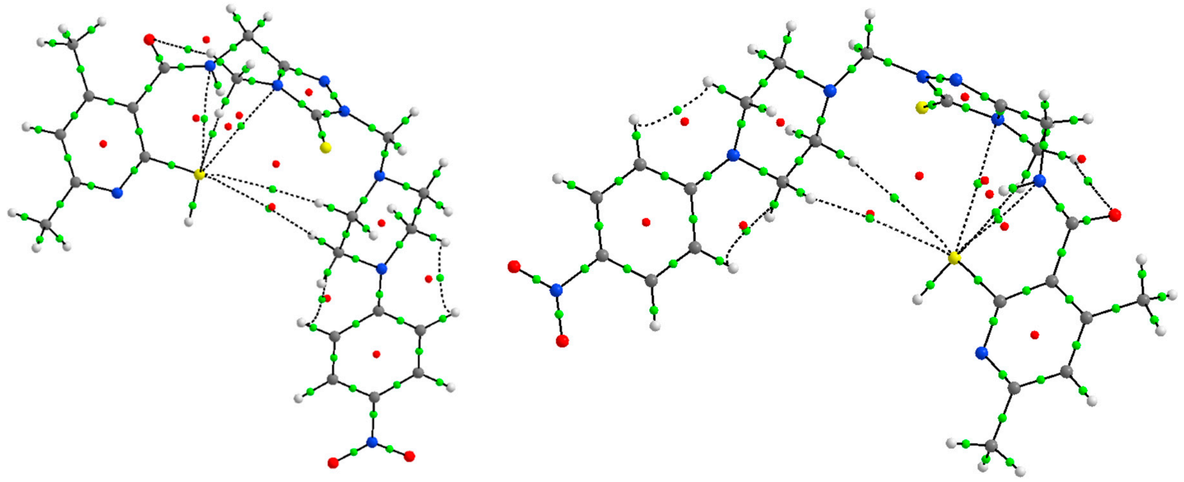

Compound 11

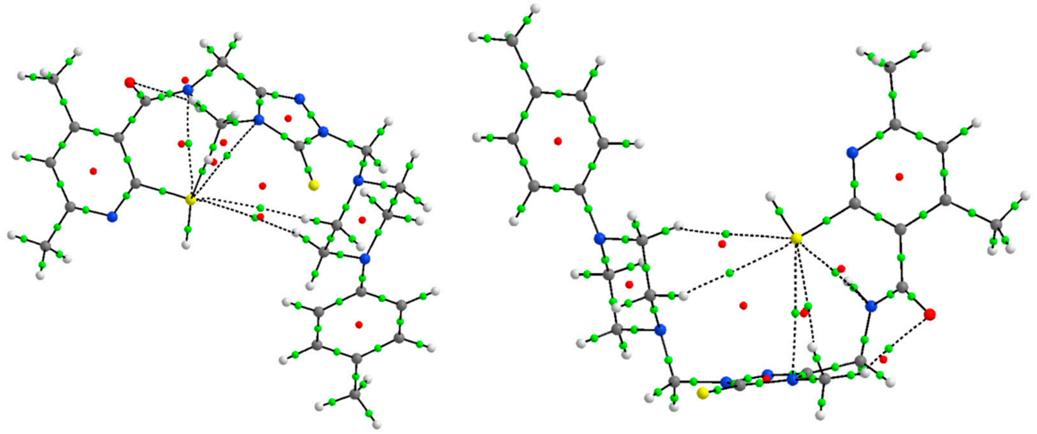

Figure S2 (Continuation). QTAIM molecular graphs of the *N*-Mannich bases obtained as a result of DFT/ωB97XD/def2-TZVP simulations *in vacuo* (two presentations to show the detected network of noncovalent interactions). Color coding: carbon—grey, oxygen—red, nitrogen—blue, sulphur—yellow, hydrogen—white, chlorine—green, fluorine—green (compound 7). Dotted lines indicate detected noncovalent interactions. Green and red spheres indicate the presence of Bond and Ring Critical Points (BCPs and RCPs), respectively.

Compound 12

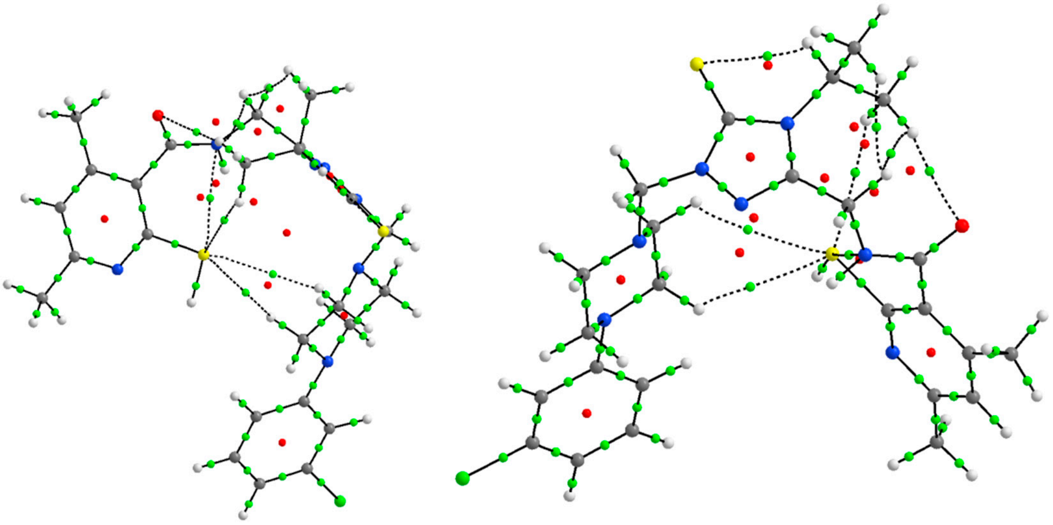

Compound 13

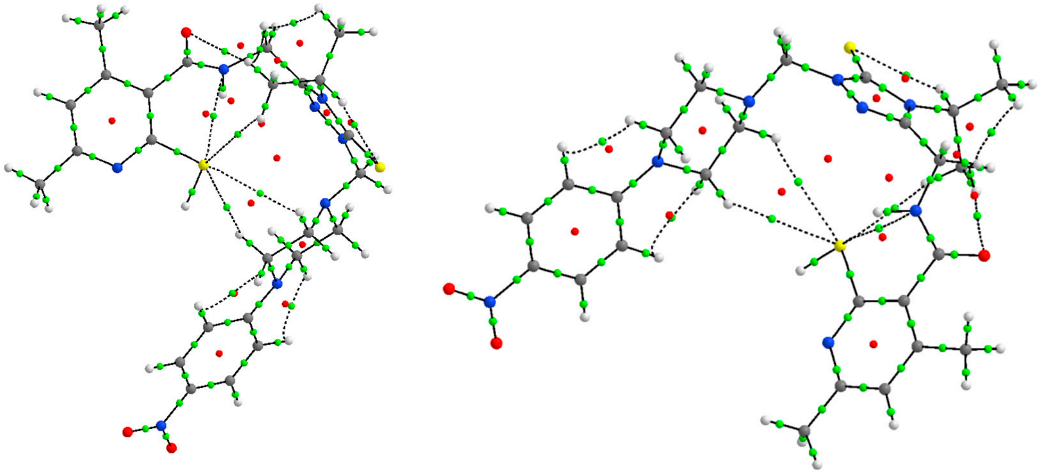

Figure S2 (Continuation). QTAIM molecular graphs of the *N*-Mannich bases obtained as a result of DFT/ωB97XD/def2-TZVP simulations *in vacuo* (two presentations to show the detected network of noncovalent interactions). Color coding: carbon—grey, oxygen—red, nitrogen—blue, sulphur—yellow, hydrogen—white, chlorine—green, fluorine—green (compound 7). Dotted lines indicate detected noncovalent interactions. Green and red spheres indicate the presence of Bond and Ring Critical Points (BCPs and RCPs), respectively.

Compound 4

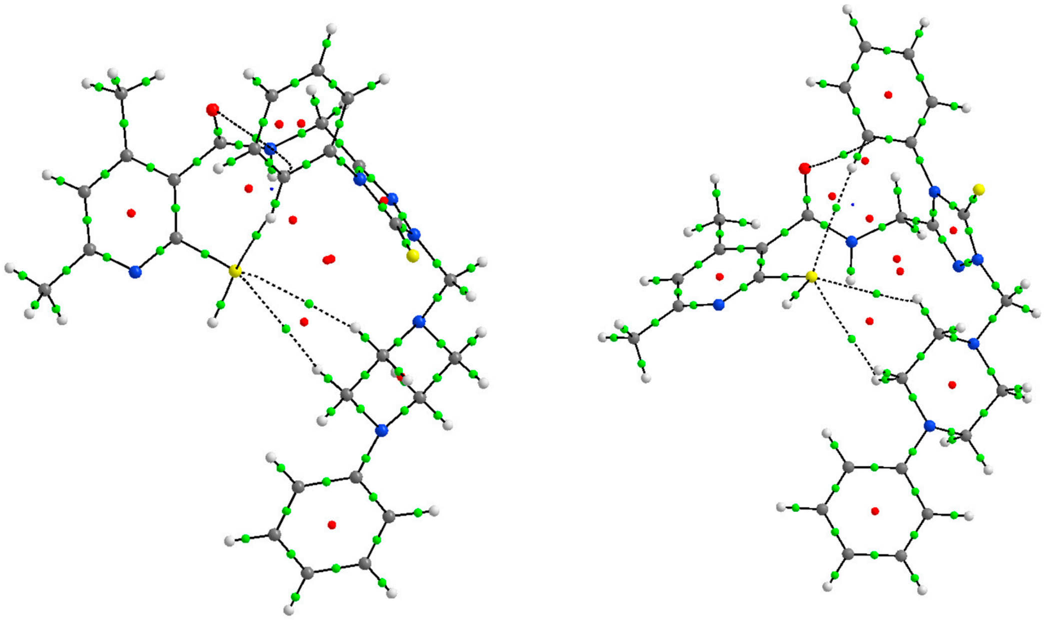

Compound 5

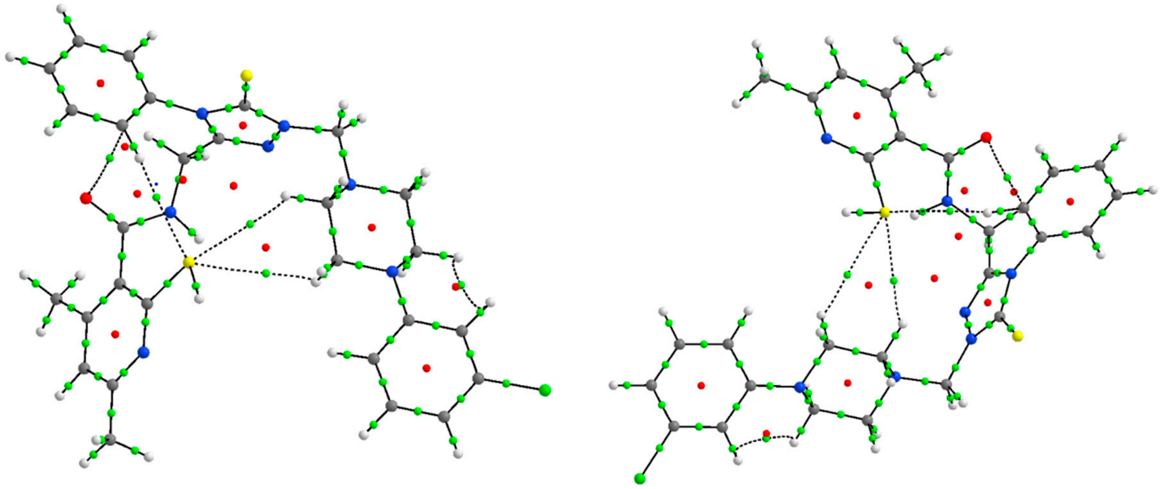

Figure S3. QTAIM molecular graphs of the *N*-Mannich bases obtained as a result of DFT/ωB97XD/def2-TZVP simulations with the PCM solvation model (two presentations to show the detected network of noncovalent interactions). Color coding: carbon—grey, oxygen—red, nitrogen—blue, sulphur—yellow, hydrogen—white, chlorine—green, fluorine—green (compound 7). Dotted lines indicate detected noncovalent interactions. Green and red spheres indicate the presence of Bond and Ring Critical Points (BCPs and RCPs), respectively.

Compound 6

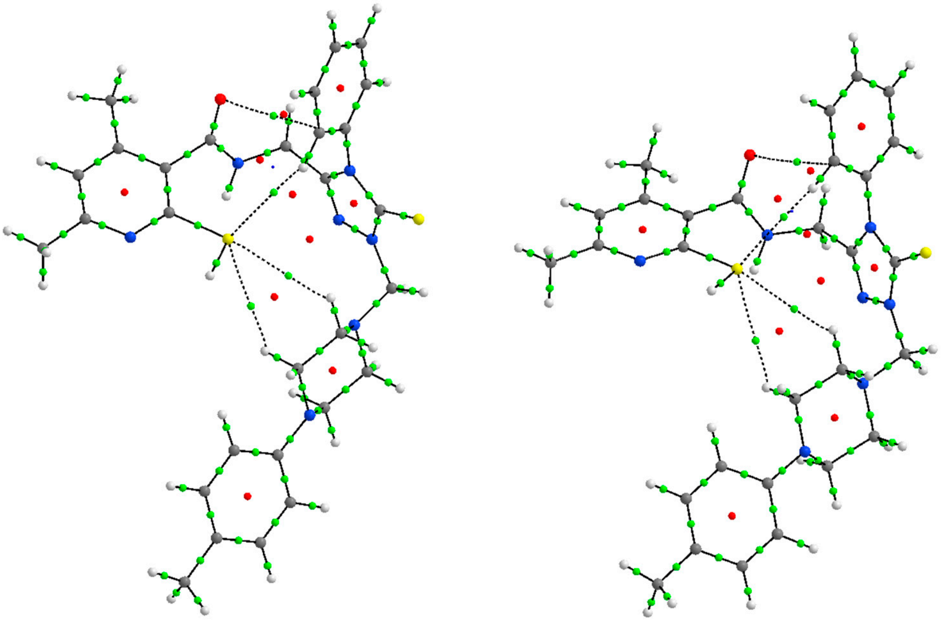

Compound 7

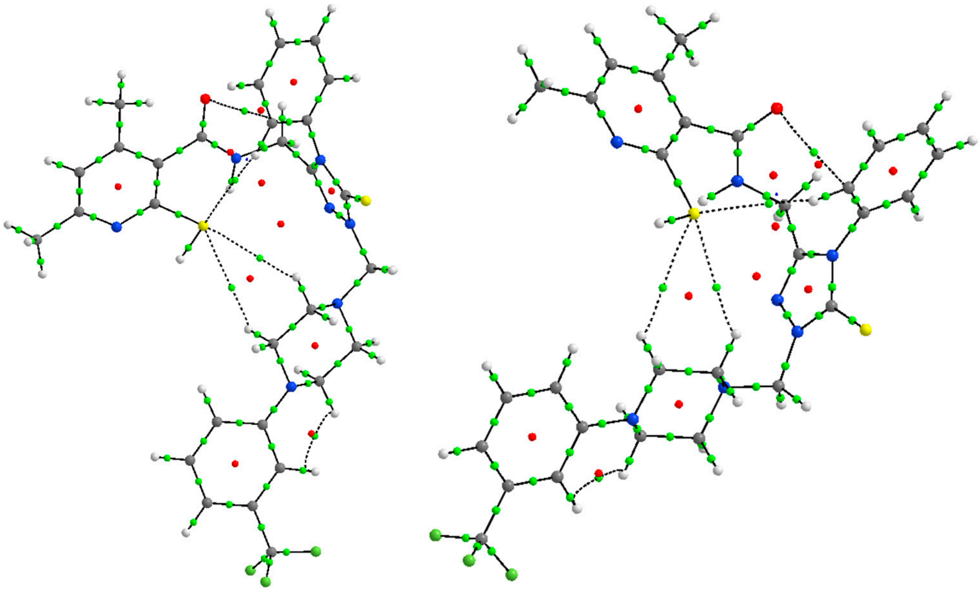

Figure S3 (Continuation). QTAIM molecular graphs of the *N*-Mannich bases obtained as a result of DFT/ωB97XD/def2-TZVP simulations with the PCM solvation model (two presentations to show the detected network of noncovalent interactions). Color coding: carbon—grey, oxygen—red, nitrogen—blue, sulphur—yellow, hydrogen—white, chlorine—green, fluorine—green (compound 7). Dotted lines indicate detected noncovalent interactions. Green and red spheres indicate the presence of Bond and Ring Critical Points (BCPs and RCPs), respectively.

Compound 8

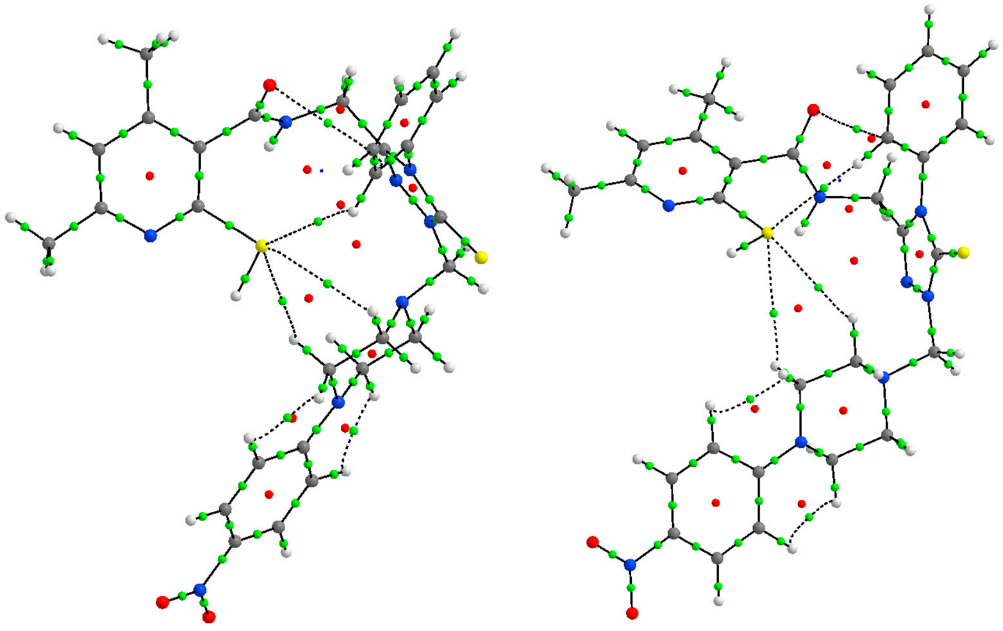

Compound 9

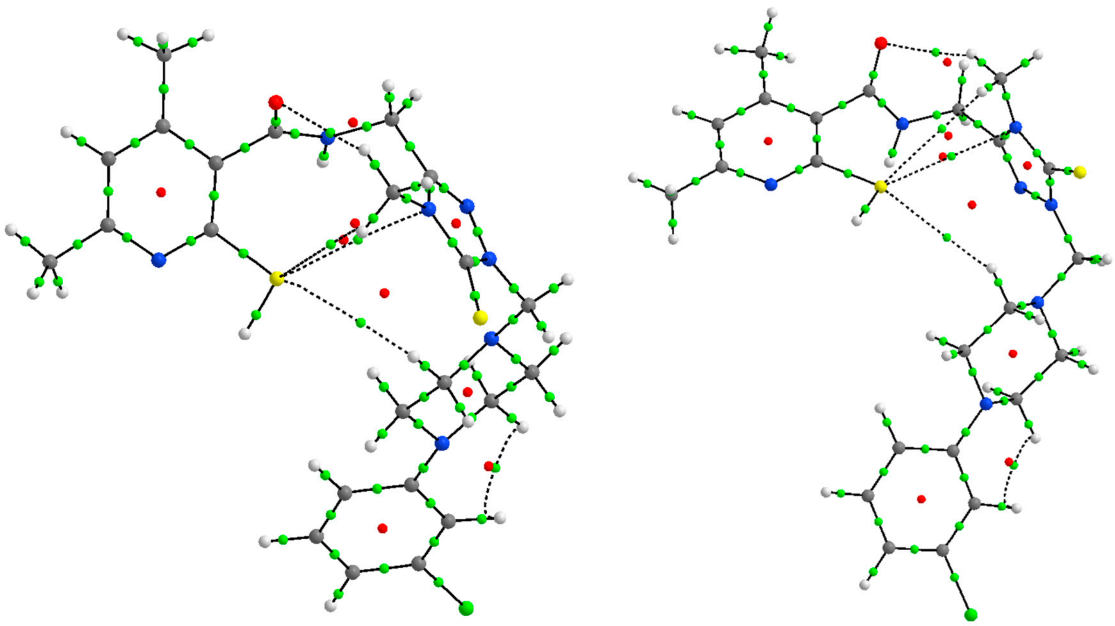

Figure S3 (Continuation). QTAIM molecular graphs of the *N*-Mannich bases obtained as a result of DFT/ωB97XD/def2-TZVP simulations with the PCM solvation model (two presentations to show the detected network of noncovalent interactions). Color coding: carbon—grey, oxygen—red, nitrogen—blue, sulphur—yellow, hydrogen—white, chlorine—green, fluorine—green (compound 7). Dotted lines indicate detected noncovalent interactions. Green and red spheres indicate the presence of Bond and Ring Critical Points (BCPs and RCPs), respectively.

Compound 10

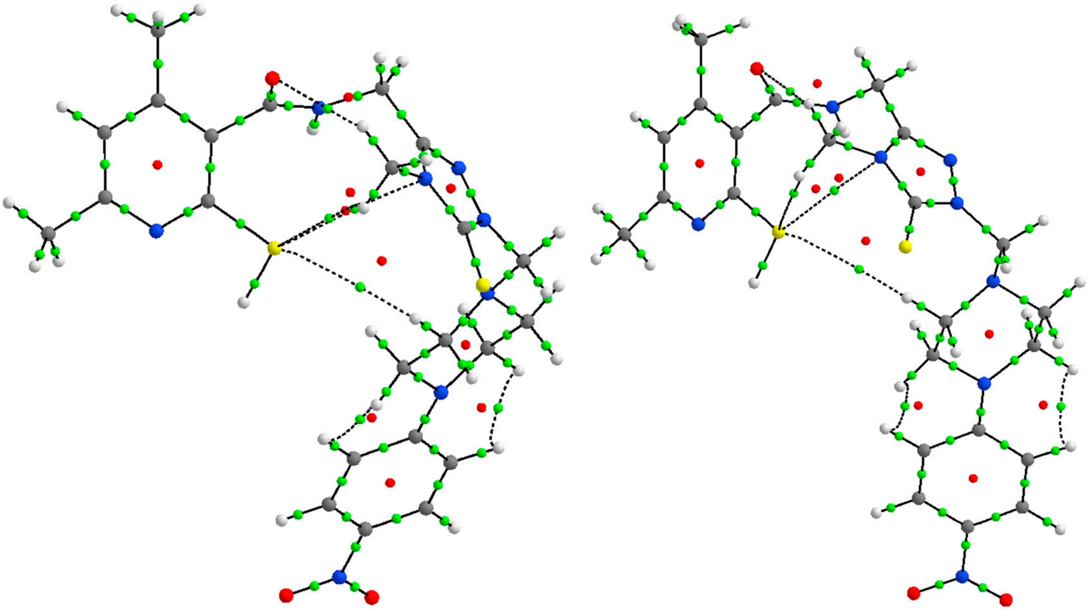

Compound 11

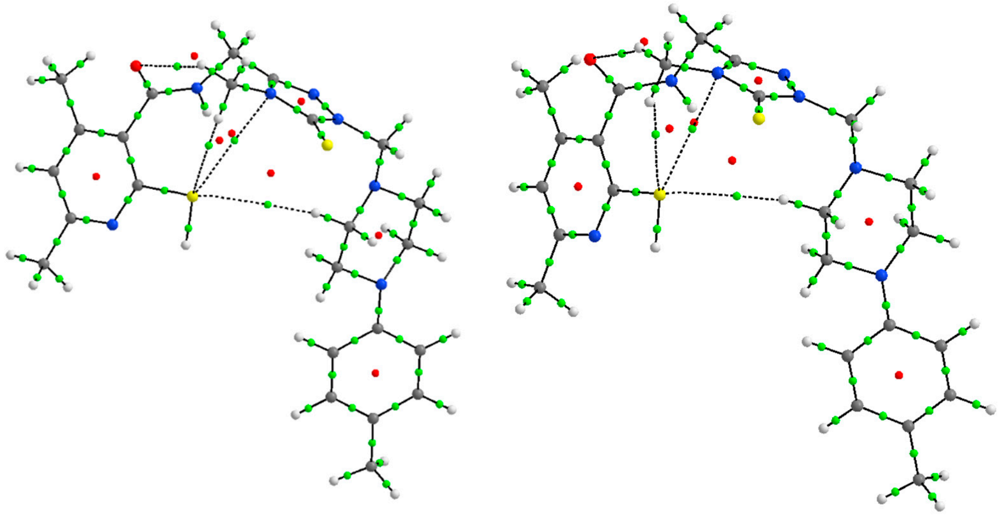

Figure S3 (Continuation). QTAIM molecular graphs of the *N*-Mannich bases obtained as a result of DFT/ωB97XD/def2-TZVP simulations with the PCM solvation model (two presentations to show the detected network of noncovalent interactions). Color coding: carbon—grey, oxygen—red, nitrogen—blue, sulphur—yellow, hydrogen—white, chlorine—green, fluorine—green (compound 7). Dotted lines indicate detected noncovalent interactions. Green and red spheres indicate the presence of Bond and Ring Critical Points (BCPs and RCPs), respectively.

Compound 12

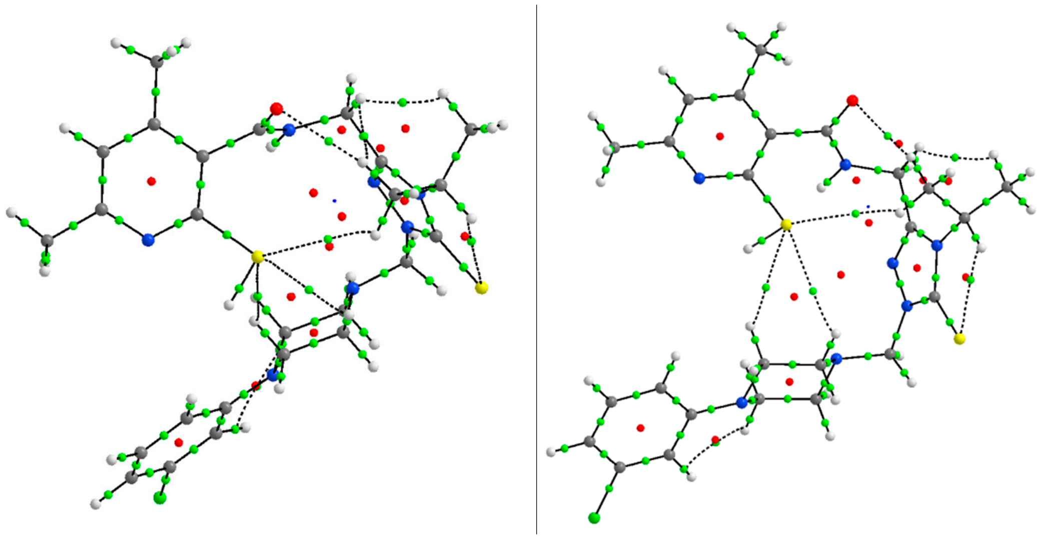

Compound 13

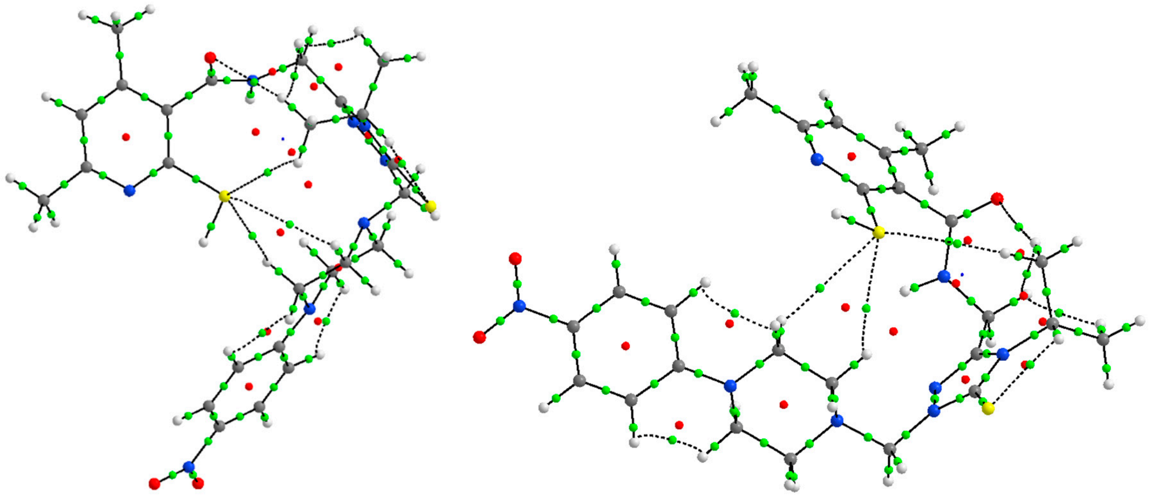

Figure S3 (Continuation). QTAIM molecular graphs of the *N*-Mannich bases obtained as a result of DFT/ωB97XD/def2-TZVP simulations with the PCM solvation model (two presentations to show the detected network of noncovalent interactions). Color coding: carbon—grey, oxygen—red, nitrogen—blue, sulphur—yellow, hydrogen—white, chlorine—green, fluorine—green (compound 7). Dotted lines indicate detected noncovalent interactions. Green and red spheres indicate the presence of Bond and Ring Critical Points (BCPs and RCPs), respectively.

Table S6. Binding affinity (kcal/mol) and molecular volume (Å) values from the molecular docking experiments for native ligands (YIN and NA3) as well as the synthesized set of *N*-Mannich bases.

**Compounds**	**Volume**	**MDM-2**		**CASPASE-3**
		**Binding affinity**		**Binding affinity**
		**Raw model**	**Modelled**	
**NA3**	226	-		-7.5
**YIN**	412	-9.3	-9.3	-
**4**	545	-8.8	-9.2	-8.5
**5**	562	-9.1	-9.4	-8.6
**6**	564	-8.8	-9.2	-8.5
**7**	577	-8.7	-8.8	-8.4
**8**	567	-10.0	-9.1	-9.5
**9**	497	-8.1	-8.2	-7.6
**10**	503	-8.8	-8.4	-9.3
**11**	499	-7.8	-7.9	-7.2
**12**	537	-8.7	-9.1	-7.8
**13**	541	-8.9	-8.4	-8.8

XYZ coordinates

XYZ coordinates of 10 *N*-Mannich bases (simulations *in vacuo*)

Compound 4

C	-2.750409	4.854860	0.270129
C	-3.276874	3.678230	0.782213
C	-2.564156	2.495951	0.566526
C	-1.563584	4.836915	-0.446020
N	-0.893756	3.706307	-0.660103
C	-1.375812	2.576376	-0.167176
C	-0.963751	6.083839	-1.020971
C	-4.570543	3.698303	1.541748
S	-0.414222	1.123281	-0.521184
C	-3.125853	1.193819	1.062148
O	-4.255134	0.838547	0.798578
N	-2.284928	0.466980	1.846776
C	-2.556685	-0.906665	2.216104
C	-1.662420	-1.829002	1.455132
N	-0.482087	-2.156491	1.856495
N	0.056711	-2.858821	0.823170
C	-0.782135	-2.976239	-0.237341
S	-0.536530	-3.741551	-1.687584
N	-1.906978	-2.302373	0.189923
C	-3.115490	-2.142638	-0.550096
C	-4.259184	-2.793905	-0.121429
C	-3.132208	-1.326211	-1.667138
C	-5.446248	-2.605047	-0.809550
C	-5.476449	-1.782956	-1.924683
C	-4.320052	-1.150050	-2.354453
C	1.426664	-3.307195	0.905617
N	2.340127	-2.199560	0.798971
C	2.438860	-1.662541	-0.546847
C	3.657021	-2.500046	1.323251
C	3.275678	-0.397931	-0.547889
C	4.505946	-1.243349	1.334327
N	4.590230	-0.660662	0.002099
C	5.585192	0.310956	-0.194444
C	5.309119	1.565470	-0.740855
C	6.915022	0.017402	0.130650
C	6.326551	2.484714	-0.958102
C	7.635055	2.186995	-0.625227
C	7.918814	0.942688	-0.076834
H	-1.567531	6.961699	-0.795317
H	-0.871521	5.987141	-2.103697
H	0.040498	6.232637	-0.621666
H	-4.858015	4.721402	1.779881
H	-4.497155	3.130162	2.469748
H	-5.360773	3.233910	0.952090
H	-3.269666	5.791341	0.428657
H	-1.346816	0.802419	1.991707
H	-4.214760	-3.445451	0.742229
H	-6.345882	-3.105456	-0.476200
H	-6.404587	-1.635697	-2.461634
H	-4.342698	-0.509505	-3.226122
H	-2.222303	-0.836923	-1.985693
H	-2.370990	-1.052837	3.279449
H	-3.606954	-1.097936	2.005373
H	1.561122	-3.779346	1.878804
H	1.547227	-4.058952	0.115103
H	1.439128	-1.433189	-0.917882
H	2.881561	-2.397696	-1.238132
H	3.557231	-2.868900	2.346454
H	4.169306	-3.275405	0.727754
H	4.063669	-0.523298	2.040363
H	5.502306	-1.491450	1.692442

H	2.743213	0.381558	0.021840
H	3.385078	-0.055612	-1.577447
H	4.295173	1.842425	-0.991537
H	7.167400	-0.959875	0.520786
H	8.938822	0.682850	0.178402
H	8.424700	2.907376	-0.793233
H	6.081439	3.449671	-1.384756
H	0.487242	1.802768	-1.240160

Compound 5

C	-3.141127	4.903939	0.288152
C	-3.689076	3.738963	0.804122
C	-3.005798	2.541041	0.579422
C	-1.962504	4.859986	-0.440444
N	-1.320932	3.714619	-0.662911
C	-1.823820	2.595429	-0.166422
C	-1.340512	6.093792	-1.020065
C	-4.974102	3.786493	1.576884
S	-0.899605	1.120896	-0.532872
C	-3.592342	1.251133	1.078076
O	-4.731006	0.921032	0.822730
N	-2.761451	0.504981	1.855291
C	-3.061747	-0.861819	2.227643
C	-2.194226	-1.804650	1.460846
N	-1.017746	-2.156680	1.852774
N	-0.503511	-2.872955	0.816287
C	-1.354147	-2.974389	-0.236662
S	-1.137210	-3.747397	-1.687237
N	-2.460175	-2.275608	0.199061
C	-3.671782	-2.091592	-0.530449
C	-4.824644	-2.719144	-0.091101
C	-3.681674	-1.275507	-1.647794
C	-6.013653	-2.506349	-0.768796
C	-6.036935	-1.684334	-1.884159
C	-4.871740	-1.075397	-2.324653
C	0.855693	-3.352963	0.888280
N	1.794297	-2.267425	0.765612
C	1.891207	-1.740440	-0.583830
C	3.109902	-2.596706	1.274642
C	2.752401	-0.492506	-0.601192
C	3.985318	-1.358452	1.276907
N	4.065891	-0.773676	-0.055406
C	5.079726	0.164843	-0.277463
C	4.837200	1.403780	-0.875837
C	6.398792	-0.148687	0.073596
C	5.878496	2.287176	-1.116640
C	7.179353	1.982482	-0.762192
C	7.413326	0.753691	-0.164653
H	-1.922656	6.984576	-0.788388
H	-1.260258	5.996019	-2.103638
H	-0.329690	6.220204	-0.629683
H	-5.240016	4.815603	1.813964
H	-4.901181	3.220745	2.506460
H	-5.778837	3.334165	0.997561
H	-3.637179	5.851803	0.453113
H	-1.815827	0.820574	1.995557
H	-4.785898	-3.371032	0.772569
H	-6.920329	-2.988085	-0.427144
H	-6.966600	-1.518382	-2.412943
H	-4.889108	-0.434938	-3.196496
H	-2.764871	-0.805003	-1.974774
H	-2.872056	-1.011620	3.289776
H	-4.117544	-1.029252	2.024332
H	0.988944	-3.821878	1.863134
H	0.951422	-4.111997	0.101420

H	0.893461	-1.492371	-0.947745
H	2.312360	-2.488278	-1.274996
H	3.014989	-2.962756	2.299161
H	3.598574	-3.383055	0.673878
H	3.566840	-0.629894	1.987743
H	4.979194	-1.629489	1.624221
H	2.240443	0.303656	-0.036746
H	2.861660	-0.165214	-1.635379
H	3.833245	1.696071	-1.145262
H	6.641190	-1.110281	0.501970
H	7.992347	2.670920	-0.942041
H	5.664043	3.241711	-1.580639
H	0.003134	1.780254	-1.268870
Cl	9.039599	0.322429	0.281620

Compound 6

C	-2.559317	5.005888	0.300849
C	-3.196926	3.876236	0.792011
C	-2.585569	2.637627	0.580819
C	-1.363804	4.889192	-0.391139
N	-0.790779	3.705948	-0.601313
C	-1.379585	2.619003	-0.127997
C	-0.645573	6.082869	-0.942978
C	-4.499422	4.004094	1.525511
S	-0.540315	1.090346	-0.474685
C	-3.269480	1.386501	1.053715
O	-4.421855	1.136874	0.769486
N	-2.509998	0.578981	1.842538
C	-2.909607	-0.768706	2.190709
C	-2.089887	-1.759698	1.432035
N	-0.951079	-2.197504	1.847866
N	-0.461437	-2.935942	0.815158
C	-1.290406	-2.966428	-0.259194
S	-1.093668	-3.738874	-1.713085
N	-2.355691	-2.196384	0.157915
C	-3.529300	-1.913985	-0.601274
C	-4.738031	-2.456896	-0.201132
C	-3.446657	-1.087351	-1.707929
C	-5.888464	-2.146636	-0.907229
C	-5.818823	-1.312925	-2.011869
C	-4.599091	-0.789757	-2.413392
C	0.862630	-3.503695	0.911847
N	1.871239	-2.479906	0.827033
C	2.031856	-1.940489	-0.512217
C	3.150253	-2.902122	1.361831
C	2.980922	-0.758025	-0.491287
C	4.109442	-1.727694	1.391787
N	4.260667	-1.147457	0.064528
C	5.337110	-0.257113	-0.102393
C	5.179862	1.031150	-0.606810
C	6.636035	-0.673298	0.212350
C	6.278303	1.862152	-0.792916
C	7.562753	1.456251	-0.473412
C	7.715037	0.166839	0.035870
H	-1.175701	7.008456	-0.723044
H	-0.538355	5.985315	-2.024242
H	0.358821	6.141303	-0.521190
H	-4.703833	5.047259	1.762373
H	-4.493038	3.429247	2.452286
H	-5.313916	3.609462	0.918378
H	-2.998220	5.983015	0.456802
H	-1.547725	0.826511	2.005025
H	-4.772420	-3.119289	0.654678
H	-6.838129	-2.561474	-0.595892
H	-6.718397	-1.070619	-2.562775
H	-4.543963	-0.140291	-3.276996

H	-2.488101	-0.684566	-2.004347
H	-2.754449	-0.942592	3.254772
H	-3.969335	-0.862671	1.962376
H	0.941407	-3.993274	1.882517
H	0.928110	-4.257037	0.116365
H	1.060851	-1.616752	-0.889298
H	2.412239	-2.705990	-1.207564
H	3.006232	-3.268681	2.380623
H	3.596585	-3.715906	0.764530
H	3.727070	-0.975340	2.099507
H	5.076166	-2.067339	1.756901
H	2.516582	0.059456	0.085281
H	3.130504	-0.413083	-1.515082
H	4.197390	1.408766	-0.852440
H	6.802487	-1.680488	0.572229
H	6.116948	2.858110	-1.190296
H	0.433231	1.693967	-1.166887
C	8.752506	2.353988	-0.670110
H	8.452717	3.326787	-1.060465
H	9.282876	2.519364	0.270342
H	9.465346	1.917017	-1.373183
H	8.707504	-0.193166	0.284726

Compound 7

C	-3.539788	4.930862	0.288859
C	-4.101092	3.774577	0.809962
C	-3.436558	2.566662	0.582398
C	-2.366720	4.868986	-0.447425
N	-1.743143	3.714178	-0.672506
C	-2.258962	2.603083	-0.171187
C	-1.730875	6.093029	-1.032665
C	-5.380376	3.841051	1.590736
S	-1.358965	1.114582	-0.542189
C	-4.038528	1.285658	1.085539
O	-5.182521	0.970590	0.835289
N	-3.214342	0.529319	1.860049
C	-3.531109	-0.832955	2.235397
C	-2.679529	-1.787656	1.465434
N	-1.504718	-2.152348	1.850806
N	-1.005189	-2.876498	0.812481
C	-1.863476	-2.969904	-0.235095
S	-1.663871	-3.747451	-1.685672
N	-2.958683	-2.257922	0.206211
C	-4.172900	-2.061758	-0.515890
C	-5.329732	-2.675700	-0.067832
C	-4.180974	-1.247705	-1.634728
C	-6.520682	-2.451033	-0.738273
C	-6.542082	-1.630934	-1.855086
C	-5.373108	-1.035756	-2.304305
C	0.348507	-3.372251	0.876746
N	1.299165	-2.298017	0.744791
C	1.390369	-1.773289	-0.605820
C	2.615152	-2.643282	1.241987
C	2.265683	-0.535376	-0.631385
C	3.504926	-1.415391	1.237083
N	3.580427	-0.830801	-0.096121
C	4.601703	0.096875	-0.325576
C	4.369915	1.337959	-0.919626
C	5.922210	-0.229202	0.011723
C	5.417614	2.213261	-1.172223
C	6.713433	1.888954	-0.829045
C	6.951083	0.655433	-0.231652
H	-2.299960	6.992055	-0.800435
H	-1.656714	5.991787	-2.116341
H	-0.716756	6.206877	-0.646943

H	-5.631402	4.874108	1.826793
H	-5.308791	3.276797	2.521347
H	-6.194543	3.397567	1.017763
H	-4.021082	5.885985	0.455612
H	-2.264434	0.833076	1.997233
H	-5.292625	-3.326397	0.796806
H	-7.430395	-2.922063	-0.389842
H	-7.473252	-1.455737	-2.378202
H	-5.389073	-0.396807	-3.177278
H	-3.261180	-0.787940	-1.968494
H	-3.338575	-0.984086	3.296828
H	-4.589922	-0.986713	2.037047
H	0.483031	-3.840062	1.851939
H	0.430235	-4.134299	0.091263
H	0.392572	-1.513539	-0.961289
H	1.796714	-2.526244	-1.300235
H	2.525251	-3.008519	2.267192
H	3.089220	-3.435076	0.636776
H	3.101119	-0.682181	1.951457
H	4.498580	-1.698338	1.575677
H	1.767535	0.267128	-0.063590
H	2.370111	-0.210376	-1.666944
H	3.366350	1.640797	-1.180233
H	6.147271	-1.195187	0.438651
H	7.528442	2.573779	-1.019889
H	5.206646	3.169051	-1.634695
H	-0.456189	1.759003	-1.291280
C	8.366931	0.296542	0.133148
F	9.173792	0.313738	-0.939622
F	8.469905	-0.920428	0.681501
F	8.883696	1.168571	1.014741

Compound 8

C	2.580440	5.120334	-0.410611
C	3.322808	4.035985	-0.853939
C	2.814293	2.756345	-0.616672
C	1.384032	4.922012	0.261492
N	0.909002	3.700147	0.495767
C	1.599012	2.655415	0.067379
C	0.555696	6.065067	0.763382
C	4.626844	4.251497	-1.563599
S	0.886011	1.069879	0.445625
C	3.612239	1.553552	-1.032198
O	4.767196	1.399571	-0.696718
N	2.948533	0.672873	-1.829642
C	3.463483	-0.646083	-2.132497
C	2.691488	-1.681393	-1.382864
N	1.600360	-2.205480	-1.826401
N	1.130548	-2.958923	-0.794777
C	1.923673	-2.911004	0.306331
S	1.727919	-3.665973	1.769113
N	2.945549	-2.076304	-0.092895
C	4.077618	-1.707896	0.693153
C	5.327706	-2.184232	0.337508
C	3.913247	-0.867870	1.780180
C	6.436304	-1.792970	1.069728
C	6.284579	-0.945448	2.155587
C	5.024739	-0.489142	2.512041
C	-0.137874	-3.632082	-0.924393
N	-1.229561	-2.691088	-0.886278
C	-1.468234	-2.140907	0.434907
C	-2.458433	-3.230822	-1.428140
C	-2.493472	-1.025606	0.368067
C	-3.500534	-2.134146	-1.532634
N	-3.725176	-1.490065	-0.244502

C	-4.882254	-0.749875	-0.064952
C	-4.881717	0.468017	0.633782
C	-6.115913	-1.218344	-0.553011
C	-6.049696	1.173221	0.843006
C	-7.242866	0.681739	0.349786
C	-7.279685	-0.514556	-0.349859
H	1.009859	7.025878	0.525592
H	0.434389	5.989707	1.844923
H	-0.440942	6.025670	0.321550
H	4.748145	5.300097	-1.831263
H	4.691346	3.649175	-2.470545
H	5.457186	3.948237	-0.926026
H	2.938063	6.126565	-0.587472
H	1.980472	0.847225	-2.043187
H	5.426733	-2.858657	-0.503789
H	7.417667	-2.155743	0.793776
H	7.151515	-0.640160	2.727070
H	4.905800	0.171380	3.360651
H	2.924416	-0.517289	2.041121
H	3.365698	-0.851909	-3.197689
H	4.517069	-0.654476	-1.860538
H	-0.147539	-4.137893	-1.889815
H	-0.170287	-4.377866	-0.120023
H	-0.536679	-1.741094	0.836519
H	-1.814666	-2.918973	1.134386
H	-2.267298	-3.625782	-2.428034
H	-2.856461	-4.051947	-0.807055
H	-3.159140	-1.390680	-2.266849
H	-4.425666	-2.566919	-1.901712
H	-2.068884	-0.174716	-0.186066
H	-2.703275	-0.704001	1.387450
H	-3.959312	0.889255	1.002545
H	-6.173076	-2.164947	-1.069710
H	-8.224366	-0.888186	-0.717251
H	-6.039717	2.113160	1.375626
H	-0.130474	1.608816	1.129617
N	-8.476253	1.429932	0.565034
O	-9.502991	0.975893	0.100965
O	-8.409156	2.465919	1.195816

Compound 9

C	4.877080	3.863298	-0.270907
C	5.115363	2.577363	-0.732610
C	4.218202	1.576591	-0.357727
C	3.788939	4.120553	0.549814
N	2.944114	3.157926	0.915898
C	3.151386	1.927920	0.474570
C	3.499294	5.493437	1.075421
C	6.302077	2.283189	-1.601672
S	1.998093	0.697224	1.033709
C	4.457126	0.158718	-0.785631
O	5.477017	-0.440093	-0.505139
N	3.453778	-0.392241	-1.516022
C	3.389646	-1.806165	-1.825450
C	2.348772	-2.479455	-0.995149
N	1.149298	-2.694194	-1.423593
N	0.483430	-3.199642	-0.352004
C	1.264060	-3.296098	0.753482
S	0.881177	-3.831956	2.278579
N	2.479153	-2.831068	0.320375
C	-0.929513	-3.473963	-0.449454
N	-1.692562	-2.253465	-0.509956
C	-1.738702	-1.542766	0.755944
C	-3.031964	-2.457306	-1.023404
C	-2.413546	-0.195912	0.582487
C	-3.719268	-1.119731	-1.216981

N	-3.742423	-0.360879	0.026381
C	-4.619920	0.726621	0.093438
C	-4.220224	1.986417	0.546600
C	-5.962075	0.553620	-0.267743
C	-5.133341	3.025900	0.638291
C	-6.456036	2.858672	0.273208
C	-6.845856	1.607901	-0.179819
H	4.227866	6.221656	0.722005
H	3.510188	5.483537	2.166256
H	2.502784	5.809362	0.763679
H	6.776981	3.205902	-1.931317
H	6.015818	1.705093	-2.481644
H	7.030278	1.684761	-1.053776
H	5.544576	4.668801	-0.548907
H	2.617841	0.148501	-1.665649
H	3.119145	-1.947068	-2.870932
H	4.380865	-2.224738	-1.661685
H	-1.095829	-4.035615	-1.368728
H	-1.175490	-4.105866	0.413897
H	-0.722754	-1.386774	1.119909
H	-2.274243	-2.127130	1.521464
H	-2.970740	-2.965254	-1.988302
H	-3.640902	-3.080942	-0.346319
H	-3.185519	-0.556292	-1.997395
H	-4.734129	-1.291974	-1.566507
H	-1.782049	0.443280	-0.054929
H	-2.495788	0.273742	1.562700
H	-3.191518	2.171352	0.817733
H	-6.327425	-0.411994	-0.585452
H	-7.168854	3.668039	0.337006
H	-4.797996	3.992932	0.991590
H	1.344587	1.554081	1.827194
C	3.643033	-2.727351	1.174533
H	4.480177	-2.333976	0.603276
H	3.423946	-2.055060	2.003352
H	3.883882	-3.709752	1.578355
Cl	-8.506521	1.348475	-0.632786

Compound 10

C	4.302266	4.407498	-0.288589
C	4.783092	3.171371	-0.693796
C	4.055337	2.039360	-0.324627
C	3.146049	4.492519	0.473022
N	2.464002	3.406726	0.834473
C	2.903715	2.221114	0.446154
C	2.595963	5.807034	0.935586
C	6.045325	3.065828	-1.497265
S	1.954402	0.822969	0.997628
C	4.556639	0.673910	-0.690780
O	5.648144	0.267977	-0.343268
N	3.700814	-0.062090	-1.446135
C	3.897777	-1.472420	-1.711388
C	2.948859	-2.295872	-0.906500
N	1.826197	-2.724515	-1.380379
N	1.205881	-3.312448	-0.323133
C	1.936900	-3.245967	0.818274
S	1.575566	-3.802380	2.341011
N	3.073619	-2.588909	0.423941
C	-0.132121	-3.827382	-0.471292
N	-1.089864	-2.756163	-0.592894
C	-1.304645	-2.035123	0.648302
C	-2.357135	-3.198384	-1.135190
C	-2.178626	-0.818396	0.412963
C	-3.247054	-2.001431	-1.407888
N	-3.435594	-1.195744	-0.208310
C	-4.498689	-0.309068	-0.162677

C	-4.373289	0.969097	0.404906
C	-5.762639	-0.678797	-0.658038
C	-5.452607	1.825980	0.483242
C	-6.679138	1.428306	-0.012889
C	-6.837679	0.175567	-0.585256
H	3.208145	6.641331	0.596309
H	2.545083	5.825917	2.025107
H	1.579821	5.938390	0.561033
H	6.367442	4.047627	-1.840546
H	5.909844	2.420073	-2.366235
H	6.838472	2.622449	-0.894787
H	4.832997	5.309628	-0.564408
H	2.794453	0.322755	-1.655516
H	3.708318	-1.684392	-2.762695
H	4.936961	-1.707924	-1.489692
H	-0.160154	-4.424728	-1.382582
H	-0.305746	-4.476395	0.396849
H	-0.345680	-1.705905	1.048657
H	-1.770202	-2.682261	1.409271
H	-2.181388	-3.723612	-2.076298
H	-2.878420	-3.889840	-0.450646
H	-2.787347	-1.391597	-2.198634
H	-4.205110	-2.356515	-1.775776
H	-1.630609	-0.089984	-0.203208
H	-2.383443	-0.363652	1.381191
H	-3.419691	1.315947	0.771823
H	-5.917339	-1.662710	-1.075558
H	-7.807623	-0.121683	-0.956595
H	-5.345502	2.810450	0.915127
H	1.136455	1.572808	1.745743
C	4.158861	-2.263450	1.325184
H	4.943669	-1.746432	0.778787
H	3.786905	-1.617246	2.119517
H	4.543795	-3.178668	1.772702
N	-7.818446	2.335710	0.063772
O	-7.647486	3.419155	0.585865
O	-8.876012	1.957992	-0.399504

Compound 11

C	4.176617	4.101569	-0.213919
C	4.552487	2.837620	-0.643855
C	3.734645	1.763113	-0.293510
C	3.032599	4.266958	0.552685
N	2.264376	3.234779	0.897370
C	2.603690	2.023806	0.485946
C	2.592477	5.613779	1.040017
C	5.799340	2.642774	-1.454783
S	1.546110	0.697390	1.012739
C	4.119478	0.368368	-0.690537
O	5.171036	-0.136759	-0.348908
N	3.209362	-0.272878	-1.467668
C	3.281107	-1.690946	-1.756925
C	2.264733	-2.438987	-0.960869
N	1.096417	-2.731352	-1.427327
N	0.431117	-3.282025	-0.378160
C	1.181069	-3.330633	0.751327
S	0.788451	-3.898137	2.262611
N	2.375890	-2.784033	0.358086
C	-0.965068	-3.622401	-0.515269
N	-1.779089	-2.437343	-0.593567
C	-1.897511	-1.736785	0.673808
C	-3.089033	-2.689601	-1.159749
C	-2.622110	-0.419134	0.480936
C	-3.823760	-1.378372	-1.362864
N	-3.925596	-0.641428	-0.111092
C	-4.835602	0.431195	-0.095245

C	-4.476662	1.719092	0.294122
C	-6.172823	0.212202	-0.446776
C	-5.419408	2.739512	0.335121
C	-6.740319	2.527585	-0.021136
C	-7.094349	1.237406	-0.414997
H	3.270890	6.401066	0.714380
H	2.543842	5.617114	2.129804
H	1.590308	5.834569	0.669819
H	6.207668	3.601208	-1.771454
H	5.604620	2.037331	-2.341464
H	6.550235	2.112484	-0.868657
H	4.779514	4.961871	-0.474849
H	2.336876	0.189713	-1.663676
H	3.069936	-1.866242	-2.810790
H	4.296199	-2.020508	-1.544172
H	-1.079350	-4.187794	-1.440170
H	-1.204282	-4.268548	0.339435
H	-0.901320	-1.541875	1.072171
H	-2.433969	-2.347431	1.417814
H	-2.969574	-3.183169	-2.126833
H	-3.696514	-3.345724	-0.512651
H	-3.285379	-0.783373	-2.117471
H	-4.818927	-1.585037	-1.750345
H	-1.996291	0.243169	-0.139759
H	-2.752732	0.051392	1.455996
H	-3.454801	1.947186	0.562207
H	-6.498261	-0.783553	-0.719155
H	-8.123077	1.027700	-0.687522
H	-5.103088	3.728982	0.646287
H	0.775555	1.497932	1.758377
C	3.498976	-2.598218	1.251790
H	4.317632	-2.125661	0.714818
H	3.196434	-1.960599	2.081713
H	3.809173	-3.563090	1.650733
C	-7.761329	3.630402	0.017496
H	-7.315886	4.569476	0.346608
H	-8.201897	3.796086	-0.968226
H	-8.577613	3.389487	0.702310

Compound 12

C	-2.315925	5.076693	0.372721
C	-3.152672	4.036626	0.749646
C	-2.834690	2.752467	0.305110
C	-1.214400	4.830343	-0.432979
N	-0.924710	3.605188	-0.867725
C	-1.708142	2.600658	-0.508675
C	-0.285082	5.924389	-0.862695
C	-4.356263	4.296917	1.606383
S	-1.265539	1.007392	-1.157329
C	-3.729749	1.599017	0.654514
O	-4.911303	1.583219	0.369231
N	-3.115647	0.590836	1.320288
C	-3.744651	-0.686413	1.585275
C	-2.891416	-1.778188	1.021366
N	-1.760782	-2.067744	1.574192
N	-1.183162	-2.968612	0.740343
C	-1.951729	-3.252261	-0.337579
S	-1.623896	-4.289042	-1.598761
N	-3.071394	-2.473112	-0.145367
C	0.154488	-3.438075	1.014345
N	1.114850	-2.380276	0.836984
C	1.319617	-2.013763	-0.552858
C	2.383582	-2.658111	1.478423
C	2.197193	-0.780760	-0.644821
C	3.276048	-1.434230	1.405721
N	3.463396	-1.005835	0.025474

C	4.497716	-0.094061	-0.217163
C	4.306658	1.081290	-0.947495
C	5.786420	-0.374530	0.253410
C	5.369592	1.935423	-1.199689
C	6.640276	1.664213	-0.727971
C	6.822549	0.498846	0.000478
H	-0.591025	6.891287	-0.465750
H	-0.256826	5.982159	-1.951692
H	0.728424	5.707050	-0.522388
H	-4.341723	5.315175	1.991912
H	-4.399778	3.605663	2.449421
H	-5.268779	4.147359	1.029103
H	-2.523915	6.084852	0.707458
H	-2.131151	0.662505	1.519691
H	-3.841740	-0.846817	2.659987
H	-4.739702	-0.657827	1.152694
H	0.186133	-3.767484	2.053231
H	0.313924	-4.299599	0.353566
H	0.355484	-1.798075	-1.014818
H	1.779396	-2.838898	-1.120314
H	2.207090	-2.903749	2.527866
H	2.902436	-3.512041	1.009787
H	2.818124	-0.626245	1.996452
H	4.237794	-1.670912	1.854375
H	1.654116	0.074864	-0.213451
H	2.387438	-0.571072	-1.697806
H	3.325731	1.348653	-1.311417
H	5.989690	-1.291044	0.788009
H	5.195631	2.840864	-1.767452
H	-0.254771	1.498913	-1.883999
C	-4.231355	-2.503664	-1.044976
H	-3.930292	-3.232278	-1.798527
C	-5.463675	-3.041907	-0.330527
H	-5.832437	-2.359279	0.436686
H	-6.265407	-3.182582	-1.055821
H	-5.250017	-4.005867	0.132248
C	-4.455182	-1.167262	-1.735551
H	-3.540517	-0.828983	-2.222128
H	-5.221583	-1.289958	-2.501816
H	-4.795203	-0.387356	-1.053412
H	7.469546	2.330454	-0.916965
Cl	8.410717	0.110345	0.597828

Compound 13

C	-1.587539	5.236031	0.459412
C	-2.618596	4.358106	0.759138
C	-2.507168	3.044084	0.301786
C	-0.498619	4.807894	-0.285341
N	-0.405050	3.556453	-0.732084
C	-1.376298	2.704573	-0.445934
C	0.635029	5.723858	-0.632463
C	-3.808951	4.818154	1.547590
S	-1.183867	1.066805	-1.108691
C	-3.613729	2.064804	0.565614
O	-4.757408	2.261938	0.204724
N	-3.233166	0.955421	1.245592
C	-4.094116	-0.192413	1.440633
C	-3.410261	-1.411737	0.907971
N	-2.388792	-1.903880	1.526335
N	-1.922973	-2.881917	0.709445
C	-2.653005	-3.010158	-0.423962
S	-2.421762	-4.069882	-1.687333
N	-3.629138	-2.048718	-0.285069
C	-0.714841	-3.588294	1.059195
N	0.430901	-2.718951	0.970031
C	0.786629	-2.370974	-0.392706

C	1.587336	-3.238229	1.668273
C	1.866742	-1.306920	-0.400011
C	2.682727	-2.190164	1.700499
N	3.027700	-1.739934	0.357621
C	4.227076	-1.069267	0.176483
C	4.328468	0.055869	-0.656756
C	5.401654	-1.521748	0.804477
C	5.538512	0.688653	-0.859543
C	6.671596	0.216084	-0.226113
C	6.607023	-0.889746	0.607687
H	0.485343	6.722170	-0.223946
H	0.735513	5.797133	-1.716267
H	1.572118	5.320466	-0.246184
H	-3.634943	5.808514	1.965350
H	-4.034318	4.130155	2.363824
H	-4.692722	4.851281	0.910225
H	-1.633248	6.260336	0.806332
H	-2.267497	0.850412	1.511070
H	-4.290779	-0.345059	2.502782
H	-5.036827	0.016816	0.944949
H	-0.813014	-3.929748	2.089984
H	-0.666677	-4.456431	0.389792
H	-0.091405	-1.981720	-0.908931
H	1.132189	-3.254541	-0.953524
H	1.307868	-3.481486	2.695493
H	1.976045	-4.156115	1.194231
H	2.338534	-1.337472	2.303070
H	3.555573	-2.613238	2.189265
H	1.452697	-0.370199	0.001602
H	2.162566	-1.136976	-1.434743
H	3.452776	0.464225	-1.137167
H	5.379502	-2.402410	1.429399
H	7.506684	-1.251338	1.083980
H	5.606365	1.558742	-1.496313
H	-0.071528	1.388871	-1.779905
C	-4.715396	-1.864509	-1.256335
H	-4.498495	-2.626437	-2.005705
C	-6.066766	-2.184755	-0.631877
H	-6.360145	-1.455236	0.124537
H	-6.831366	-2.175492	-1.408931
H	-6.055759	-3.175619	-0.176818
C	-4.655878	-0.502354	-1.929865
H	-3.667644	-0.327308	-2.354560
H	-5.383145	-0.478530	-2.742293
H	-4.893815	0.318366	-1.252400
N	7.949221	0.888861	-0.433777
O	7.972173	1.845922	-1.181526
O	8.920101	0.455227	0.153606

XYZ coordinates of 10 *N*-Mannich bases (simulations with application of PCM solvation model and water as a solvent)

Compound 4

C	-2.319375	5.100619	0.375999
C	-2.953884	3.946570	0.812737
C	-2.372691	2.722211	0.482280
C	-1.156253	5.017059	-0.374430
N	-0.608323	3.841827	-0.690935
C	-1.196113	2.732698	-0.270085
C	-0.446535	6.239807	-0.869819
C	-4.220246	4.025302	1.612436
S	-0.416258	1.203506	-0.731078
C	-3.040290	1.438524	0.883061
O	-4.136345	1.119868	0.455670
N	-2.329737	0.683933	1.749982
C	-2.699884	-0.662672	2.128349
C	-1.806745	-1.654900	1.456173
N	-0.637231	-1.976131	1.896605
N	-0.115071	-2.798936	0.948540
C	-0.955939	-3.004141	-0.084730
S	-0.741487	-3.956219	-1.446613
N	-2.054949	-2.253789	0.246835
C	-3.252443	-2.147023	-0.524838
C	-4.392216	-2.805018	-0.094671
C	-3.253210	-1.380651	-1.677725
C	-5.560417	-2.682901	-0.831566
C	-5.573805	-1.920783	-1.990625
C	-4.421110	-1.273608	-2.414079
C	1.240550	-3.284445	1.100863
N	2.186693	-2.211772	0.935853
C	2.307925	-1.766634	-0.442534
C	3.495100	-2.545840	1.472476
C	3.211404	-0.552632	-0.532747
C	4.409821	-1.339648	1.398296
N	4.516490	-0.846317	0.029125
C	5.554081	0.061677	-0.231937
C	5.341931	1.265352	-0.910806
C	6.865186	-0.244938	0.159002
C	6.401286	2.118212	-1.194132
C	7.690669	1.807300	-0.798894
C	7.910608	0.615868	-0.117185
H	-0.955176	7.150440	-0.558460
H	-0.389898	6.223657	-1.959431
H	0.575867	6.259405	-0.489175
H	-4.385625	5.039061	1.972420
H	-4.191420	3.350705	2.469140
H	-5.072904	3.732219	0.998463
H	-2.732553	6.070073	0.621934
H	-1.441624	1.030672	2.073542
H	-4.362498	-3.403569	0.806611
H	-6.457928	-3.188563	-0.500910
H	-6.486102	-1.829893	-2.565751
H	-4.432522	-0.676161	-3.316073
H	-2.349762	-0.870676	-1.984558
H	-2.602076	-0.783664	3.205435
H	-3.738978	-0.820530	1.850177
H	1.335819	-3.691059	2.106831
H	1.360093	-4.095555	0.372862
H	1.322075	-1.498816	-0.825166
H	2.706742	-2.570052	-1.083136
H	3.387639	-2.845334	2.516295
H	3.955479	-3.383628	0.923431
H	4.016210	-0.550128	2.056455
H	5.390076	-1.624762	1.770524
H	2.729009	0.293792	-0.018850

H	3.323059	-0.288944	-1.584076
H	4.347491	1.555892	-1.217354
H	7.073488	-1.180308	0.661146
H	8.912535	0.345680	0.193385
H	8.512394	2.476388	-1.018257
H	6.203441	3.042786	-1.722806
H	0.512793	1.791464	-1.494212

Compound 5

C	-2.765629	5.117043	0.395408
C	-3.404955	3.968474	0.839486
C	-2.841981	2.739110	0.496476
C	-1.615797	5.023397	-0.374099
N	-1.085217	3.843394	-0.702253
C	-1.677639	2.739334	-0.274642
C	-0.901668	6.240039	-0.878120
C	-4.657438	4.057613	1.659673
S	-0.921623	1.203125	-0.751915
C	-3.516537	1.461357	0.904513
O	-4.621351	1.153953	0.491612
N	-2.801451	0.698718	1.760529
C	-3.180526	-0.643844	2.144333
C	-2.304940	-1.645281	1.462760
N	-1.133610	-1.977363	1.890103
N	-0.630145	-2.805650	0.936667
C	-1.484602	-3.003341	-0.086995
S	-1.293880	-3.957476	-1.450859
N	-2.572622	-2.242480	0.256719
C	-3.777697	-2.124296	-0.501464
C	-4.919427	-2.769358	-0.057165
C	-3.783370	-1.359925	-1.655664
C	-6.094616	-2.635794	-0.780837
C	-6.113080	-1.875443	-1.940981
C	-4.958380	-1.241519	-2.378807
C	0.722354	-3.303609	1.073140
N	1.676260	-2.239208	0.896762
C	1.782370	-1.792201	-0.481905
C	2.989467	-2.587508	1.411171
C	2.689849	-0.582079	-0.581988
C	3.911793	-1.387429	1.334446
N	3.999894	-0.878698	-0.030917
C	5.040895	0.007633	-0.316827
C	4.842059	1.191241	-1.036437
C	6.347514	-0.300764	0.089054
C	5.909431	2.021766	-1.342805
C	7.198521	1.721836	-0.938669
C	7.386322	0.551238	-0.221025
H	-1.397140	7.155033	-0.558410
H	-0.860697	6.224786	-1.968438
H	0.126196	6.249523	-0.512038
H	-4.808111	5.072331	2.023370
H	-4.620581	3.381745	2.515088
H	-5.522456	3.772540	1.059371
H	-3.164651	6.090086	0.650373
H	-1.906299	1.037048	2.073433
H	-4.885760	-3.366916	0.844636
H	-6.993674	-3.131209	-0.439043
H	-7.030886	-1.775600	-2.505758
H	-4.973669	-0.645532	-3.281709
H	-2.878073	-0.860290	-1.973781
H	-3.071532	-0.765112	3.220323
H	-4.224286	-0.791487	1.878292
H	0.826308	-3.710898	2.077951
H	0.826063	-4.115662	0.343835
H	0.793825	-1.517581	-0.852265
H	2.168901	-2.596203	-1.129184

H	2.896167	-2.893948	2.454210
H	3.435429	-3.424294	0.849008
H	3.535396	-0.601579	2.005680
H	4.894449	-1.685422	1.689022
H	2.218036	0.267982	-0.065685
H	2.792554	-0.323448	-1.635124
H	3.852263	1.483585	-1.353141
H	6.559921	-1.217178	0.619677
H	8.029961	2.371956	-1.170201
H	5.725716	2.932124	-1.899301
H	-0.001047	1.782850	-1.531417
Cl	8.998295	0.124801	0.297485

Compound 6

C	-2.094853	5.216031	0.415217
C	-2.838536	4.124309	0.839448
C	-2.371135	2.852226	0.507475
C	-0.937032	5.027938	-0.324381
N	-0.497426	3.808230	-0.641212
C	-1.190199	2.756621	-0.232473
C	-0.111442	6.181343	-0.806322
C	-4.098963	4.317749	1.628992
S	-0.548645	1.164493	-0.695239
C	-3.162264	1.635634	0.892130
O	-4.287086	1.436022	0.466965
N	-2.526487	0.798212	1.740728
C	-3.025428	-0.511582	2.100114
C	-2.216307	-1.575442	1.431041
N	-1.091761	-2.010823	1.889848
N	-0.629051	-2.867407	0.940874
C	-1.462847	-2.979290	-0.112171
S	-1.308883	-3.930155	-1.483203
N	-2.493592	-2.132896	0.208031
C	-3.657016	-1.900803	-0.587891
C	-4.864262	-2.453062	-0.194679
C	-3.556650	-1.121542	-1.727870
C	-5.997194	-2.209519	-0.956087
C	-5.909800	-1.433397	-2.102586
C	-4.690791	-0.893261	-2.489078
C	0.677287	-3.469843	1.107752
N	1.712897	-2.479422	0.969195
C	1.891548	-2.030502	-0.401925
C	2.980327	-2.923598	1.524069
C	2.894086	-0.895137	-0.463173
C	3.991161	-1.795128	1.474876
N	4.161273	-1.305497	0.110899
C	5.268901	-0.467199	-0.111737
C	5.162019	0.772429	-0.740641
C	6.550058	-0.888653	0.269405
C	6.290648	1.548462	-0.983882
C	7.557452	1.137106	-0.601665
C	7.659029	-0.101670	0.033242
H	-0.541212	7.134550	-0.503546
H	-0.036720	6.158763	-1.894681
H	0.901535	6.109120	-0.407109
H	-4.174979	5.342679	1.987269
H	-4.137088	3.644291	2.486230
H	-4.969786	4.100945	1.009124
H	-2.418573	6.218663	0.662093
H	-1.609548	1.052398	2.069450
H	-4.913219	-3.064855	0.696811
H	-6.946512	-2.631770	-0.654305
H	-6.795013	-1.247780	-2.696960
H	-4.623466	-0.285215	-3.381499
H	-2.601289	-0.697016	-2.006040
H	-2.956467	-0.651641	3.177221

H	-4.070092	-0.570079	1.804598
H	0.721452	-3.893968	2.110020
H	0.737765	-4.280277	0.371675
H	0.936960	-1.677192	-0.794636
H	2.231873	-2.857034	-1.046884
H	2.830636	-3.221451	2.563187
H	3.380210	-3.791821	0.974936
H	3.651129	-0.980374	2.132819
H	4.939847	-2.159945	1.860255
H	2.475196	-0.019119	0.057606
H	3.043267	-0.624983	-1.508497
H	4.197999	1.155207	-1.044242
H	6.682484	-1.857176	0.734054
H	6.166590	2.505626	-1.478285
H	0.433529	1.669423	-1.451188
C	8.777928	1.977873	-0.856187
H	8.520028	2.898994	-1.378949
H	9.272191	2.248675	0.079465
H	9.508734	1.438931	-1.463136
H	8.634355	-0.465493	0.337870

Compound 7

C	-3.223129	5.123338	0.395004
C	-3.862108	3.976411	0.843796
C	-3.303485	2.745672	0.498648
C	-2.077983	5.026761	-0.381104
N	-1.551510	3.845444	-0.711148
C	-2.143559	2.742919	-0.279084
C	-1.364707	6.241561	-0.890747
C	-5.109750	4.068579	1.670998
S	-1.393317	1.204774	-0.759306
C	-3.978338	1.469597	0.911356
O	-5.085867	1.163948	0.504454
N	-3.260190	0.706287	1.764185
C	-3.639961	-0.635062	2.151482
C	-2.770320	-1.639199	1.466228
N	-1.597282	-1.973289	1.887295
N	-1.101148	-2.804038	0.932129
C	-1.961920	-3.001248	-0.086326
S	-1.781024	-3.957517	-1.450024
N	-3.046266	-2.237509	0.262603
C	-4.255160	-2.117443	-0.489196
C	-5.395775	-2.760116	-0.038631
C	-4.265263	-1.353834	-1.643849
C	-6.574386	-2.624772	-0.756385
C	-6.597340	-1.865114	-1.916903
C	-5.443703	-1.233653	-2.361051
C	0.250821	-3.305192	1.061455
N	1.206589	-2.243668	0.877623
C	1.304063	-1.797736	-0.502124
C	2.522093	-2.595685	1.383791
C	2.215002	-0.590769	-0.608890
C	3.447599	-1.398658	1.299036
N	3.527823	-0.893638	-0.068456
C	4.569714	-0.010110	-0.360838
C	4.369579	1.181549	-1.062369
C	5.881315	-0.331071	0.018615
C	5.437092	2.011048	-1.379159
C	6.724894	1.692044	-0.997952
C	6.929866	0.509407	-0.293807
H	-1.855584	7.157823	-0.567600
H	-1.332043	6.225964	-1.981349
H	-0.334091	6.248656	-0.532470
H	-5.256656	5.083916	2.034501
H	-5.069188	3.393512	2.526876
H	-5.978633	3.784332	1.075913

H	-3.618788	6.097385	0.651355
H	-2.363265	1.043646	2.072979
H	-5.358596	-3.357273	0.863301
H	-7.472621	-3.118280	-0.409704
H	-7.517825	-1.763899	-2.477051
H	-5.462480	-0.638237	-3.264261
H	-3.360641	-0.856142	-1.966894
H	-3.525333	-0.755392	3.226988
H	-4.685480	-0.780807	1.891349
H	0.359747	-3.711080	2.066304
H	0.348098	-4.118713	0.332907
H	0.313732	-1.520008	-0.865245
H	1.683254	-2.603124	-1.151953
H	2.434580	-2.900348	2.427837
H	2.961662	-3.434653	0.819903
H	3.077649	-0.610135	1.970699
H	4.432035	-1.698158	1.647842
H	1.750280	0.260440	-0.087914
H	2.311012	-0.330817	-1.662519
H	3.376338	1.482938	-1.360618
H	6.082769	-1.257383	0.535421
H	7.553260	2.343842	-1.240815
H	5.248547	2.927468	-1.923435
H	-0.477310	1.781822	-1.546130
C	8.331709	0.158915	0.121136
F	9.172101	0.131321	-0.928967
F	8.421283	-1.035135	0.719616
F	8.836025	1.062597	0.982575

Compound 8

C	-2.422889	5.249006	0.519074
C	-3.206621	4.166712	0.892016
C	-2.758393	2.891124	0.546538
C	-1.245204	5.049233	-0.185334
N	-0.824030	3.826526	-0.515132
C	-1.555076	2.783246	-0.154427
C	-0.377104	6.192690	-0.613143
C	-4.488257	4.373754	1.643023
S	-0.934558	1.187193	-0.632998
C	-3.591036	1.686615	0.877006
O	-4.709383	1.529033	0.418727
N	-2.999462	0.810270	1.718287
C	-3.544896	-0.491572	2.036150
C	-2.745681	-1.564899	1.370531
N	-1.655717	-2.050680	1.861317
N	-1.183386	-2.896119	0.906809
C	-1.976938	-2.950502	-0.181821
S	-1.795844	-3.865997	-1.572836
N	-2.991836	-2.079642	0.122229
C	-4.115308	-1.789610	-0.711388
C	-5.355440	-2.306094	-0.376033
C	-3.943675	-0.993109	-1.830714
C	-6.448676	-2.007915	-1.175134
C	-6.289897	-1.213779	-2.301383
C	-5.038689	-0.710171	-2.629839
C	0.089346	-3.553793	1.108753
N	1.166977	-2.598069	1.058866
C	1.413248	-2.088660	-0.278420
C	2.398271	-3.130982	1.612976
C	2.445862	-0.978492	-0.251413
C	3.449311	-2.041803	1.687978
N	3.675101	-1.422481	0.386166
C	4.850983	-0.759175	0.135225
C	4.901806	0.354480	-0.728681
C	6.067634	-1.185816	0.711043
C	6.091460	0.984438	-1.015258

C	7.268481	0.529903	-0.441163
C	7.253765	-0.556054	0.425524
H	-0.791684	7.148674	-0.298257
H	-0.271279	6.195681	-1.699175
H	0.621619	6.084280	-0.187208
H	-4.553765	5.393390	2.018050
H	-4.569464	3.685682	2.485568
H	-5.342400	4.187634	0.990921
H	-2.730707	6.253475	0.778516
H	-2.086340	1.033001	2.078744
H	-5.461056	-2.933226	0.499826
H	-7.423089	-2.402019	-0.918654
H	-7.144270	-0.985639	-2.925331
H	-4.915348	-0.088177	-3.506538
H	-2.964226	-0.597483	-2.063925
H	-3.512703	-0.654918	3.111738
H	-4.581391	-0.514195	1.708500
H	0.072953	-4.018974	2.093482
H	0.156513	-4.335295	0.342772
H	0.488685	-1.684896	-0.692874
H	1.755779	-2.892623	-0.950052
H	2.210396	-3.500761	2.621960
H	2.783655	-3.969257	1.009856
H	3.123548	-1.276377	2.404319
H	4.366038	-2.482424	2.064455
H	2.031987	-0.101689	0.265119
H	2.650724	-0.701322	-1.282810
H	4.001106	0.753782	-1.167024
H	6.095653	-2.040470	1.368685
H	8.175175	-0.908797	0.865028
H	6.106968	1.840978	-1.673160
H	0.082529	1.686860	-1.345061
N	8.516004	1.192385	-0.740830
O	9.533555	0.787064	-0.203841
O	8.504195	2.131150	-1.519666

Compound 9

C	4.582558	4.016701	-0.333408
C	4.894162	2.735462	-0.765322
C	4.092593	1.688046	-0.313437
C	3.515455	4.223283	0.528083
N	2.756032	3.214005	0.960500
C	3.034856	1.987674	0.547641
C	3.152496	5.590536	1.021187
C	6.054084	2.493908	-1.684715
S	1.996308	0.684649	1.166128
C	4.403295	0.271704	-0.698829
O	5.416018	-0.295432	-0.320410
N	3.471764	-0.312859	-1.480942
C	3.465476	-1.722132	-1.814027
C	2.429398	-2.442290	-1.013605
N	1.227684	-2.650827	-1.443020
N	0.574341	-3.218288	-0.396152
C	1.365974	-3.365464	0.685779
S	0.999824	-4.007693	2.191916
N	2.567855	-2.862441	0.280818
C	-0.839633	-3.502418	-0.505804
N	-1.602662	-2.281615	-0.566167
C	-1.625764	-1.560447	0.695445
C	-2.958222	-2.509015	-1.035890
C	-2.325444	-0.225004	0.535188
C	-3.673434	-1.185688	-1.218674
N	-3.669463	-0.411353	0.018504
C	-4.555170	0.665080	0.102524
C	-4.167160	1.920322	0.584319
C	-5.896964	0.491460	-0.267305

C	-5.087909	2.950932	0.698435
C	-6.410939	2.783117	0.328960
C	-6.786075	1.539086	-0.153053
H	3.813627	6.351800	0.610923
H	3.212062	5.623039	2.110292
H	2.124817	5.828537	0.742325
H	6.419664	3.431462	-2.099237
H	5.776168	1.833722	-2.507650
H	6.870319	2.012436	-1.144309
H	5.173591	4.858445	-0.669692
H	2.671902	0.231958	-1.758569
H	3.223126	-1.848565	-2.867133
H	4.461022	-2.123194	-1.639136
H	-0.998311	-4.064892	-1.425213
H	-1.091825	-4.138092	0.351601
H	-0.603506	-1.377379	1.028705
H	-2.132702	-2.148887	1.477579
H	-2.923015	-3.026181	-1.996080
H	-3.530016	-3.136300	-0.332313
H	-3.178840	-0.617794	-2.020350
H	-4.693620	-1.385896	-1.533645
H	-1.725917	0.422356	-0.122860
H	-2.379125	0.246817	1.515531
H	-3.141434	2.110486	0.862087
H	-6.255269	-0.466119	-0.615326
H	-7.127781	3.587525	0.410384
H	-4.758955	3.912116	1.072710
H	1.306194	1.485811	1.986246
C	3.746236	-2.803886	1.122214
H	4.543391	-2.295276	0.587951
H	3.512286	-2.249211	2.029219
H	4.056972	-3.812590	1.388687
Cl	-8.448774	1.276698	-0.617481

Compound 10

C	4.304933	4.411855	-0.413791
C	4.776181	3.157779	-0.774880
C	4.085776	2.045252	-0.296043
C	3.193102	4.530560	0.406763
N	2.539974	3.459842	0.864432
C	2.971591	2.257178	0.518503
C	2.659912	5.866731	0.824662
C	5.986892	3.012359	-1.647811
S	2.068648	0.870106	1.166534
C	4.572892	0.659809	-0.601996
O	5.631126	0.234758	-0.166197
N	3.744062	-0.066621	-1.380704
C	3.926056	-1.475887	-1.658364
C	2.949064	-2.295180	-0.879811
N	1.817946	-2.692013	-1.364746
N	1.186923	-3.302569	-0.328187
C	1.921841	-3.287644	0.802746
S	1.554864	-3.909391	2.316928
N	3.064028	-2.635757	0.440087
C	-0.159316	-3.799261	-0.503001
N	-1.089705	-2.709418	-0.662749
C	-1.295557	-1.953231	0.559816
C	-2.369423	-3.158755	-1.179534
C	-2.163674	-0.736464	0.303504
C	-3.256984	-1.968378	-1.482171
N	-3.426497	-1.112812	-0.312484
C	-4.505636	-0.268724	-0.226710
C	-4.427537	0.963482	0.455739
C	-5.751856	-0.613350	-0.794806
C	-5.525634	1.783299	0.581987
C	-6.736575	1.405880	0.022457

C	-6.846448	0.205893	-0.669332
H	3.246448	6.680777	0.402499
H	2.673251	5.950878	1.912511
H	1.623550	5.974364	0.500714
H	6.251711	3.965211	-2.102188
H	5.817988	2.282445	-2.440879
H	6.835938	2.661178	-1.059685
H	4.806376	5.301009	-0.772855
H	2.899824	0.367885	-1.715571
H	3.754851	-1.667323	-2.715719
H	4.953502	-1.743126	-1.422313
H	-0.176534	-4.413198	-1.402634
H	-0.368317	-4.432372	0.367734
H	-0.335030	-1.610074	0.945440
H	-1.762952	-2.579781	1.336989
H	-2.210587	-3.715644	-2.104094
H	-2.882169	-3.824423	-0.465920
H	-2.811437	-1.385623	-2.298823
H	-4.216158	-2.341814	-1.823642
H	-1.620433	-0.020101	-0.327291
H	-2.354201	-0.267020	1.265595
H	-3.494298	1.302936	0.875884
H	-5.879398	-1.547620	-1.318650
H	-7.793907	-0.084617	-1.098904
H	-5.440946	2.726085	1.102254
H	1.272807	1.619620	1.938152
C	4.168451	-2.374785	1.341386
H	4.929745	-1.802255	0.819595
H	3.809340	-1.800466	2.193668
H	4.584030	-3.317383	1.693064
N	-7.888264	2.266506	0.153674
O	-7.770728	3.305080	0.782722
O	-8.934993	1.921210	-0.369207

Compound 11

C	4.175678	4.118733	-0.311863
C	4.540587	2.841187	-0.711657
C	3.760451	1.776095	-0.263769
C	3.078179	4.304945	0.515484
N	2.340036	3.278814	0.945142
C	2.669991	2.055311	0.562397
C	2.656860	5.667834	0.973066
C	5.733430	2.621480	-1.593637
S	1.657954	0.729132	1.174836
C	4.126013	0.364551	-0.616993
O	5.142556	-0.165854	-0.197626
N	3.238553	-0.258821	-1.419949
C	3.289818	-1.670416	-1.738517
C	2.254060	-2.420492	-0.965670
N	1.076094	-2.677467	-1.432896
N	0.410943	-3.265452	-0.405111
C	1.171418	-3.376574	0.703036
S	0.781999	-4.027493	2.199811
N	2.365578	-2.828480	0.335196
C	-0.987919	-3.601503	-0.560265
N	-1.792318	-2.409662	-0.642235
C	-1.891870	-1.701509	0.623586
C	-3.117190	-2.677531	-1.175422
C	-2.636896	-0.393578	0.444514
C	-3.872498	-1.377342	-1.366294
N	-3.954102	-0.631915	-0.114656
C	-4.866487	0.438451	-0.089889
C	-4.513467	1.720121	0.330212
C	-6.201538	0.226650	-0.460385
C	-5.458967	2.739002	0.384799
C	-6.777709	2.533822	0.012077

C	-7.125482	1.250938	-0.413549
H	3.302468	6.443613	0.565276
H	2.687064	5.722212	2.062515
H	1.629354	5.865554	0.663792
H	6.085059	3.563908	-2.009128
H	5.498053	1.944162	-2.415985
H	6.546254	2.169163	-1.023680
H	4.748688	4.973440	-0.646685
H	2.432732	0.257926	-1.731881
H	3.086362	-1.814220	-2.797616
H	4.292138	-2.036039	-1.528183
H	-1.095144	-4.166605	-1.485402
H	-1.243403	-4.249177	0.287171
H	-0.890407	-1.484689	0.997520
H	-2.405776	-2.316582	1.380389
H	-3.019708	-3.176743	-2.140976
H	-3.695728	-3.337373	-0.507973
H	-3.363418	-0.776352	-2.135694
H	-4.871310	-1.605164	-1.729676
H	-2.038247	0.276200	-0.193312
H	-2.741311	0.077935	1.421548
H	-3.495708	1.945987	0.614847
H	-6.526421	-0.759797	-0.764958
H	-8.150504	1.045545	-0.702688
H	-5.145405	3.721522	0.719996
H	0.915941	1.520830	1.957896
C	3.511798	-2.718821	1.214974
H	4.305834	-2.182097	0.703983
H	3.225941	-2.168974	2.109991
H	3.852990	-3.712890	1.498688
C	-7.799635	3.635853	0.060910
H	-7.365728	4.559123	0.445031
H	-8.204650	3.842038	-0.932361
H	-8.640926	3.365792	0.702889

Compound 12

C	-1.922626	5.126920	0.503085
C	-2.854149	4.139172	0.790116
C	-2.643035	2.873584	0.245642
C	-0.833354	4.845036	-0.307849
N	-0.642828	3.632201	-0.832465
C	-1.519505	2.678261	-0.559738
C	0.193406	5.884199	-0.640169
C	-4.043614	4.428358	1.656495
S	-1.227150	1.089499	-1.297179
C	-3.628915	1.767621	0.487479
O	-4.755018	1.786088	0.016512
N	-3.156973	0.766392	1.257226
C	-3.856972	-0.479920	1.483424
C	-3.015676	-1.618633	0.991387
N	-1.891419	-1.899766	1.564293
N	-1.344961	-2.882929	0.808508
C	-2.127987	-3.226527	-0.231491
S	-1.834744	-4.389760	-1.410343
N	-3.218873	-2.406175	-0.110531
C	-0.021855	-3.374175	1.130967
N	0.965338	-2.348481	0.914463
C	1.182083	-2.050971	-0.490985
C	2.229030	-2.656926	1.560387
C	2.115760	-0.866864	-0.644083
C	3.177933	-1.482259	1.431083
N	3.376359	-1.117486	0.031442
C	4.445005	-0.264991	-0.257451
C	4.311237	0.848638	-1.094008
C	5.715035	-0.540440	0.269705
C	5.406815	1.643639	-1.395364

C	6.659846	1.376288	-0.872520
C	6.782710	0.275446	-0.039464
H	-0.034514	6.837374	-0.166359
H	0.241109	6.030920	-1.720401
H	1.179915	5.554076	-0.310797
H	-3.938713	5.393474	2.148403
H	-4.171361	3.659859	2.420455
H	-4.954143	4.442158	1.055729
H	-2.044398	6.120121	0.914988
H	-2.223541	0.845253	1.626772
H	-4.044711	-0.620423	2.547851
H	-4.813710	-0.421162	0.976822
H	-0.015865	-3.654877	2.183686
H	0.129256	-4.271229	0.519097
H	0.229192	-1.803929	-0.960978
H	1.601510	-2.922193	-1.020184
H	2.052666	-2.851880	2.619436
H	2.698719	-3.553276	1.123041
H	2.768127	-0.627159	1.988560
H	4.126396	-1.752986	1.886560
H	1.620675	0.034826	-0.252784
H	2.301010	-0.716087	-1.707043
H	3.349895	1.114738	-1.507061
H	5.877869	-1.405468	0.895755
H	5.273810	2.500150	-2.044122
H	-0.158418	1.496539	-1.992521
C	-4.371309	-2.446328	-1.025128
H	-4.117902	-3.262748	-1.700242
C	-5.650575	-2.823105	-0.291753
H	-5.997514	-2.038200	0.380292
H	-6.436872	-2.998835	-1.025742
H	-5.509451	-3.737319	0.285077
C	-4.475221	-1.168715	-1.843342
H	-3.534636	-0.965293	-2.355991
H	-5.255520	-1.293671	-2.594431
H	-4.732507	-0.301555	-1.234398
H	7.512883	1.999004	-1.100842
Cl	8.348251	-0.108522	0.631794

Compound 13

C	-1.692779	5.236000	0.566288
C	-2.727421	4.333667	0.767688
C	-2.579569	3.051457	0.240180
C	-0.565546	4.858153	-0.147941
N	-0.434162	3.630361	-0.655250
C	-1.409408	2.755876	-0.462076
C	0.570305	5.804900	-0.388669
C	-3.957877	4.730700	1.527646
S	-1.183434	1.143121	-1.172531
C	-3.679421	2.040554	0.385807
O	-4.764653	2.180192	-0.155483
N	-3.358758	0.978846	1.152507
C	-4.193496	-0.193898	1.302645
C	-3.437493	-1.405032	0.846958
N	-2.405930	-1.821612	1.505420
N	-1.901229	-2.838960	0.765924
C	-2.619284	-3.068710	-0.349956
S	-2.345174	-4.227215	-1.537679
N	-3.622635	-2.137218	-0.295793
C	-0.678345	-3.485839	1.189618
N	0.437643	-2.580100	1.085089
C	0.816884	-2.298793	-0.287764
C	1.594732	-3.050415	1.824132
C	1.886955	-1.226064	-0.341533
C	2.678984	-1.991717	1.824132
N	3.039289	-1.589793	0.468181

C	4.249797	-0.984504	0.233667
C	4.409209	-0.020244	-0.783198
C	5.395415	-1.325628	0.985330
C	5.635254	0.547369	-1.045749
C	6.740811	0.178996	-0.295095
C	6.617796	-0.757735	0.723747
H	0.382758	6.777246	0.063402
H	0.724532	5.939242	-1.460503
H	1.492942	5.395746	0.026045
H	-3.808061	5.681313	2.035957
H	-4.224531	3.976670	2.269766
H	-4.805548	4.829920	0.848271
H	-1.763947	6.238003	0.968608
H	-2.453413	0.960738	1.593060
H	-4.469148	-0.330550	2.348303
H	-5.101491	-0.031766	0.732681
H	-0.798813	-3.779324	2.231796
H	-0.576578	-4.385909	0.572224
H	-0.051589	-1.940696	-0.841979
H	1.182473	-3.208834	-0.790878
H	1.307283	-3.250302	2.857378
H	1.995552	-3.983935	1.396360
H	2.325938	-1.115181	2.382935
H	3.541961	-2.393617	2.343901
H	1.464067	-0.267895	-0.010484
H	2.191231	-1.123512	-1.380633
H	3.564291	0.312941	-1.364490
H	5.340330	-2.066070	1.767884
H	7.484332	-1.044105	1.301419
H	5.733537	1.290022	-1.823897
H	-0.039411	1.466647	-1.787420
C	-4.690861	-2.034077	-1.303190
H	-4.458962	-2.848185	-1.988640
C	-6.056871	-2.310144	-0.691551
H	-6.379690	-1.522960	-0.010104
H	-6.793688	-2.375806	-1.491997
H	-6.052337	-3.256821	-0.150968
C	-4.605296	-0.726248	-2.074249
H	-3.607087	-0.593075	-2.492215
H	-5.320614	-0.755189	-2.896509
H	-4.840826	0.140701	-1.456566
N	8.026305	0.777140	-0.568844
O	8.111904	1.580202	-1.483015
O	8.976967	0.455562	0.124596
